# A Protein-Centric Mass Spectrometry Approach for Species Identification within Harmful Algal Blooms

**DOI:** 10.1021/jacs.5c07419

**Published:** 2025-07-28

**Authors:** Jaspreet K. Sound, Hannah E. Wedgwood, Qonita Afinanisa, Tim W. Overton, Aneika C. Leney

**Affiliations:** † School of Biosciences, 1724 University of Birmingham, Edgbaston, Birmingham B15 2TT, U.K.; ‡ School of Chemical Engineering, University of Birmingham, Edgbaston, Birmingham B15 2TT, U.K.

## Abstract

Harmful algal blooms present severe environmental threats, impacting water quality, aquatic ecosystems, and human health. The frequency and intensity of these blooms are rising, largely driven by global warming and changing climatic conditions. There is an urgent need for innovative methods to monitor blue-green algae, also known as cyanobacteria, to enable the implementation of preventative measures. Here, we show that native mass spectrometry is an effective tool for detecting cyanobacteria directly from lake samples, both prior and during bloom formation. Our approach allows for the rapid characterization of cyanobacterial populations within lakes, offering valuable insights into the dynamics of cyanobacterial species associated with harmful algae blooms. Overall, we highlight the exceptional capability of native mass spectrometry in directly detecting and monitoring cyanobacterial blooms, which will support the development of more effective strategies to mitigate this growing environmental challenge.

## Graphical abstract

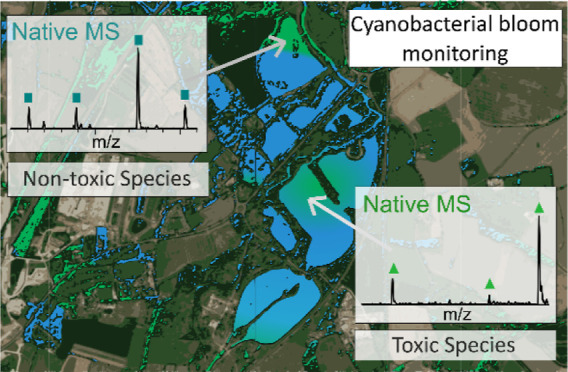

## Introduction

Cyanobacteria (also known as blue-green algae) are ubiquitous micro-organisms commonly found in ponds, lakes and oceans, among other ecosystems, worldwide. Due to their ability to perform extremely efficient oxygenic photosynthesis,^1^ cyanobacteria have been crucial in the formation and maintenance of Earth’s atmosphere^2^ and are essential to the carbon^3^ and nitrogen cycle.^4^ However, cyanobacteria can also accumulate into cyanobacterial blooms. Incidences of these blooms are increasing worldwide due to climate change and eutrophication,^5–7^ and are having devastating effects on water quality.^8,9^ The primary concern regarding cyanobacterial blooms is their toxicity. Besides, some but not all strains of cyanobacteria produce cyanotoxins, as secondary metabolites, which can cause hepatotoxicity, neurotoxicity and dermatotoxicity in both animals^10^ and humans.^11–13^ Characterizing harmful cyanobacterial blooms is challenging since cyanotoxin production is strain specific and cyanotoxin release can occur as a result of a range of environmental and biological factors. New methods are critical to unlock our ability to both monitor cyanobacterial strain presence and quantify any toxins present.

Within a single body of water, it is estimated that many species of cyanobacteria coexist,^14,15^ alongside other micro-organisms and biological material. Although their presence can be established visibly upon bloom formation, either by the naked eye or using technologically advanced remote sensing, different cyanobacteria strains are often indistinguishable even by microscopy analysis. Flow cytometry can detect cyanobacteria at low abundance prior to bloom formation, although again, it is unable to discriminate between similar strains and analysis is challenged by multicellular conformations such as colonies and filaments. In contrast, cyanobacterial identification based on 16S RNA sequencing is advancing rapidly, and while it can determine the presence of toxin producing genes, it is unable to detect or quantify any released toxins. Liquid chromatography–mass spectrometry (LC–MS) has become the gold standard approach for cyanotoxin analysis, being able to detect the most common microcystin cyanotoxin variant, MC-LR, at levels below the drinking water limit of 1 μg/L set by the World Health Organization (WHO).^16^ Furthermore, the application of tandem mass spectrometry (MS/MS) allows for specific cyanotoxins to be identified. We have demonstrated previously that mass spectrometry, in the form of native MS, can also be applied to the identification of individual lab-grown cyanobacterial strains and that even upon mixing together, strains were distinguishable from one another.^17^ This approach is based upon the fact that almost all cyanobacteria contain light harvesting complexes, termed phycobilisomes, that are divided into subcomplexes predominantly comprising the phycobiliproteins phycocyanin and allophycocyanin. Across cyanobacteria, phycobiliproteins maintain the same function but have subtle differences in mass due to their differing primary sequences. Thus, by utilizing high-resolving native MS, different strains of cyanobacteria could be clearly resolved. This approach requires minimal sample preparation yet maintains high sensitivity. Thus, using our native MS approach, detecting low abundances of cyanobacteria prior to bloom formation is highly feasible.

Here, we aimed to develop an innovative method that can identify low abundant toxin-producing cyanobacterial strains directly from lake water while simultaneously quantifying their cyanotoxin production.

## Results and Discussion

First, we chose to analyze a lake where a visible cyanobacterial bloom was present, herein called Lake Water 1 (Figure S1 and Table S1). Flow cytometry confirmed the presence of cyanobacteria (Figures 1a and S2a), characterized by higher phycocyanin (λ_ex_ 640 nm, λ_em_ 675 nm) than chlorophyll (λ_ex_ 488 nm, λ_em_ > 670 nm) fluorescence. Furthermore, light microscopy identified colonies of unicellular cyanobacteria (Figures 1b and S2b), likely corresponding to strains from the common bloom-forming genus *Microcystis*. Consistent with this observation, previous flow cytometry analysis^18^ showed that *Microcystis* spp. possess very high phycocyanin fluorescence, corresponding to the major cyanobacterial population observed by flow cytometry in Lake Water 1 (Figure S2a). To clarify the cyanobacterial species present, the lake water was filtered using a 0.2 μm PES membrane, and any retained cellular material was lysed using a combination of freeze–thaw cycles and sonication (see Supporting Information methods). Successful cell lysis was confirmed by a blue-green colored cell lysate with clear spectroscopic features corresponding to the presence of phycobilisome components over the 500–700 nm range,^19,20^ in addition to chlorophyll a (λ_max_ = 430, 662) and b (λ_max_ = 453, 642)^21,22^ from either cyanobacteria or other photosynthetic organisms^23^ (Figure 1c). The lysate was filtered through a 30 kDa molecular weight filter and the analytes >30 kDa were analyzed by high-resolving native MS. Strikingly, despite the range of proteins from different organisms that could have been present, relatively few charge state distributions were observed in the 4500–5500 *m*/*z* region (Figures 1d and S3) corresponding to proteins of 106–113 kDa in mass. In addition, we noted that the abundant peaks observed in the 2800–3800 *m*/*z* region corresponded to proteins of 35.5–37.5 kDa, approximately one-third of the mass of the larger analytes (Figure S3).

**Figure 1:**
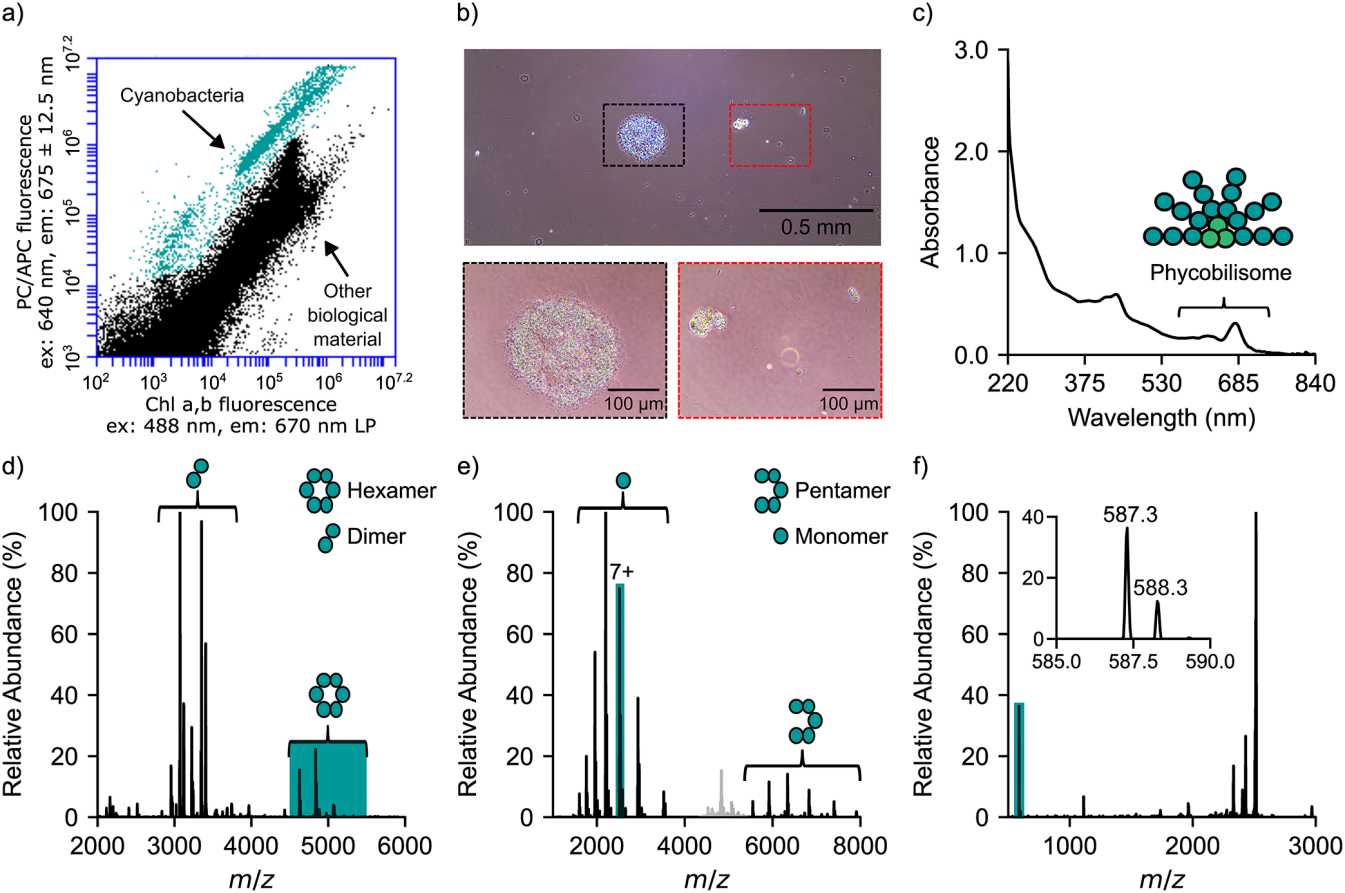
Cyanobacteria identification in lake water 1. Lake water 1 was analyzed directly by flow cytometry (a) and light microscopy (b). The lake water was filtered, the cellular material lysed, and the soluble lysate analyzed by UV–vis spectroscopy (c) confirming the presence of light harvesting complexes. Native MS of the cell lysate (d) showed charge state distributions corresponding to the hexameric and dimeric forms of phycobiliproteins. The hexameric region (blue, panel d) was selected for MS^2^ fragmentation (e) to produce charge state distributions corresponding to the monomeric and pentameric forms of phycobiliproteins. The monomeric 7+ charge state (green, panel e) was selected for MS^3^ fragmentation (f) to detect the phycocyanobilin chromophore.

Consistent with previous data on pure, lab-grown cyanobacterial cultures,^17^ we hypothesized that the protein features in the native MS correspond to dimers and hexamers of the phycobiliprotein complexes, phycocyanin and allophycocyanin (Figure S4). Hexameric phycobiliprotein complexes are composed of three αβ heterodimers. To confirm the presence of these hexameric protein complexes, the 4500–5500 *m*/*z* region of the native mass spectrum was selected and the ions subjected to higher-energy collision induced dissociation (MS^2^) (Figures 1e and S5a). Peaks corresponding to pentameric and monomeric subcomplexes were detected, as is typical for asymmetric charge partitioning during collision induced dissociation of hexameric protein complexes.^24–26^ Phycobiliproteins are unique in that they contain bilin chromophores, termed phycocyanobilin, to aid their function in light energy transfer (Figure S4). To further confirm phycobiliprotein presence, the released monomer from the MS^2^ experiment was selected for further fragmentation (MS^3^). Dominant peaks were observed in the MS^3^ spectrum at 587.3 *m*/*z* consistent with phycocyanobilin release (Figures 1f and S5b,c).

To determine the identity of species present within the lake, we searched the mass of phycocyanin and allophycocyanin dimers obtained from the native mass spectrum (Figures 1d and S3b) against all possible sequences of phycobiliprotein αβ complexes within the UniProt database.^27^ Of eight clearly resolved peaks within the 2800–3800 *m*/*z* region (Figures 2a and S3b), seven were mass matched to phycobiliprotein αβ dimers from known species (Table S3). Notably, the 11+ charge state ions corresponding to the 35,578 Da and 35,667 Da complexes, match exactly (within 1 Da) to the masses of allophycocyanin dimers from the *Microcystis* genus, with one of the most abundant monomers in the MS^3^ spectrum at 17,774 Da corresponding to the α subunit of allophycocyanin from the same genera (Figures 1e, S5a and Table S4). In addition to allophycocyanin, the peak associated with a 37,457 Da complex matched the theoretical molecular weight of the phycocyanin dimer from the species (Figures 2a, S3b and Table S3). To verify the native MS findings, the proteins present within the native MS sample were digested with trypsin and the resulting peptides were analyzed by liquid chromatography (LC)–MS/MS. Peptides were detected corresponding to 27 strains (see Supporting Information), confirming a strong *Microcystis* presence in Lake Water 1.

**Figure 2:**
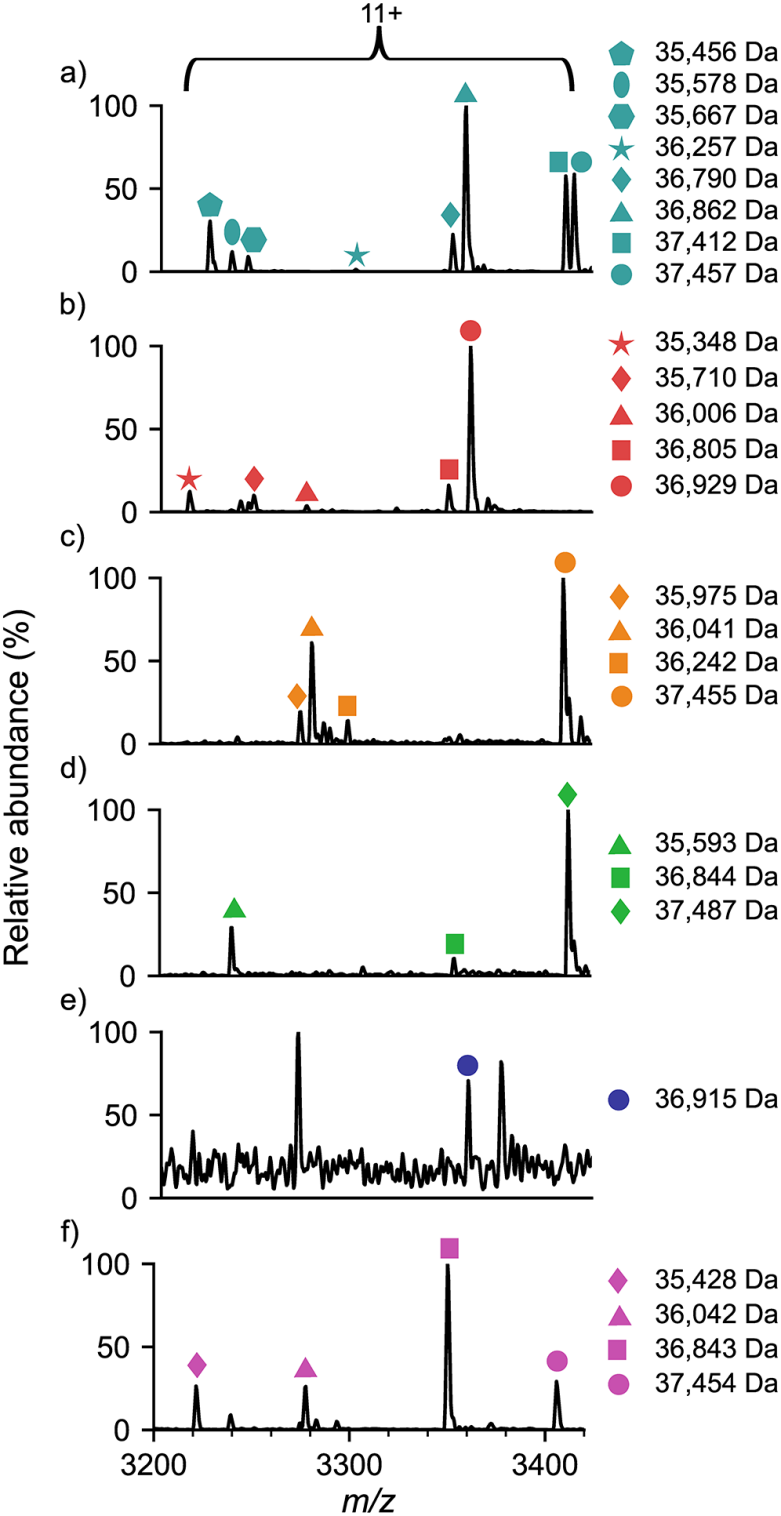
Cyanobacteria fingerprints differ across lakes from different geographical regions. Native MS of the 3200–3400 *m*/*z* region for lake water 1 (a), lake water 2 (b) lake water 3 (c), lake water 4 (d), lake water 5 (e), and lake water 6 (f). Peaks corresponding to the 11+ charge state of phycobiliprotein dimer-like masses are highlighted, each with a different symbol. The complex masses were compared to theoretical masses from all cyanobacterial strains (Table S3) to confirm their identity.

We next sought to determine whether our native MS approach could detect variances in cyanobacterial profiles across different lakes. Samples were collected from 5 additional lakes (Lake Water 2–6) within a 35 km radius, each geographically separated by at least 9 km (Table S1). Flow cytometry analysis of the lakes (Figures S6a–S10a and Table S1) showed the presence of cyanobacteria to varying degrees, both above and below the WHO guideline of 100,000 cells/mL for moderate risk of adverse health effects.^28^ Furthermore, light microscopy showed the presence of cyanobacterial features across all lakes (Figures S6b–S10b). Upon cell lysis, phycobilisome features were observed in all lake water as determined by UV–vis spectroscopy (Figure S11). In addition, the native MS data showed clear peaks corresponding to cyanobacterial complexes (Figures 2, S12–S16 and Table S3). Moreover, fragmentation of these complexes confirmed hexameric structure (Figures S17a–S21a and Table S4) and bilin presence within the complex (Figures S17b–S21b).

Strikingly, although generated from similar environments, the native mass spectra show stark differences in the cyanobacterial profiles of the different lakes based on the phycobiliprotein complexes detected (Figure 2). For example, the number of complexes ranged from 1 in Lake Water 5 (Figure 2e) to 8 in Lake Water 1 (Figure 2a), highlighting varied cyanobacterial presence across the lakes. Furthermore, the observed masses of the phycobiliprotein complexes from each lake indicate an array of originating cyanobacteria. Lake Water 4 is dominated by a phycobiliprotein dimer of 37,487 Da (Figure 2d, green diamond) which is absent from all other lakes. The mass of this complex matches phycocyanin from three potential species; *Chroococcidiopsis cubana*, *Synechocystis* sp. and *Nostoc* sp. (Table S3). To uncover the exact species present, matches at the monomer level were also considered. The predominant monomer in Lake Water 4, released by MS^3^, is 17,774 Da (Figure S19a) which corresponds to the allophycocyanin β subunit of *Nostoc* sp. with no monomers from *Chroococcidiopsis cubana* or *Synechocystis* sp. detected (Table S4). Together this indicates that Lake Water 4 is dominated by species of *Nostoc* origin, contrasting to Lake Water 1 which consists of mainly *Microcystis* (Figure 2). Despite their different relative abundances, identical cyanobacterial species are also observed between the lake samples. For example, the abundant peak in Lake Water 6 at 36,843 Da (Figure 2f, pink square) overlaps in *m*/*z* with a peak in Lake Water 4 (Figure 2d, green square). These peaks correspond to the phycocyanin dimer from the toxin-producing species *Anabaena* sp. (Table S3) and both lakes also indicate the presence of this species at the monomer level (Table S4). In addition, phycobiliproteins associated with detected in Lake Water 1 (Figure 2a, cyan circle) are also detected in Lake Water 3 (Figure 2c, orange circle) and Lake Water 6 (Figure 2f, pink circle) in the dimeric and monomeric forms (Tables S3 and S4). In many cases, the native MS data is consistent with the light microscopy analysis whereby a variety of cyanobacterial features are observed across the lake samples (Figures S2b, S6b–S10b). However, in some cases the species identified by native MS is less defined by microscopy. For example, a strong presence of *Nostoc* and *Anabaena* species in Lake Water 4 is determined by native MS, but these species’ features are not obviously visible by microscopy (Figure S8b). This could be due to unintentional selective sampling during microscopy, which is lessened in native MS due to the unbiased approach to sample collection and analysis and the greater amount of material taken for analysis. Flow cytometry is a rapid technique that can be used to detect unicellular cyanobacteria and other phytoplankton^29^ analysis of filamentous (such as *Anabaena*) and colonial (such as *Microcystis* spp.) organisms can be problematic. Indeed, previous studies^29^ have highlighted the need for flow cytometry to be used in conjunction with other techniques. This is corroborated by the fact that flow cytometry shows a population of cyanobacteria present in lake water 5 (Figure S9) comparable to lake water 1 (Figure S2), yet only one species dominates in the native mass spectrum (Figure 2e). Furthermore, lake water 5 likely contains a highly diverse set of cyanobacteria in lower abundance based off the high number of proteins detected by LC–MS/MS (see Supporting Information).

Finally, we sought to find out whether the cyanobacteria profiles uncovered by native MS could be used to predict cyanotoxins in lake samples. To achieve this, the samples Lake Water 1–6 were analyzed for the presence of cyanotoxins. Filtered lake water from each lake was passed through a solid phase extraction cartridge and any bound toxin was eluted for further analysis by LC–MS/MS. Seven major toxins; microcystin-RR, -YR, -LR, -LA, -LY, -LW, and nodularin-R, were quantified (Figures S22–S24). Our native MS data showed evidence of toxin producing genera such as *Microcystis* and/or *Anabaena* across all lakes (Tables S3 and S4). LC–MS/MS determined quantifiable amounts of microcystin in only Lake Water 6 (Table S5), indicating that although microcystin-producing species were present in all the other lakes, they were not actively releasing quantities that were detrimental to the lake water quality. Similarly, no nodularin-R was detected across the lakes, including the *Nostoc* containing Lake Water 4 (Table S5).

Within Lake Water 6, four different microcystin variants were detected and quantified (Figure S25 and Table S4) including the heavily studied microcystin-LR and –RR (Figure 3b,c). In particular, levels of microcystin-LR reached 3.45 μg/L, surpassing the WHO guideline for safe drinking water but remaining below the guideline of 24 μg/L for recreational water.^16^ The presence of microcystin is supported by the identification of both non-N_2_-fixing *Microcystis* and N_2_-fixing *Anabaena* species by native MS (Figure 2, Tables S3 and S4) which are collectively among the most toxic cyanobacterial genera. Moreover, light microscopy clearly identifies the presence of *Microcystis* (Figure 3a) with some filamentous features that could be affiliated with *Anabaena* also observed (Figure S2b). Consistent with these findings, it has been suggested that *Microcystis* and *Anabaena* succeed each other during harmful algal blooms and have an allelopathic interaction between one another dependent upon their aquatic environment.^30–32^ This species–species interaction could explain the greater detection of *Microcystis* compared to *Anabaena* in Lake Water 6. According to standards set by the WHO, the high level of cyanobacterial cells in Lake Water 6, as determined by flow cytometry (Figure S10a and Table S1), is deemed a high-risk bloom.^28^ Using protein-based mass spectrometric approaches, we have confirmed that the composition of the bloom contains harmful cyanobacteria and corresponding toxins, highlighting the requirement for management.

**Figure 3:**
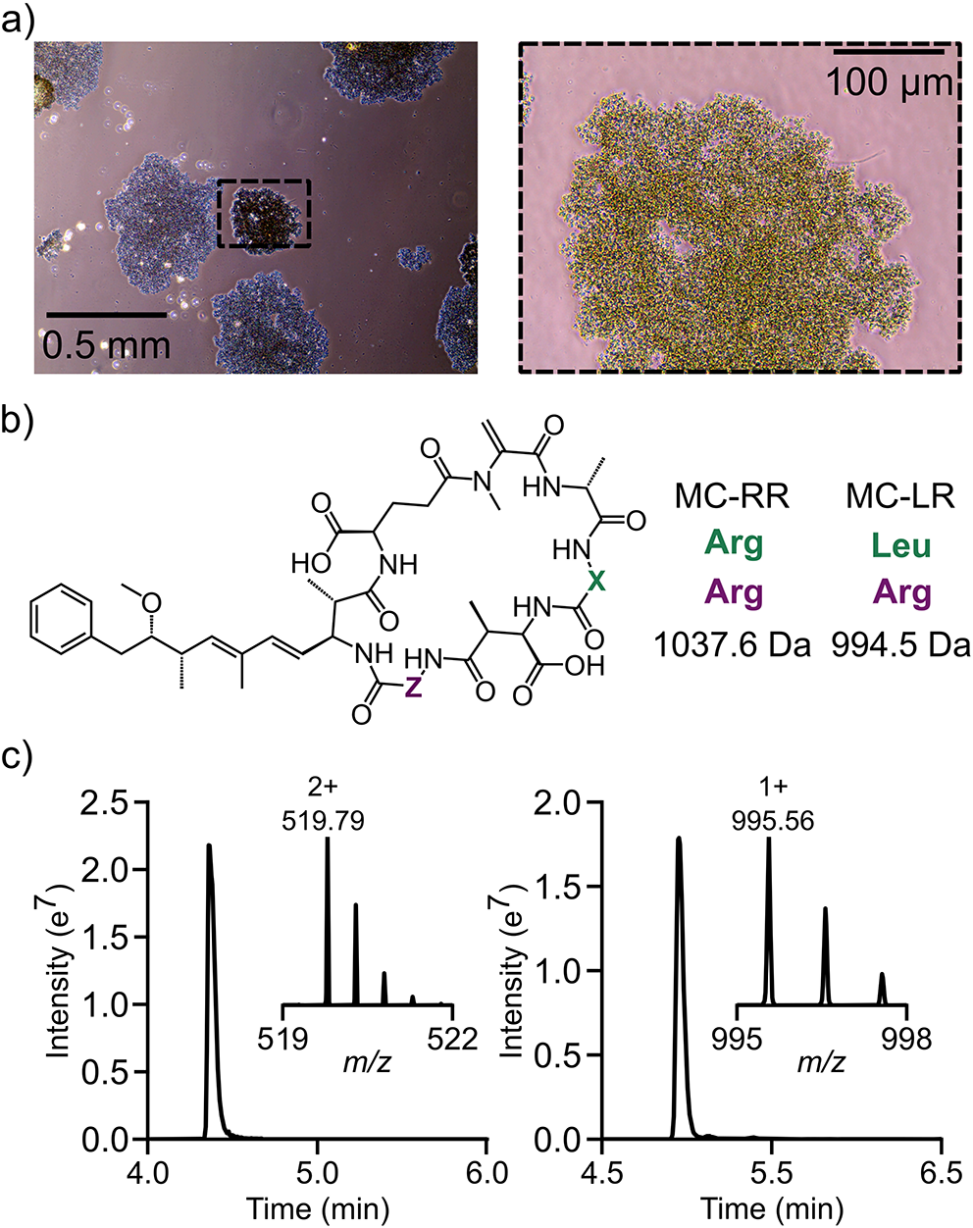
Cyanotoxins detected corroborate with species detected in lake water. (a) Light microscopy of lake water 6 corroborates the presence of the cyanobacterium , (b) Chemical structure of microcystin where X/Z corresponds to arginine/arginine or leucine/arginine for microcystin-RR (MC-RR) and microcystin-LR (MC-LR), respectively. Corresponding monoisotopic masses of MC-RR and MC-LR are indicated. (c) Liquid chromatogram and corresponding mass spectrum of the [M+2H]^2+^ ion of MC-RR (c, left) and the [M + H]^+^ ion of MC-LR (c, right) detected within lake water 6.

## Conclusions

In summary, we have shown that native MS can identify cyanobacterial proteins directly from freshwater lakes. By molecular weight comparison with the UniProt database, complex dissociation and bottom-up proteomics we identify these proteins to be the phycobiliproteins from cyanobacteria’s highly abundant light harvesting complex, the phycobilisome. Comparing the six different lakes analyzed, the native mass spectra differ dramatically in their phycobiliprotein spectral features, highlighting the ability of high-resolving native MS to not only determine which cyanobacterial strains are present, but also the range of cyanobacterial strains that can coexist in different environments. Finally, we show the high sensitivity of these native MS measurements in detecting cyanobacteria prior to levels whereby toxins released exceed the WHO guidelines for recreational water use.

Although many strains were identified precisely based off their accurate mass (Tables S3 and S4), our approach is heavily reliant on genome sequences from cyanobacterial databases. Inaccuracies in these sequences can result in false negative identifications. In addition, some phycobiliprotein-like masses were not matched to any theoretical sequence, highlighting the need for more extensive coverage of the cyanobacterial taxonomy in bioinformatic resources which can be addressed through advances in metagenomics. Moreover, these masses could correlate to cryptophytes,^33–35^ glaucophytes^36^ or rhodophytes^37–40^ that also contain phycobiliproteins but are not reported to produce toxins. Furthermore, in freshwater lake samples, it can be assumed that cyanobacteria are more abundant than other organisms such as red algae which are typically found in marine environments, making it more likely that the detected phycobiliproteins are indeed of cyanobacterial origin. Regardless, our native MS approach can readily generate unique profiles of lake water samples containing key information that can be highly valuable in understanding harmful algal blooms.

Overall, our data highlights the power of native MS to rapidly identify cyanobacteria directly from lake water. In particular, we have shown that toxic cyanobacterial species can be detected, even before the release of corresponding toxins, highlighting the potential for our native MS method to monitor and predict harmful algal blooms. Moreover, we envisage this approach will be readily applied to longitudinal monitoring of cyanobacterial strains in any water body.

## Supplementary Material







## Data Availability

All mass spectrometry data has been deposited to the ProteomeXchange Consortium via the PRIDE partner repository with the data set identifier PXD065745 and 10.6019/PXD065745.

## Notes

Experimental details and data (PDF)
Dimer and monomer phycobiliprotein annotations (XLSX)
Proteomics species identification (PDF)

## Abbreviations

LC: liquid chromatography
MC: microcystin
MS: mass spectrometry
WHO: world health organization.
