# Supplementary material for: A Protein-Centric Mass Spectrometry Approach for Species Identification within Harmful Algal Blooms
Source: J Am Chem Soc. 2025 Jul 28;147(31):27974–80. doi: 10.1021/jacs.5c07419 (PMC12333325; doi:10.1021/jacs.5c07419)
Supplement: Supplementary file 1 [file ja5c07419_si_001.pdf]

Supporting Information

A protein-centric mass spectrometry approach for species identification within harmful algal blooms.

Jaspreet K. Sound[†], Hannah E. Wedgwood[†], Qonita Afinanisa[‡], Tim W. Overton[‡], Aneika C. Leney[†*]

[†] School of Biosciences, University of Birmingham, Edgbaston, Birmingham, B15 2TT, UK

[‡] School of Chemical Engineering, University of Birmingham, Edgbaston, Birmingham, B15 2TT, UK

* a.leney@bham.ac.uk

CONTENTS

Materials and Methods 3
Water Sample Collection and Processing 3
Light Microscopy 3
Flow Cytometry 3
Protein Extraction 3-4
Native Mass Spectrometry 4
Identification of Cyanobacteria in Lake Water 4-5
Bottom-up proteomics LC-MS analysis 5-6
Cyanotoxin extraction 6
Cyanotoxin LC-MS analysis 6-7
References........ 7
Supplementary Figures 8
Figure S1: Photograph of cyanobacterial bloom during collection of lake water 1 8
Figure S2: Flow cytometry and microscopy of lake water 1 9
Figure S3: Native mass spectrometry of the lysate from lake water 1 10
Figure S4: Hexameric structures of allophycocyanin and phycocyanin........ 11
Figure S5: Tandem native mass spectrometery of the lysate from lake water 1 11
Figure S6: Flow cytometry and microscopy of lake water 2 12
Figure S7: Flow cytometry and microscopy of lake water 3 13
Figure S8: Flow cytometry and microscopy of lake water 4 14
Figure S9: Flow cytometry and microscopy of lake water 5 15
Figure S10: Flow cytometry and microscopy of lake water 6 16
Figure S11: Absorbance spectra of lysed lake material 17
Figure S12: Native mass spectrometry of the lysate from lake water 2 18
Figure S13: Native mass spectrometry of the lysate from lake water 3 19
Figure S14: Native mass spectrometry of the lysate from lake water 4 20
Figure S15: Native mass spectrometry of the lysate from lake water 5 21
Figure S16: Native mass spectrometry of the lysate from lake water 6 22
Figure S17: Tandem native mass spectrometry of the lysate from lake water 2 23
Figure S18: Tandem native mass spectrometry of the lysate from lake water 3 24
Figure S19: Tandem native mass spectrometry of the lysate from lake water 4 25
Figure S20: Tandem native mass spectrometry of the lysate from lake water 5 26
Figure S21: Tandem native mass spectrometry of the lysate from lake water 6 27

Figure S22: Liquid chromatography of cyanotoxin standards 27
Figure S23: Mass spectra of cyanotoxins 28
Figure S24: Calibration curves of cyanotoxins 29
Figure S24: LC-MS of extracted cyanotoxins 30
Supplementary Tables 31
Table S1: Location of lake samples taken for analysis 31
Table S2: Instrument parameters for Native Mass Spectrometry analysis 31
Table S3: Observed phycobiliprotein dimers matched to known strains 32-33
Table S4: Observed phycobiliprotein monomers matched to known strains 34-38
Table S5: Concentration of cyanotoxins in lake water samples 39
Table S6: Inclusion list for triggering MS2 data acquisition 39

MATERIALS AND METHODS

Water Sample Collection and Processing

Lake water samples were collected from 6 sites (1 L per site in borosilicate glass bottle) across Warwickshire, Leicestershire, and Nottinghamshire, UK in August-September 2024 (Table S1). These water bodies are utilized for recreational purposes or water supply reservoirs. Water samples were collected ~10 cm below the surface. All lakes were >9 km apart. The Global Positioning System (GPS) coordinates are available upon request. Lake samples were stored at 21 °C, maintaining the same day/night light regime from where the lake sample was collected. All lake sample analysis was performed within 3 days of sample collection.

Light Microscopy

The contents of each lake sample were visualised in triplicate by brightfield microscopy (EVOS XL Core Imaging System, Thermo Fisher Scientific) using 10x and 40x magnification. The replicates of each lake were analysed directly from the lake water samples with the exception of lake water 6 which underwent 10-fold dilution with 0.2 μm aPES membrane (Rapid-Flow, Nalgene) filtered lake water before analysis. The brightness and contrast of the images were set to a minimum and maximum of 48 and 157, respectively and scale bars were added using ImageJ *v*1.54g[1].

Flow Cytometry

To estimate the turbidity within each lake sample, the optical density at 750 nm (OD_{750}) was measured in triplicate using a spectrophotometer (Jenway 7315) using lake water that had been filtered through a 0.2 μm aPES membrane (Rapid-Flow, Nalgene) as a blank (Table S1). For each of these samples, the number of cyanobacterial events were determined using a BD Accuri C6 flow cytometer (BD Biosciences) equipped with lasers exciting samples at 488 nm and 640 nm. The instrument was calibrated with BD CS&T Bead Lots 2309553 and 4031893 (BD Biosciences). Fluorescence data was acquired using the filters: FSC-H (488 nm excitation, 488/13), SSC-H (488 nm excitation 448/13), FL2-H (488 nm excitation, 585/40), FL3-H (488 nm excitation, 670 LP) and FL4-H (640 nm excitation, 675/25). FL3-H and FL4-H signify fluorescence from chlorophyll and phycocyanin/allophycocyanin, respectively, thus were used as the primary filters for differentiating cyanobacteria from other micro-organisms. 100 μL of lake water from each site were analysed in triplicate at a flow rate of 14 μL/min with a 10 μm core width with a threshold of 1,000 set for FSC-H. For both the OD_{750} and flow cytometry measurements, Lake water 6 underwent a 1000-fold dilution with 0.2 μm aPES membrane (Rapid-Flow, Nalgene) filtered lake water before analysis. Gating to determine and count particles corresponding to cyanobacteria was applied manually based on a FL4-H signal greater than FL3-H and at least $1x10^4$. Data was analysed using BD Accuri C6 Plus software (BD Biosciences).

Protein Extraction

To obtain cyanobacterial proteins within the lake water, 300 mL of lake water from each location was vacuum filtered using a 0.2 μm aPES membrane (Rapid-Flow, Nalgene). The material retained on the filter was resuspended in 1 mL of the corresponding filtered lake water and transferred to a microfuge tube. The tubes were then centrifuged (10,500 xg, 10 minutes, 4 °C), the supernatant was removed, and the resulting pellets were stored at -80 °C until use for protein

extraction. The cells were lysed as previously described[2]. In brief, the cells were resuspended in ultrapure water and subjected to 3 cycles of freeze-thaw followed by sonication via water-bath if necessary[2]. Successful lysis was verified by absorbance in the 620-650 nm range (Jenway 7315 spectrophotometer), indicative of phycobiliproteins released from the cyanobacterial cells. The lysed samples were centrifuged (10,500 xg, 10 minutes, 4 °C) to remove cell debris and the supernatants were buffer exchanged into 100 mM ammonium acetate pH 6.8 using Amicon Ultra 0.5 mL concentrators with a 30 kDa molecular weight cut-off filter (Merck Millipore). The solvent and pH was chosen to maintain phycobiliprotein oligomeric state[2,3]. The total protein concentration of each extract was determined by measuring the absorbance at 280 nm (Jenway 7315 spectrophotometer) and using extinction coefficient of 1 $(mg/mL)^{-1}$ cm^{-1}. For analysis by native mass spectrometry, each protein extract was diluted to 0.5 mg/mL total protein with 100 mM ammonium acetate pH 6.8. If the initial concentration of the protein extract was below this value, no dilution was carried out.

Native Mass Spectrometry

The protein extraction and native MS analysis steps take 2-3 h. Native MS was performed on the Orbitrap Eclipse Tribrid mass spectrometer using a nano-electrospray ionisation source (Thermo Fisher Scientific) fitted with gold coated borosilicate glass capillaries pulled in-house (P-1000, Sutter Instrument). Positive ion electrospray was used throughout. Full scan data was acquired in intact protein mode at high pressure with a mass range of 1000-8000 *m/z*, using a resolution setting of 7500 at 200 m/z, an automatic gain control of 100 % and a maximum injection time of 100 ms. MS^2 data was acquired on the phycobiliprotein hexamers by isolating 5000 *m/z* with a window of 1000 *m/z* and applying 20 % normalised HCD collision energy (assuming a 21+ charge state) with a mass range of 500-8000 *m/z*. For lake water 3 no hexamer peaks were observed. Instead, MS^2 data was acquired on the phycocyanin dimers by isolating the 11+ precursor at 3380 *m/z* with a window of 100 *m/z* and applying 20 % normalised HCD collision energy. For all samples, MS^3 data was acquired by isolating the most abundant 7+ phycobiliprotein monomer charge state ~ 2500 *m/z* with a window of 20 *m/z* and applying 50 % normalised HCD collision energy. All data was processed using Xcalibur *v*4.2 (Thermo Fisher Scientific).

Identification of Cyanobacteria in Lake Water

To confirm the identity of cyanobacteria present within the lake water samples, masses of phycobiliproteins observed by native MS were compared to the masses of phycobiliproteins from all known cyanobacteria strains. The α and β monomers for both allophycocyanin and phycocyanin were searched using the terms 'allophycocyanin alpha', 'allophycocyanin beta', 'phycocyanin alpha' and 'phycocyanin beta' and downloaded from UniProt (Reviewed and Unreviewed, accessed 3rd October 2024). Any entries where the protein name did not match the searched protein were excluded from the database. The masses were adjusted to account for post-translational modifications. For the α monomer of allophycocyanin, 1 x phycocyanobilin (PCB, + 586.7 Da) and 1 x N-terminal methionine loss (Met-loss, - 131.2 Da) were added, while for the α monomer of phycocyanin, only 1 x PCB (+ 586.7 Da) was added. For the β monomer of allophycocyanin, 1 x PCB (+ 586.7 Da) and methylation of asparagine (Me-Asn, + 14 Da) were added, while for the β monomer of phycocyanin 2 x

PCB (+ 1,173.4 Da) and Me-Asn (+ 14 Da) were added. For the β monomers of both allophycocyanin and phycocyanin, between 0 – 1 Met-loss modifications were added. Furthermore, where α and β monomers of allophycocyanin or phycocyanin from the same cyanobacteria strain were present in the database, the masses were combined to create a database of known phycobiliprotein dimers. The phycobiliprotein dimers and monomers of the lake protein extracts observed via MS^1 and MS^2, respectively, were searched against the filtered and mass adjusted UniProt database. Matches were determined with a tolerance of +/- 2 Da on the observed masses. To reduce false positive identifications, the cyanobacteria identified were sorted according to their habitat (i.e. salt or freshwater environment), and any cyanobacteria known to survive only in salt-water removed from the identification list.

Bottom-up proteomics LC-MS analysis

Protein extracts from all lake samples except lake 3, due to low protein abundance, were digested with trypsin (Promega) in 100 mM ammonium bicarbonate pH 8.0 for 16 hours at 37 °C at a 1:50 ratio of enzyme:protein. Digestion was quenched by the addition of formic acid (10 % *v/v* final concentration). Liquid chromatography-mass spectrometry was performed using a Dionex UltiMate 3000 HPLC system (Thermo Fisher Scientific) coupled to a Q-Exactive HF mass spectrometer (Thermo Fisher Scientific). Samples were kept at 4 °C and the injection volume was set to 5 μL corresponding to approximately 2.5 μg of protein. Peptides were desalted on a PepMap100 C18 nanoViper trap column (75 μM x 2 cm, 3 μM particle size) (Thermo Fisher Scientific) before being separated using a PepMap100 C18 nanoViper analytical column (75 μM x 15 cm, 3 μM particle size) (Thermo Fisher Scientific) with a gradient elution from 3.2 % A (100 % water and 0.1 % formic acid) to 44 % B (100 % acetonitrile and 0.1 % formic acid) over 30 minutes. The column was kept at a temperature of 40 °C and a flow rate of 350 nL/min. Full scan data was acquired in positive mode with a mass range between 380-1600 *m/z*, resolution of 120,000 at 200 *m/z*, automatic gain control set to 3×10^6 and a maximum injection time of 50 ms. Precursors for MS^2 data acquisition were selected based on the Top20 most abundant ions. Isolation and fragmentation occurred in the quadrupole with a 1.2 *m/z* window and a normalised HCD energy of 28 %. Only precursor charge states between 2+ and 6+ were selected for fragmentation and dynamic exclusion was employed for 20 s on a single charge state per precursor. For the tandem mass spectrometry analyses, the resolution was set to 15,000, the maximum injection time was 50 ms and the AGC was set to 1×10^5.

All RAW files were processed and analysed using Proteome Discoverer *v*.2.5. (Thermo Fisher Scientific) and database searching was performed with Sequest HT[4] The default settings were used throughout unless otherwise stated. Trypsin cleavage was set to full and the maximum number of missed cleavages set to 3. The protein database was obtained from UniProt by searching 'allophycocyanin alpha', 'allophycocyanin beta', 'phycocyanin alpha' and 'phycocyanin beta' (Reviewed and Unreviewed, accessed 11th June 2025). A precursor mass tolerance of 10 ppm was used with a fragment mass tolerance of 0.02 Da. Dynamic modifications included oxidation (+ 15.995 Da, M), methylation (+ 14.016 Da, N), N-terminal methionine loss (- 131.040 Da, M), the addition of phycocyanobilin (+ 586.279 Da, C) and the addition of phycoerythrobilin (+ 588.295 Da, C) with the maximum number of modifications per peptide set to 3. The peptide validator node was set to filter for a false discovery rate of 0.01. Reported proteins were filtered to contain ≥ 2 peptides and

PSMs that were found in one replicate. This stringent criteria aids with correct species identification, however, due to the high sequence similarity of certain proteins between species, false positive identification via bottom-up proteomics occur and further validation is required. To reduce false positive identifications, the cyanobacteria identified were sorted according to their habitat (i.e. salt or freshwater environment), and any cyanobacteria known to survive only in salt-water removed from the identification list. Identified species corresponding to red algae were not included within the proteomics identification list.

Cyanotoxin extraction

To extract cyanotoxins from the lake water samples, solid phase extraction was performed using 500 mg, 6 mL Supelclean™ ENVI™-18 cartridges (Sigma-Aldrich). In brief, each cartridge was conditioned with 100 % MeOH followed by LC-MS grade water before being loaded with 200 mL of vacuum filtered lake water at a flow rate of approximately 2 mL/min. Polar contaminants were removed from the cartridge bed with LC-MS grade water and 20 % MeOH, then retained cyanotoxins were eluted step-wise with 900 µL 80 % MeOH followed by 100 µL 100 % MeOH before storage of the cyanotoxin extracts at -80 °C. Extracts were dried by vacuum evaporation (1,400 rpm, 45 °C), resuspended in 50 % MeOH and then diluted 10-fold in LC-MS grade water before analysis by liquid chromatography-mass spectrometry.

Cyanotoxin LC-MS analysis

Lyophilised MC-RR, -YR, -LR, -LA, -LW, -LF and NOD-R were purchased from Enzo Life Sciences and resuspended in 50-100 % methanol and stored at -20 °C until use. A stock solution containing each cyanotoxin standard at 200 µg/L was made with 5% methanol. Dilutions of the stock solution from 200 to 0.1 µg/L were prepared to form calibration curve points. Liquid chromatography-mass spectrometry was performed using a Dionex UltiMate 3000 UHPLC system (Thermo Fisher Scientific) coupled to a Q-Exactive mass spectrometer equipped with a heated electrospray ionisation source (Thermo Fisher Scientific). A Hypsersil GOLD™ C18 column (100 mm x 2.1 mm i.d., 1.9 µm) (Thermo Fisher Scientific) was used with the column temperature set to 40 °C and a flow rate of 0.350 mL/min. Samples were kept at 4 °C with the injection volume set to 10 µL. Cyanotoxins were separated using mobile phases A (100 % water, 0.1 % formic acid) and B (100 % acetonitrile, 0.1 % formic acid) with a gradient of 5 % to 35 % B over 2 minutes, 35 % to 90 % B over 7.5 minutes and a hold at 90 % B for 2 minutes before re-equilibration at 5 % B for 3.5 minutes. The mass spectrometer was set to an electrospray voltage of 3.6 kV, capillary temperature of 320 °C, sheath gas of 7, auxiliary gas of 3, spare gas of 1, and a heater temperature of 100 °C. Full scan data was acquired in positive mode with a mass range between 400-1600 *m/z*, resolution of 35,000, automatic gain control set to 3×10^6 and a maximum injection time of 100 ms. Precursors for MS^2 data acquisition were isolated using an inclusion list (Table S6) and a method whereby only the Top7 most abundant ions were selected. Isolation and fragmentation occurred in the quadrupole with a 5 *m/z* window and a normalised HCD energy of 35 %. Only precursor charge states between 1+ and 2+ were selected for fragmentation, dynamic exclusion was turned off and the threshold for fragmentation was 1×10^3. For the tandem mass spectrometry analyses, the AGC was set to 1×10^2.

The raw data was analysed using the Processing Setup and Quan Browser software from Xcalibur *v*4.2 (Thermo Fisher Scientific). For each cyanotoxin, a processing method was created containing the expected *m/z*, retention time and retention window (20 seconds). Peak detection was performed using Genesis set to highest peak mode and a minimum signal to noise of 3, while peak integration was performed with smoothing set to 15 and a signal to noise threshold of 0.5. The resolution threshold was set to 50 %. Each detected and integrated peak was manually confirmed. Calibration curves were generated in linear mode using equal weighting and ignoring the origin. The LOD and LOQ of each cyanotoxin was calculated using the standard error and the slope of the calibration curve (n=3). The determined LOD and LOQ for each toxin standard is stated within Figure S24.

References

(1) Schneider, C. A.; Rasband, W. S.; Eliceiri, K. W. NIH Image to ImageJ: 25 Years of Image Analysis. *Nat. Methods* **2012**, *9* (7), 671–675. https://doi.org/10.1038/nmeth.2089.

(2) Sound, J. K.; Peters, A.; Bellamy-Carter, J.; Rad-Menéndez, C.; MacKechnie, K.; Green, D. H.; Leney, A. C. Rapid Cyanobacteria Species Identification with High Sensitivity Using Native Mass Spectrometry. *Anal. Chem.* **2021**, *93* (42), 14293–14299. https://doi.org/10.1021/acs.analchem.1c03412.

(3) Leney, A. C.; Tschanz, A.; Heck, A. J. R. Connecting Color with Assembly in the Fluorescent B-Phycoerythrin Protein Complex. *FEBS J.* **2018**, *285* (1), 178–187. https://doi.org/10.1111/febs.14331.

(4) Tabb, D. L. The SEQUEST Family Tree. *J. Am. Soc. Mass Spectrom.* **2015**, *26* (11), 1814–1819. https://doi.org/10.1007/s13361-015-1201-3.

SUPPLEMENTARY FIGURES

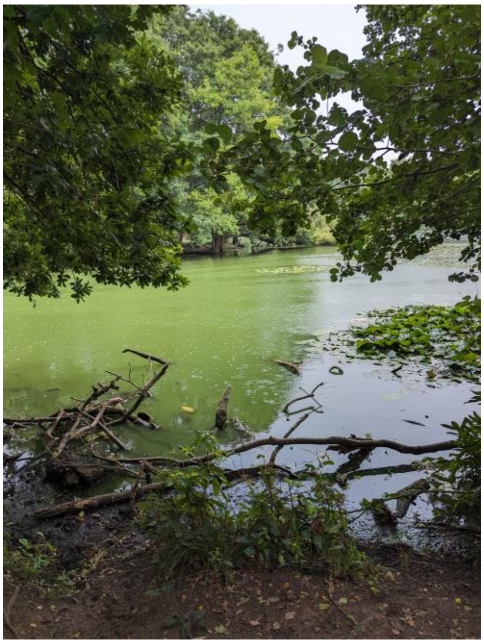

Figure S1: Photograph of cyanobacterial bloom at time of collection of lake water 1.

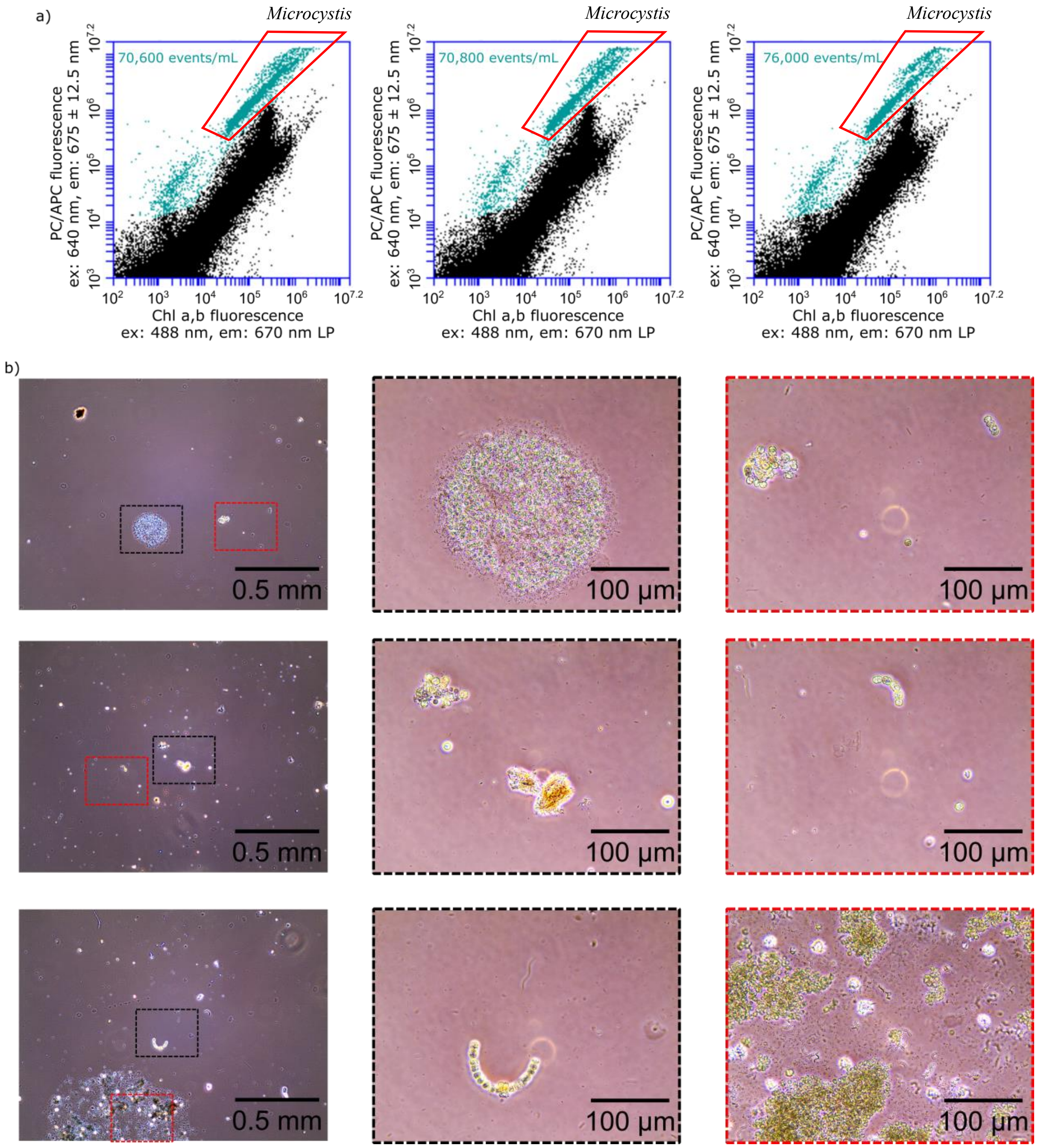

Figure S2: (a) Flow cytometry of lake water 1 shows the presence of cyanobacteria (teal). A separate cytogram is shown for each measurement (three separate samples taken from the lake water 1). The population likely corresponding to *Microcystis* is shown in the red box. (b) Light microscopy of lake water 1 shows cyanobacterial features. Three example images at 40x magnification are shown with regions selected for visualisation at 10x magnification (black and red boxes).

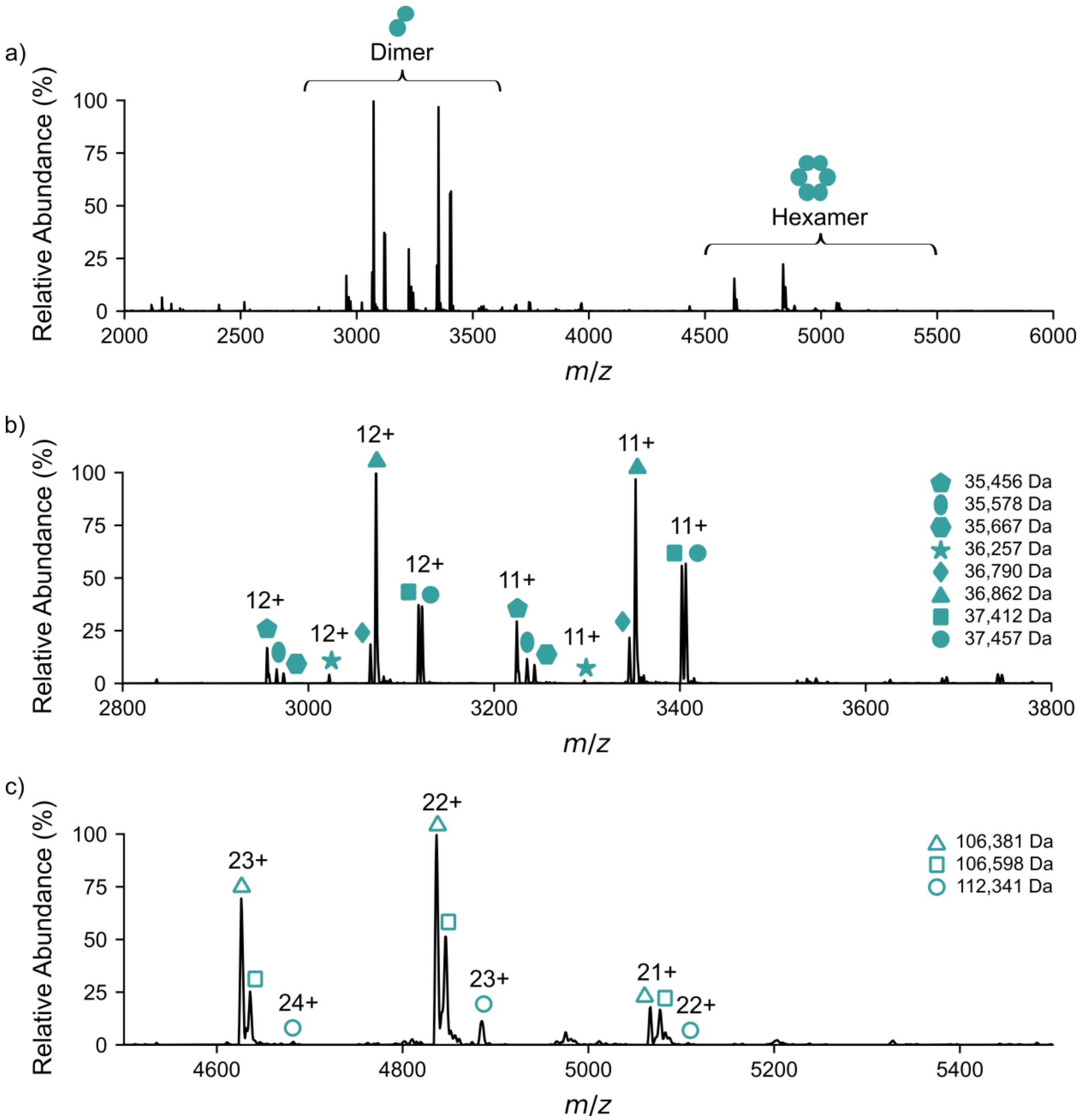

Figure S3: Native MS of the lysate from lake water 1 (a) showed charge state distributions corresponding to the dimeric (b) and hexameric (c) forms of phycobiliproteins.

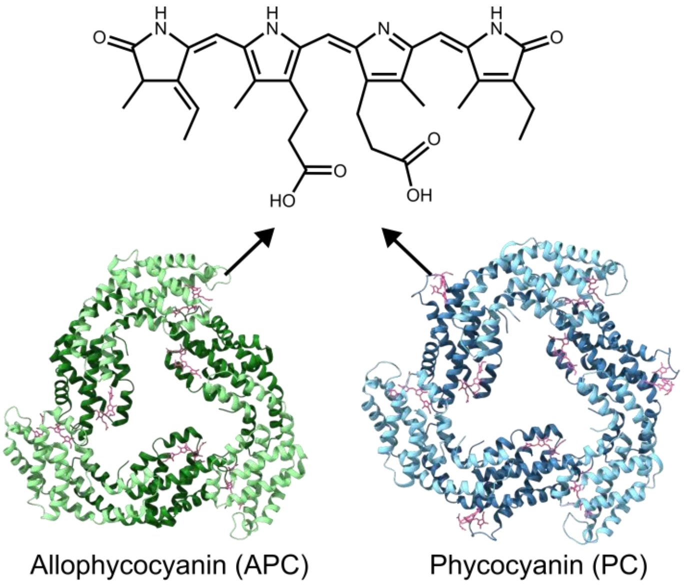

Figure S4: Hexameric structures of main phycobiliprotein components within the phycobilisomes in most cyanobacteria. Allophycocyanin (APC) and phycocyanin (PC) are coloured green and blue, respectively. Light and dark colours represent the alpha and beta subunits, respectively. The phycobiliprotein chromophore, phycocyanobilin is shown in pink.

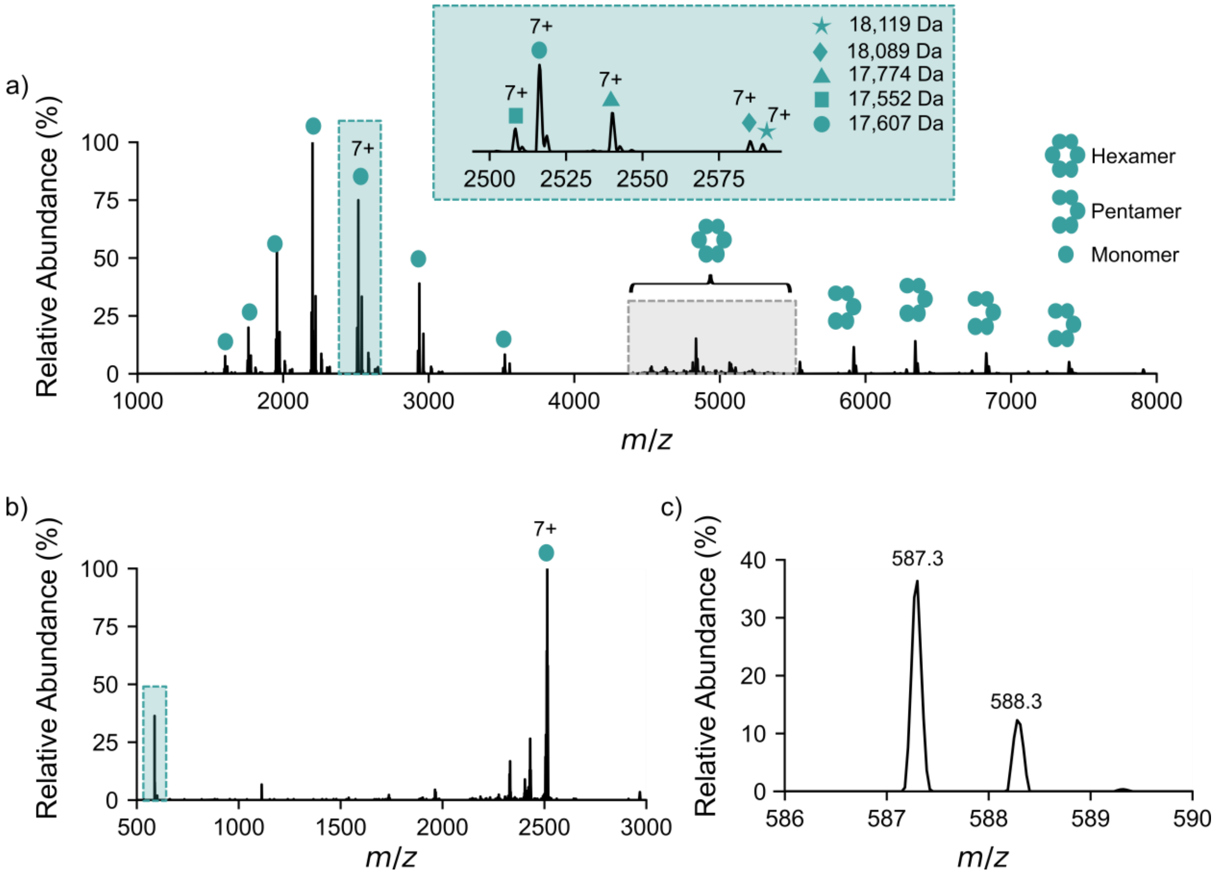

Figure S5: The hexameric region of the lysate from lake water 1 (grey, 5000 ± 1000 *m/z*) was selected for MS^2 using 20 % HCD (a) to produce charge state distributions corresponding to the monomeric and pentameric forms of phycobiliproteins. The monomeric 7+ charge state (blue) was selected and MS^3 performed using 50 % HCD (b) to release the phycocyanobilin chromophore (c).

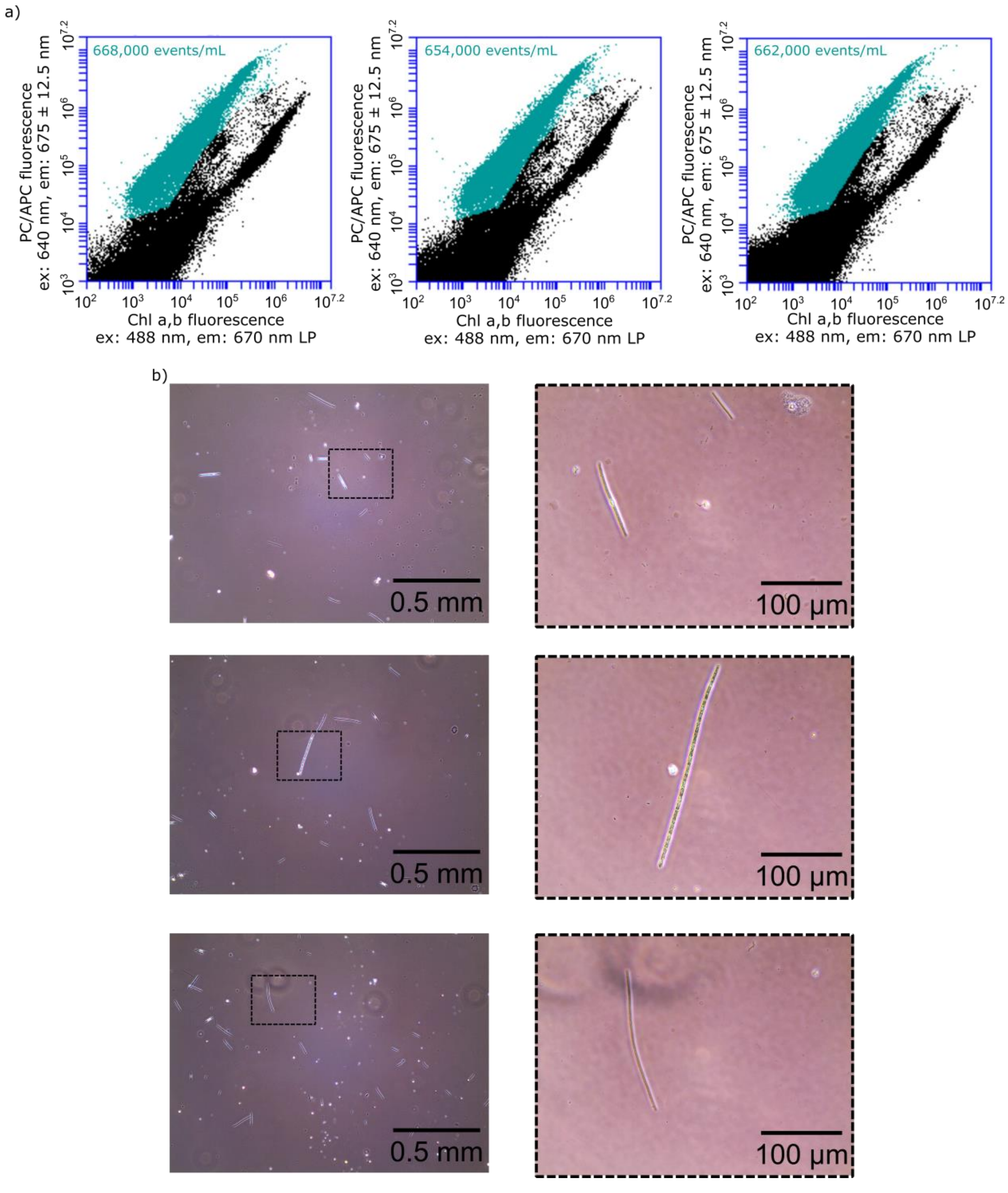

Figure S6: (a) Flow cytometry of lake water 2 shows the presence of cyanobacteria (teal). A separate cytogram is shown for each measurement (three separate samples taken from the lake water 2). (b) Light microscopy of lake water 2 shows cyanobacterial features. Three example images at 40x magnification are shown with regions selected for visualisation at 10x magnification (black boxes).

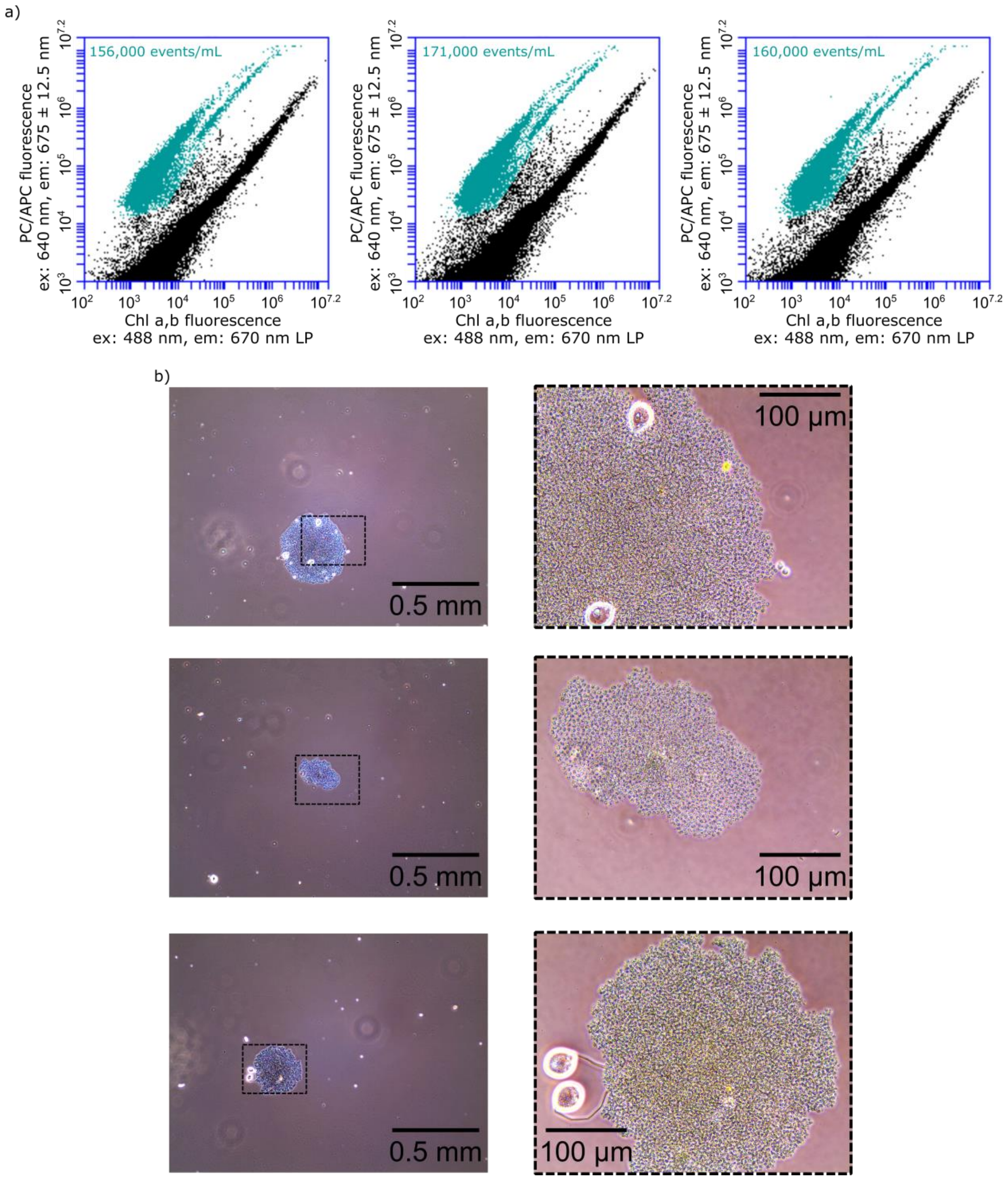

Figure S7: Flow cytometry of lake water 3 shows the presence of cyanobacteria (teal). A separate cytogram is shown for each measurement (three separate samples taken from the lake water 3). (b) Light microscopy of lake water 3 shows cyanobacterial features. Three example images at 40x magnification are shown with regions selected for visualisation at 10x magnification (black boxes).

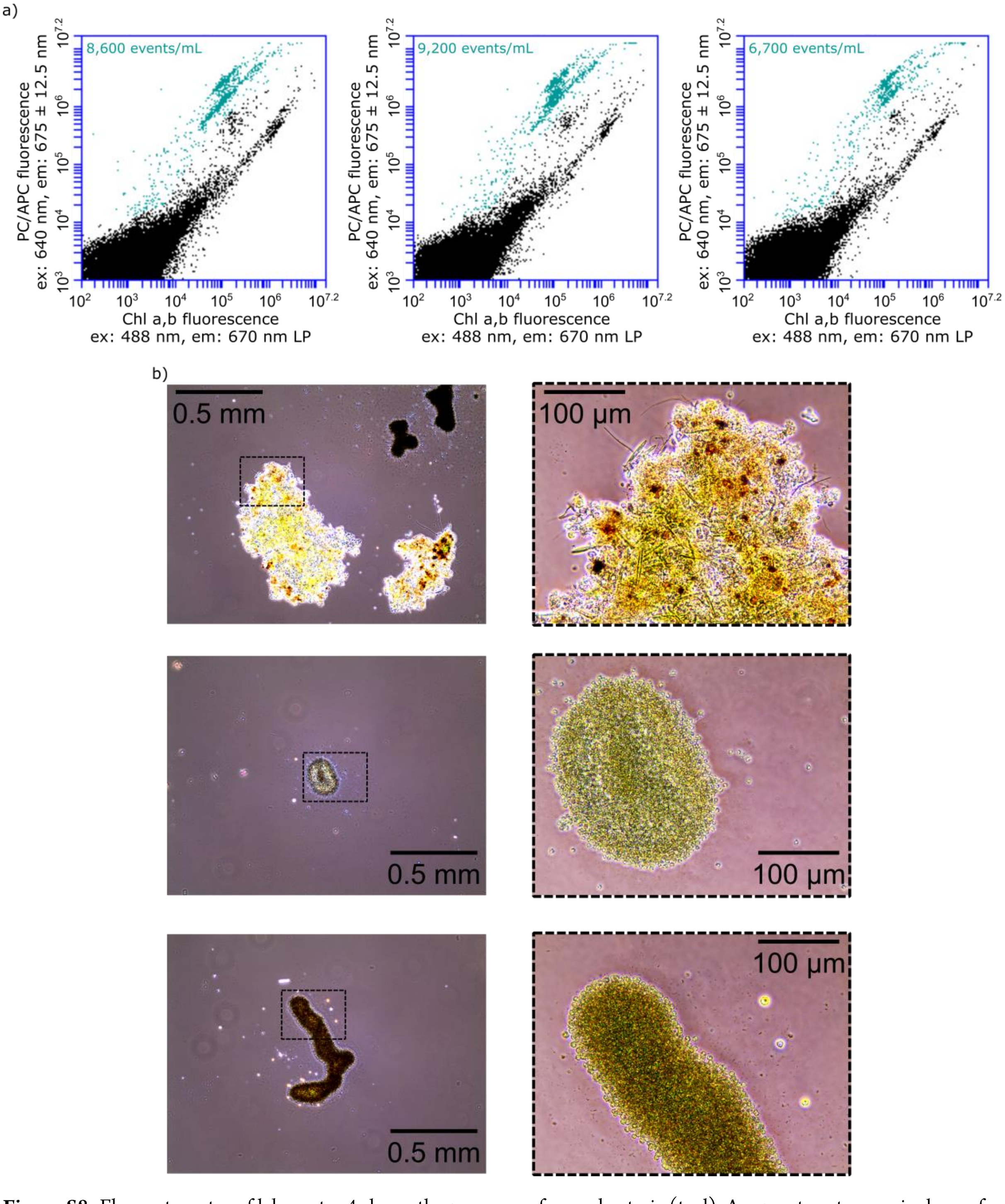

Figure S8: Flow cytometry of lake water 4 shows the presence of cyanobacteria (teal). A separate cytogram is shown for each measurement (three separate samples taken from the lake water 1). (b) Light microscopy of lake water 4 shows cyanobacterial features. Three example images at 40x magnification are shown with regions selected for visualisation at 10x magnification (black boxes).

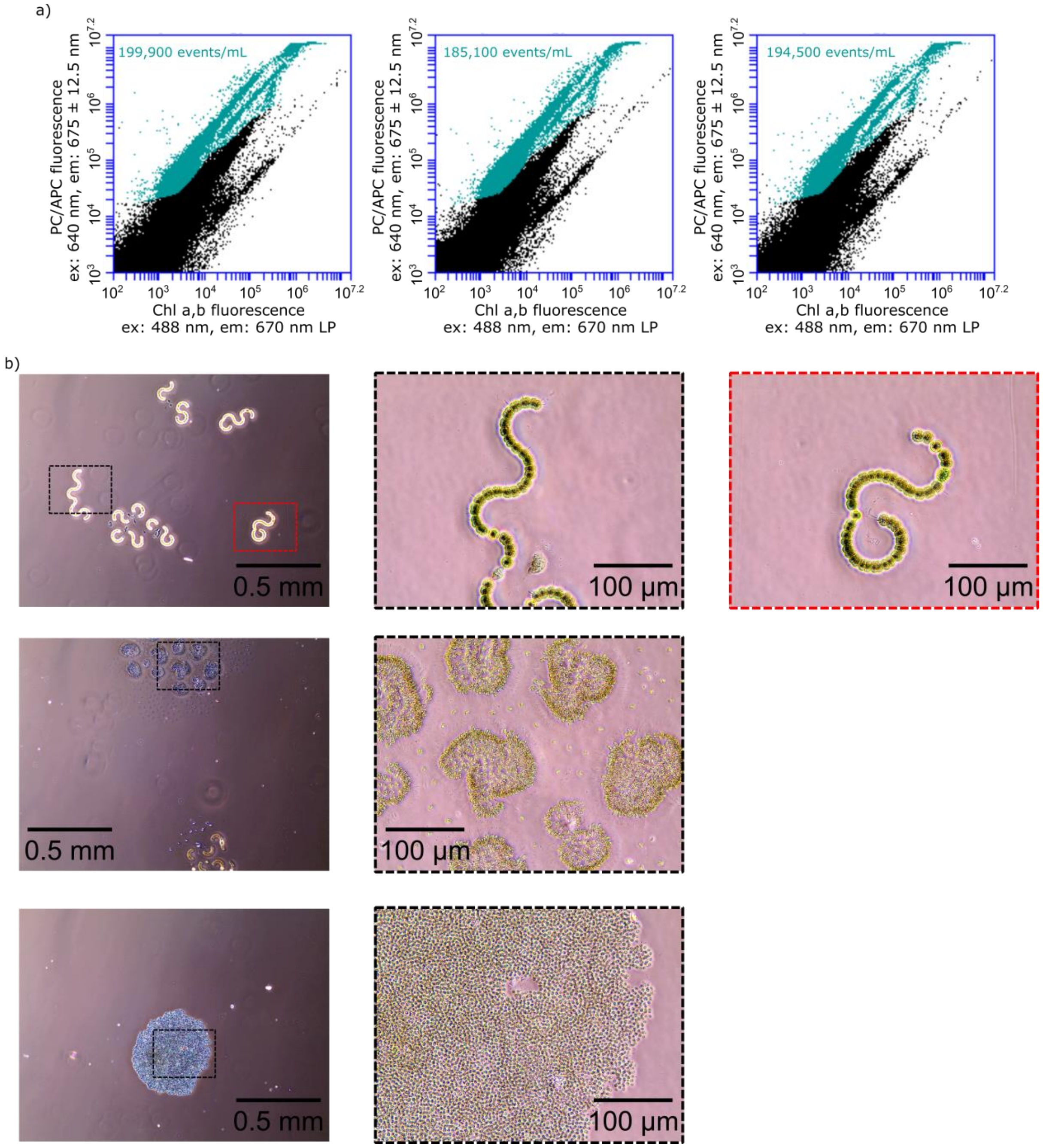

Figure S9: Flow cytometry of lake water 5 shows the presence of cyanobacteria (teal). A separate cytogram is shown for each measurement (three separate samples taken from the lake water 5). (b) Light microscopy of lake water 5 shows cyanobacterial features. Three example images at 40x magnification are shown with regions selected for visualisation at 10x magnification (black and red boxes).

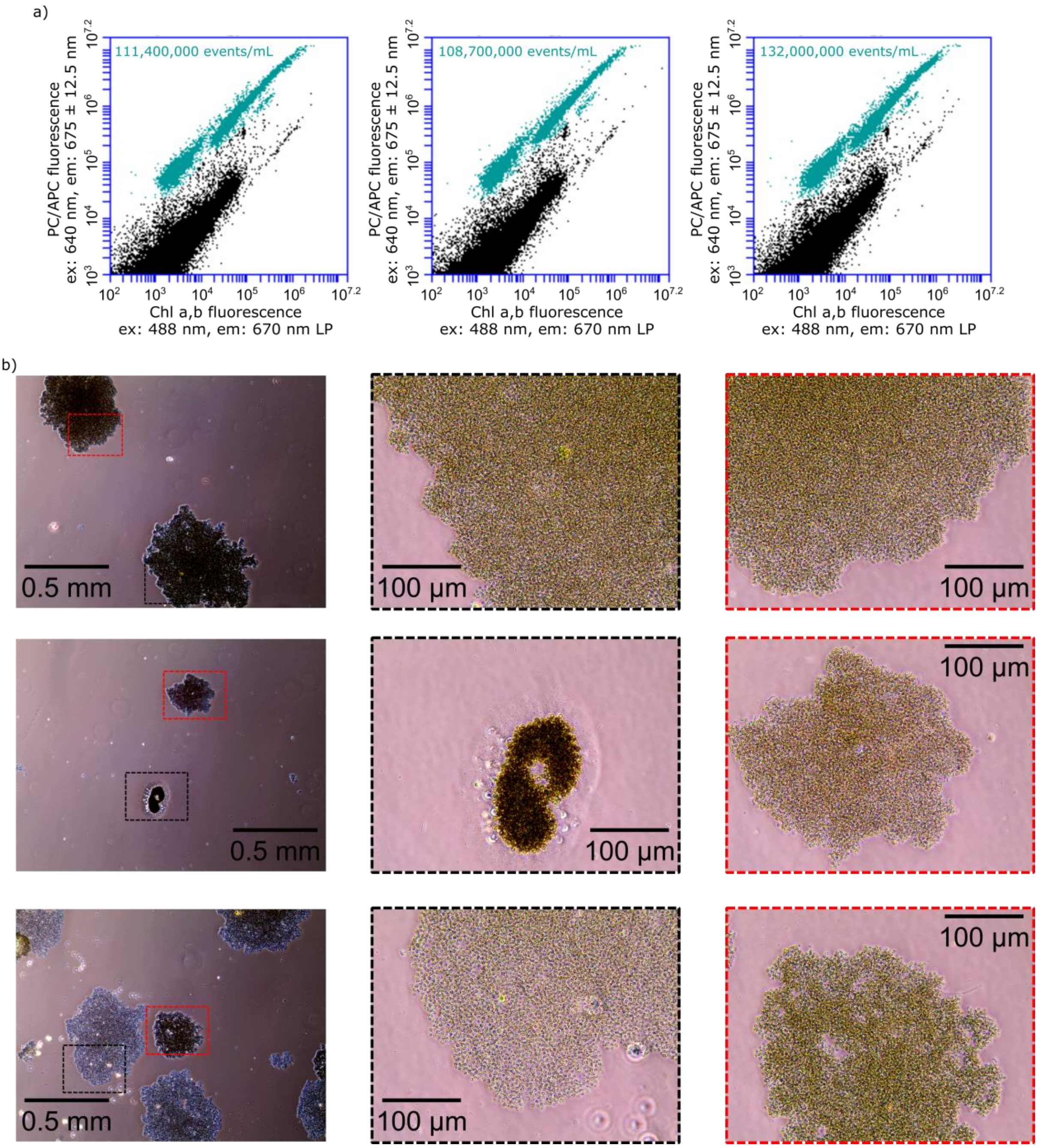

Figure S10: Flow cytometry of lake water 6 shows the presence of cyanobacteria (teal). A separate cytogram is shown for each measurement (three separate samples taken from the lake water 6). (b) Light microscopy of lake water 6 shows cyanobacterial features. Three example images at 40x magnification are shown with regions selected for visualisation at 10x magnification (black and red boxes). Note, due to the high abundance of cellular material, lake water 6 was 1000-fold diluted prior to flow cytometry and 10-fold diluted prior to light microscopy measurements.

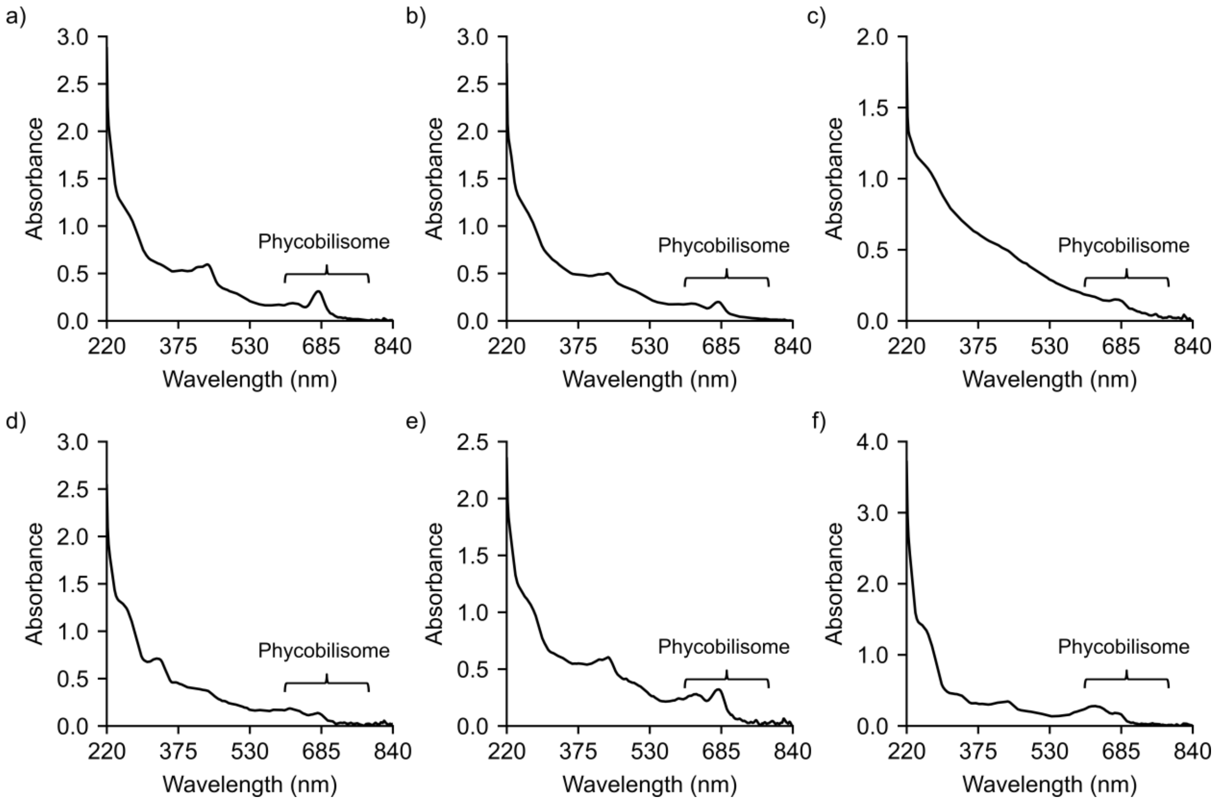

Figure S11: Absorbance spectra of lysed material from lake water 1 (a), lake water 2 (b), lake water 3 (c), lake water 4 (d), lake water 5 (e) and lake water 6 (f). The region where phycobilisome proteins are expected to absorb is labelled.

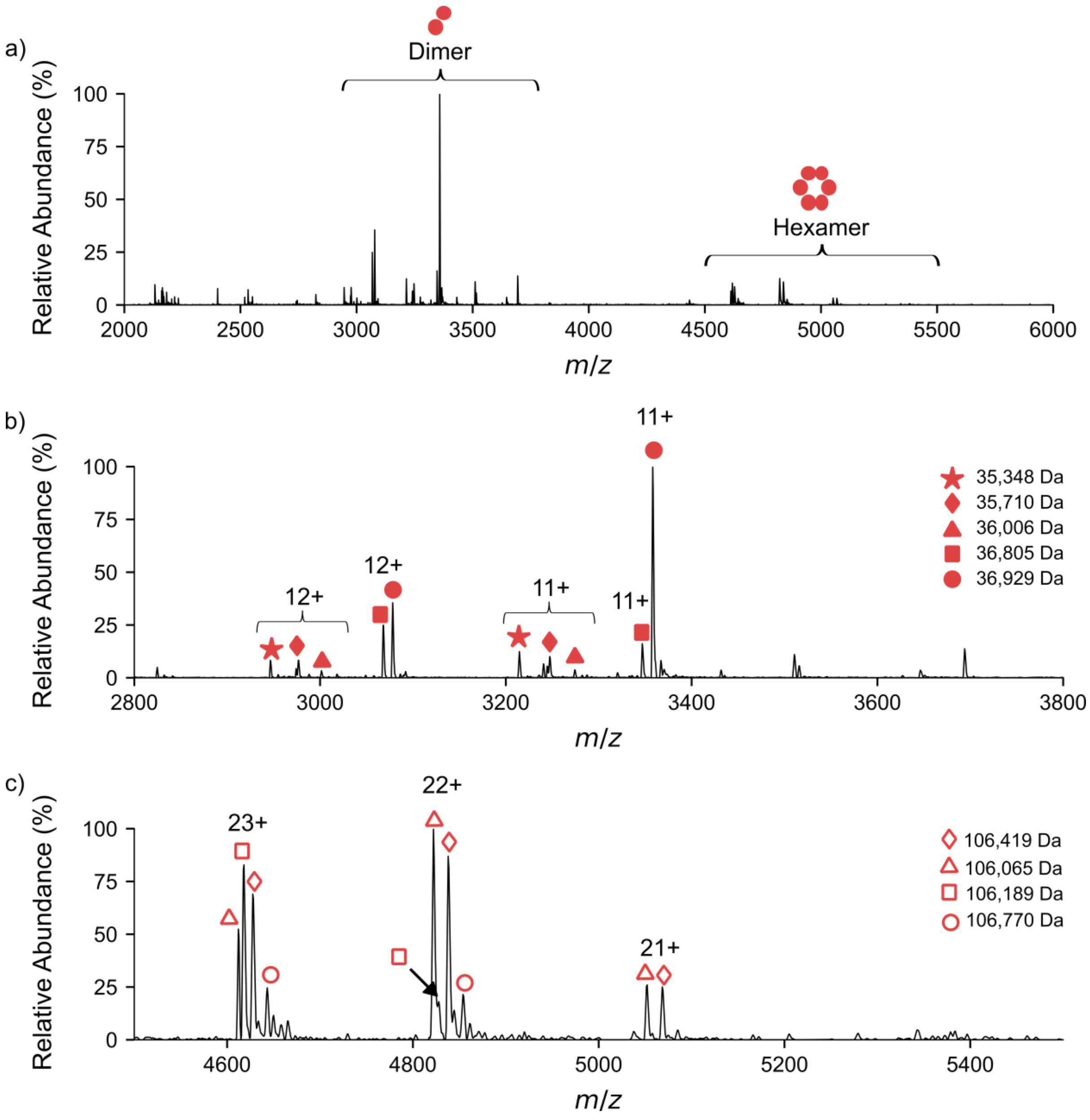

Figure S12: Native MS of the lysate from lake water 2 (a) showed charge state distributions corresponding to the dimeric (b) and hexameric (c) forms of phycobiliproteins.

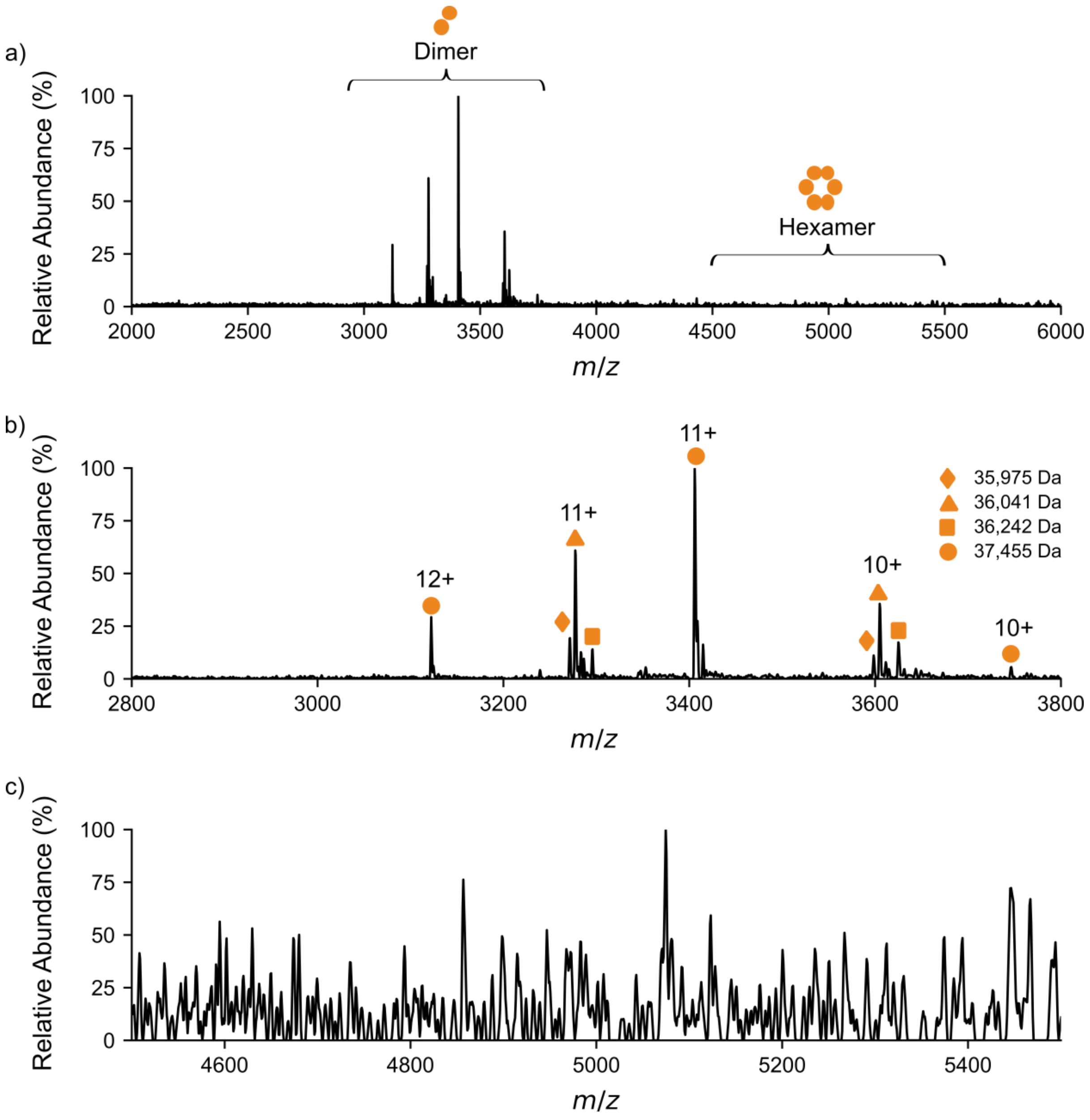

Figure S13: Native MS of the lysate from lake water 3 (a) showed charge state distributions corresponding to the dimeric (b) forms of phycobiliproteins. Note, due to low sample abundance, no obvious charge state distributions were present corresponding to hexameric complexes.

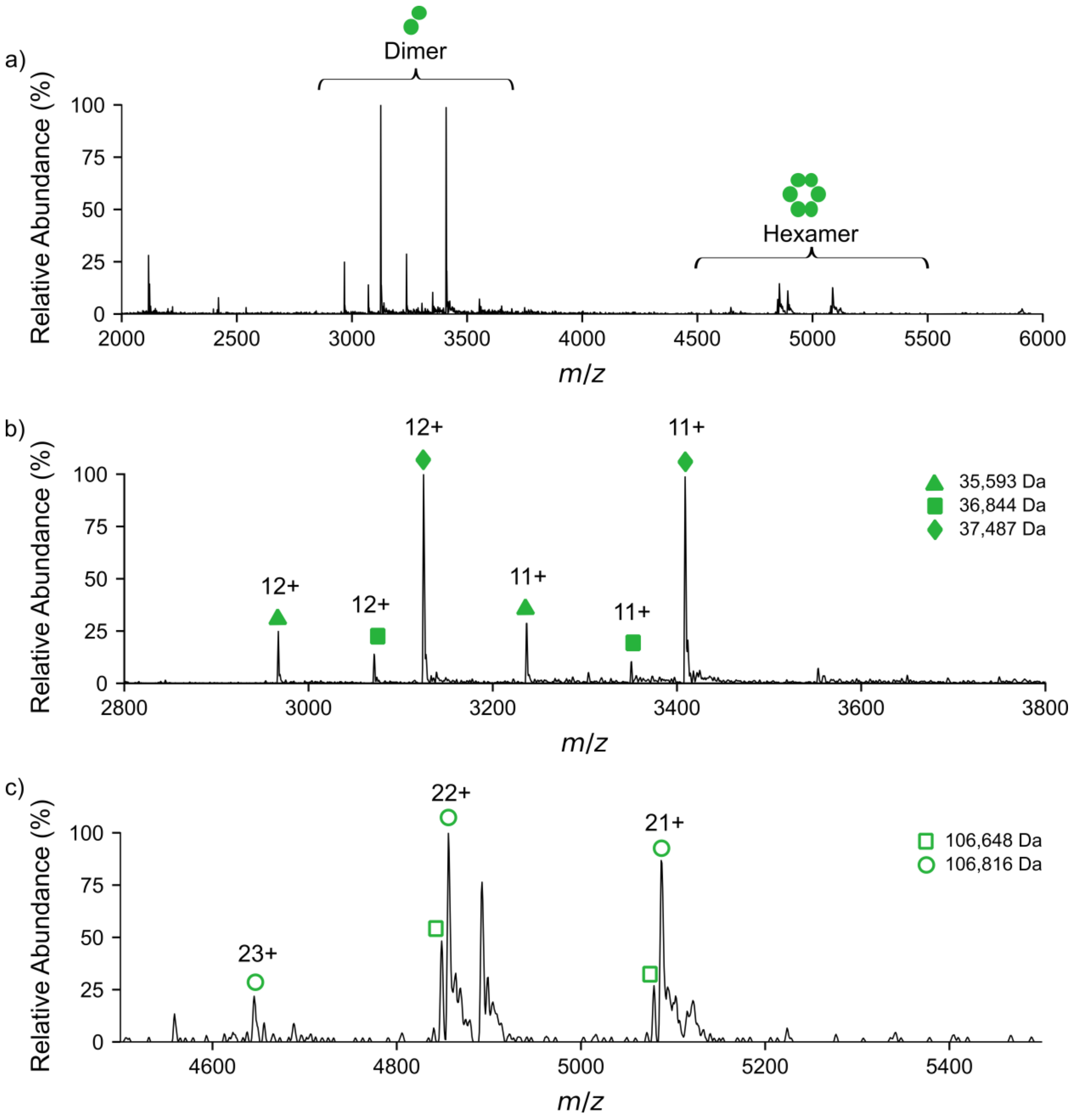

Figure S14: Native MS of the lysate from lake water 4 (a) showed charge state distributions corresponding to the dimeric (b) and hexameric (c) forms of phycobiliproteins.

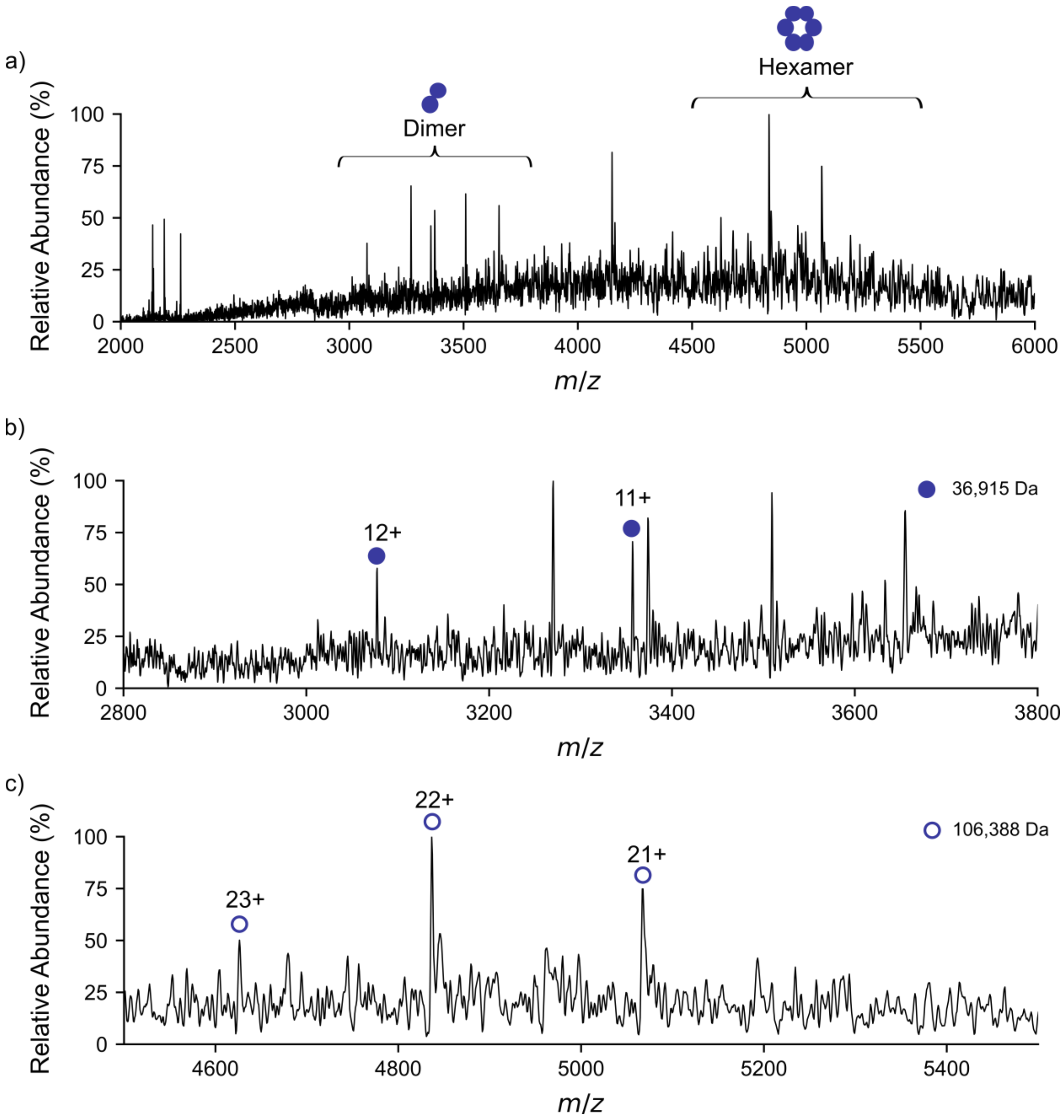

Figure S15: Native MS of the lysate from lake water 5 (a) showed charge state distributions corresponding to the dimeric (b) and hexameric (c) forms of phycobiliproteins.

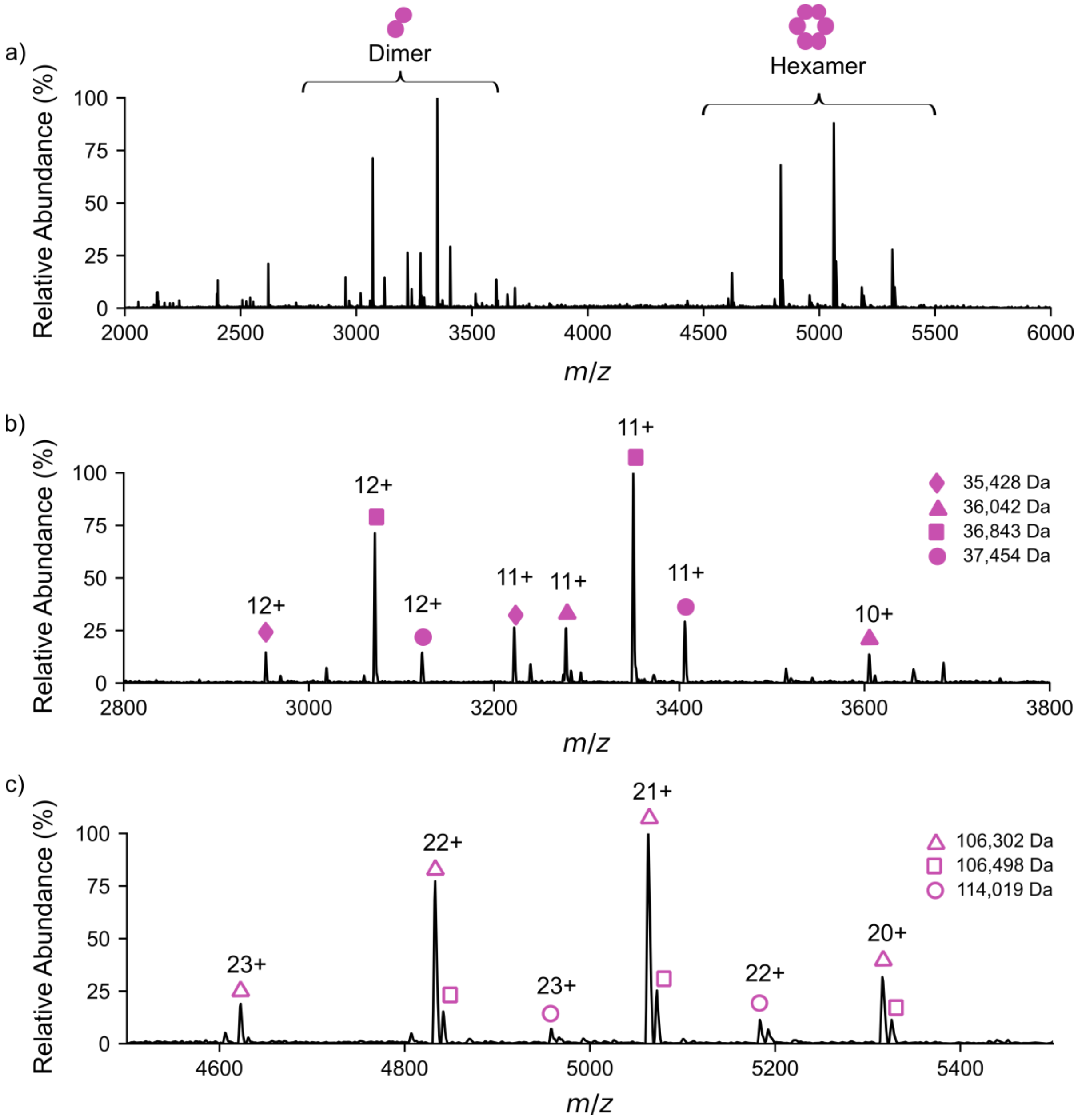

Figure S16: Native MS of the lysate from lake water 6 (a) showed charge state distributions corresponding to the dimeric (b) and hexameric (c) forms of phycobiliproteins.

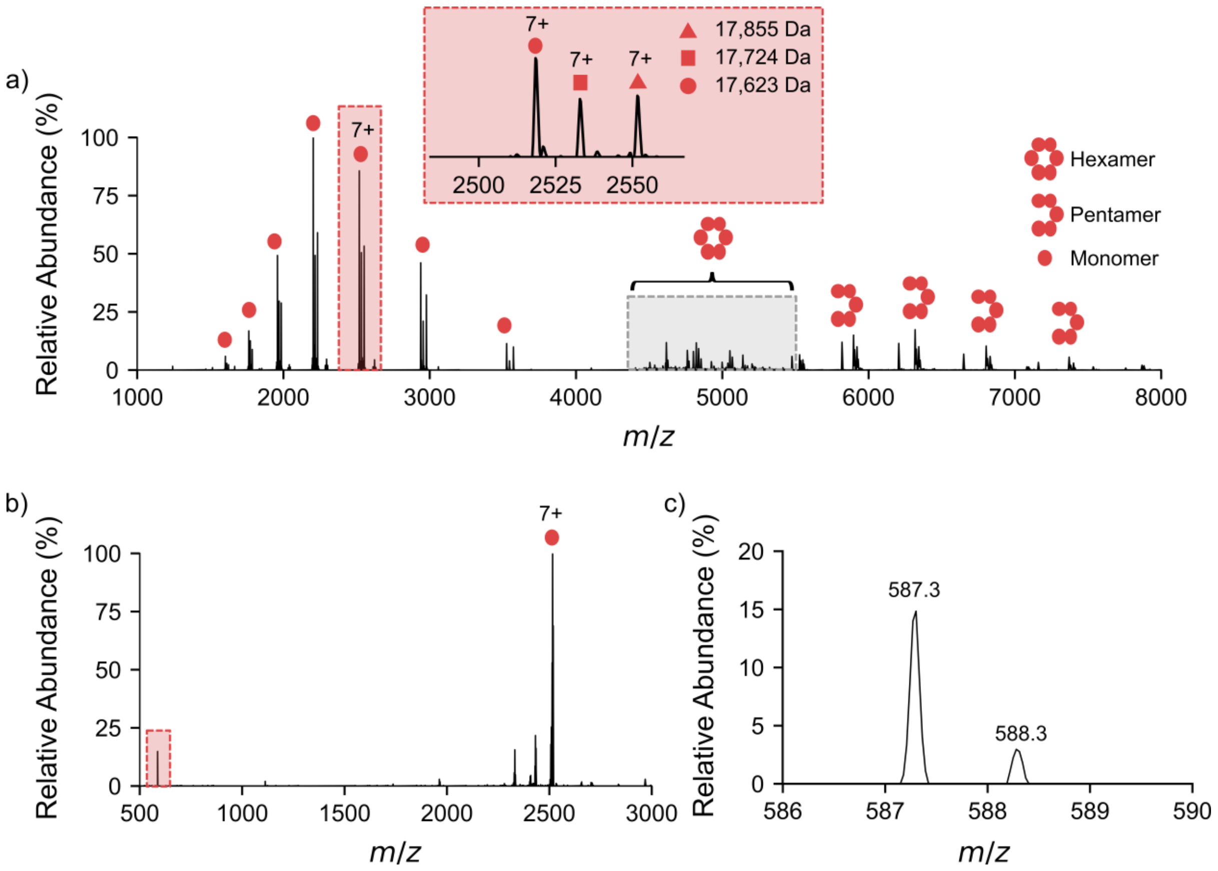

Figure S17: The hexameric region of the lysate from lake water 2 (grey, 5000 ± 1000 *m/z*) was selected for MS^2 using 20 % HCD (a) to produce charge state distributions corresponding to the monomeric and pentameric forms of phycobiliproteins. The monomeric 7+ charge state (red) was selected and MS^3 performed using 50 % HCD (b) to release the phycocyanobilin chromophore (c).

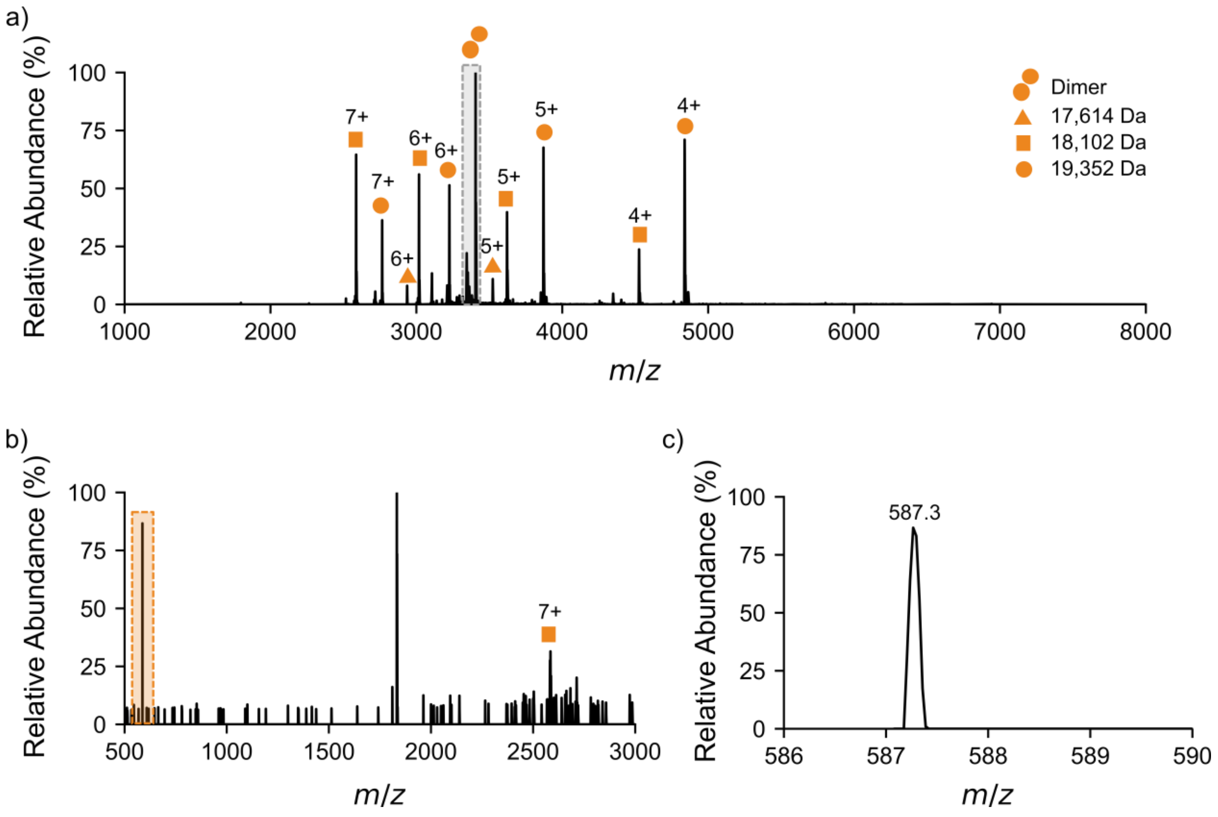

Figure S18: The PC dimer region of the lysate from lake water 3 (grey, 3380 ± 100 *m/z*) was selected for MS^2 using 20 % HCD (a) to produce charge state distributions corresponding to the monomeric forms of phycobiliproteins. The monomeric 7+ charge state was selected for MS^3 using 50 % HCD (b) to release the phycocyanobilin chromophore (c).

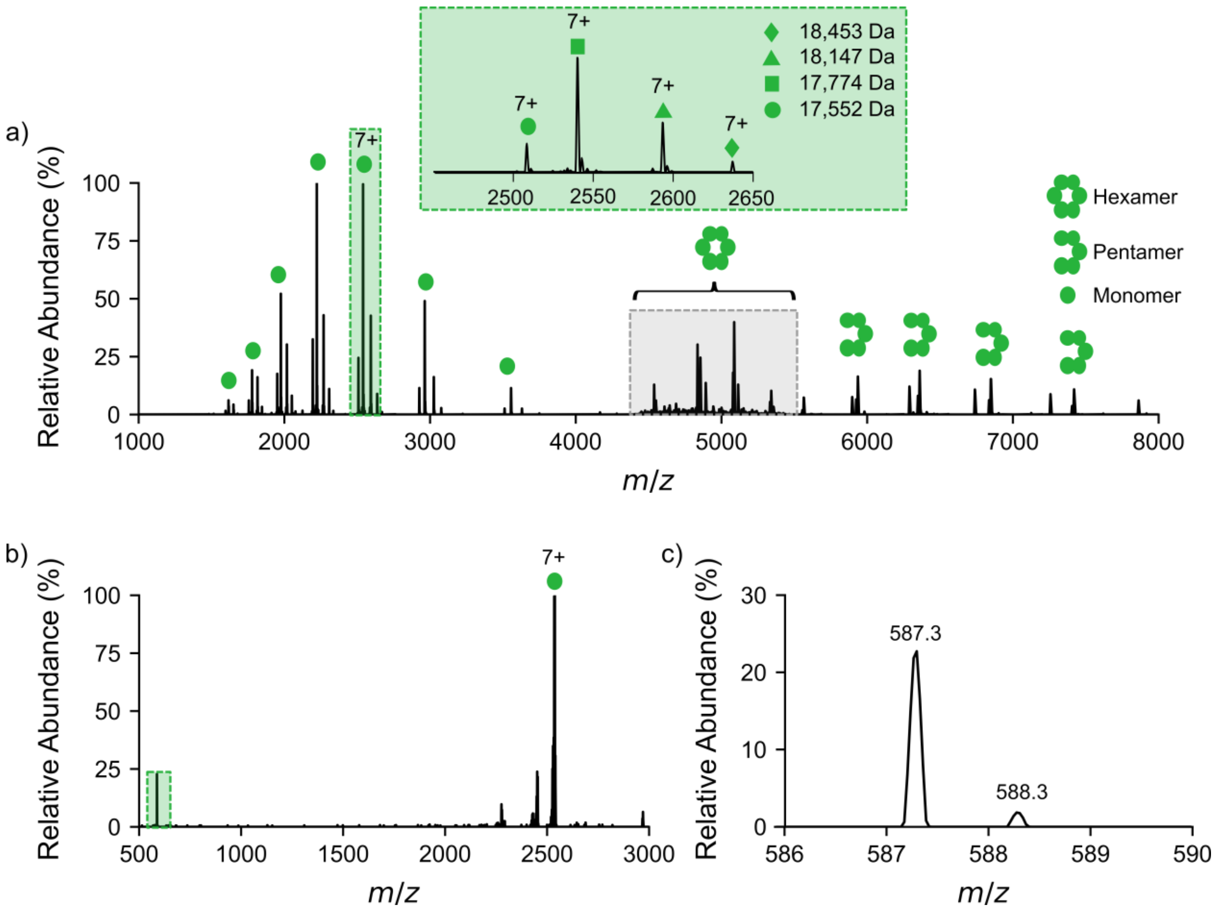

Figure S19: The hexameric region of the lysate from lake water 4 (grey, 5000 ± 1000 *m/z*) was selected for MS^2 using 20 % HCD (a) to produce charge state distributions corresponding to the monomeric and pentameric forms of phycobiliproteins. The monomeric 7+ charge state (green) was selected and MS^3 performed using 50 % HCD (b) to release the phycocyanobilin chromophore (c).

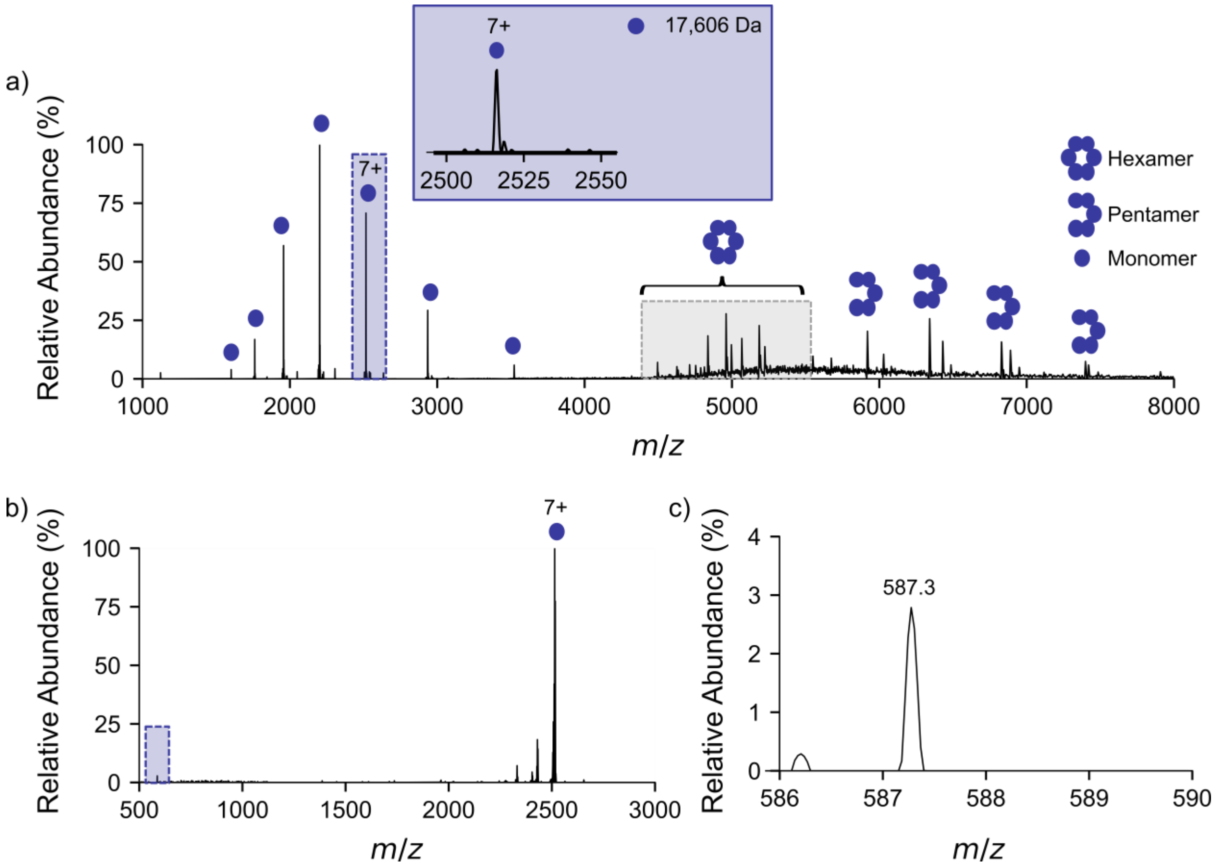

Figure S20: The hexameric region of the lysate from lake water 5 (grey, 5000 ± 1000 *m/z*) was selected for MS^2 using 20 % HCD (a) to produce charge state distributions corresponding to the monomeric and pentameric forms of phycobiliproteins. The monomeric 7+ charge state (blue) was selected and MS^3 performed using 50 % HCD (b) to release the phycocyanobilin chromophore (c).

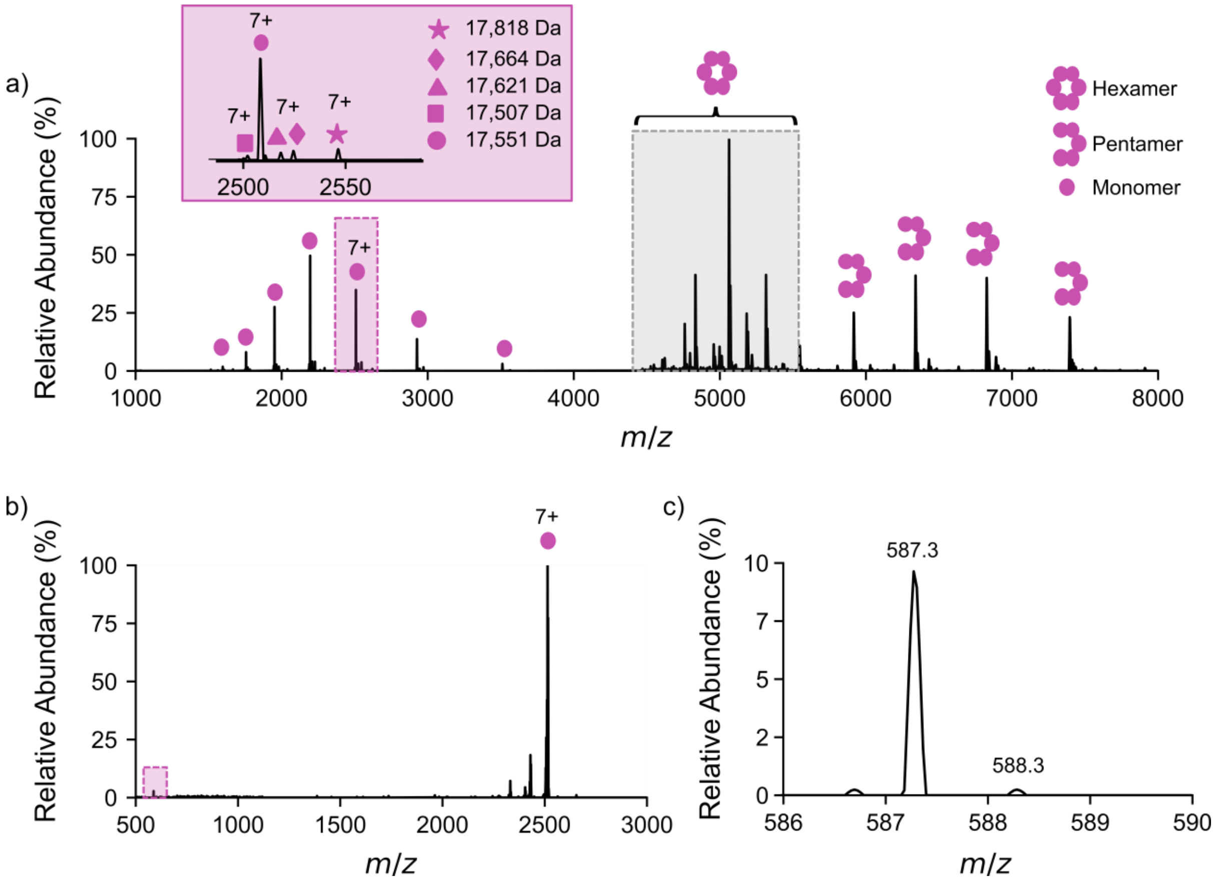

Figure S21: The hexameric region of the lysate from lake water 6 (grey, 5000 ± 1000 *m/z*) was selected for MS^2 using 20 % HCD (a) to produce charge state distributions corresponding to the monomeric and pentameric forms of phycobiliproteins. The monomeric 7+ charge state (pink) was selected and MS^3 performed using 50 % HCD (b) to release the phycocyanobilin chromophore (c).

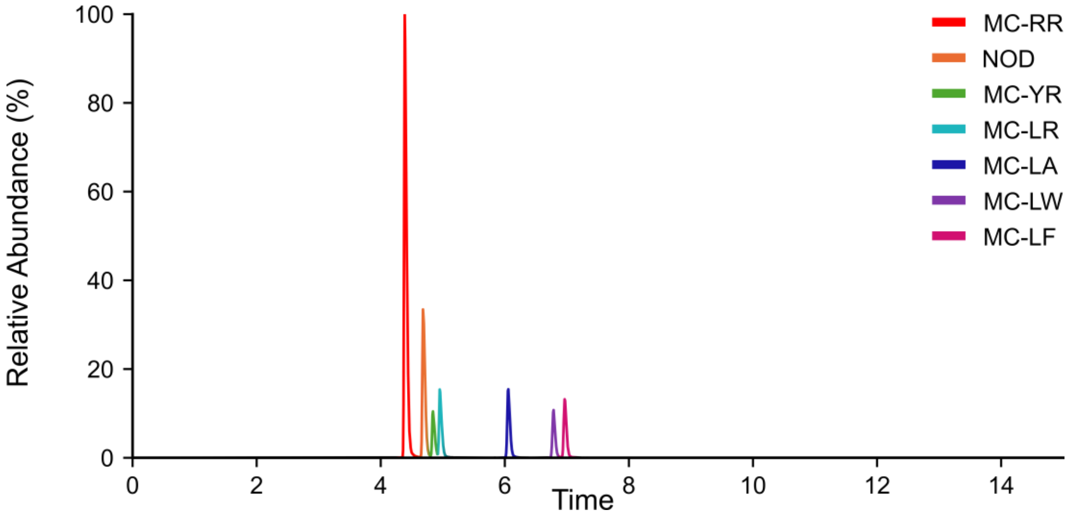

Figure S22: Liquid chromatography of cyanotoxins MC-RR, NOD, MC-YR, MC-LR, MC-LA, MC-LW and MC-LF at 50 µg/L.

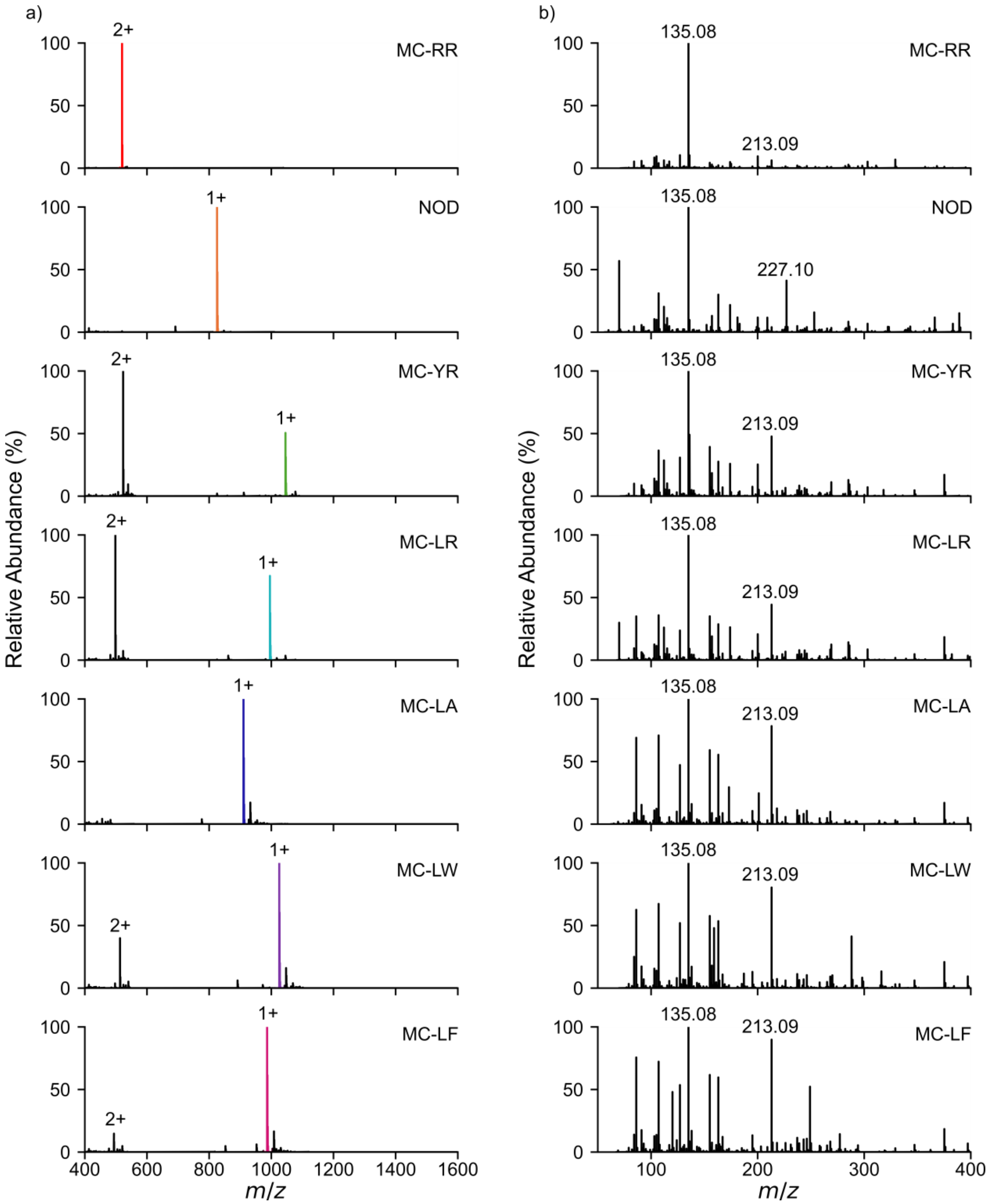

Figure S23: Mass spectra of cyanotoxins MC-RR (red), NOD (orange), MC-YR (green), MC-LR (blue), MC-LA (dark blue), MC-LW (purple) and MC-LF (pink) at 50 µg/L (a). The coloured ions were selected for MS/MS (b) which produced cyanotoxin specific peptide fragments (labelled).

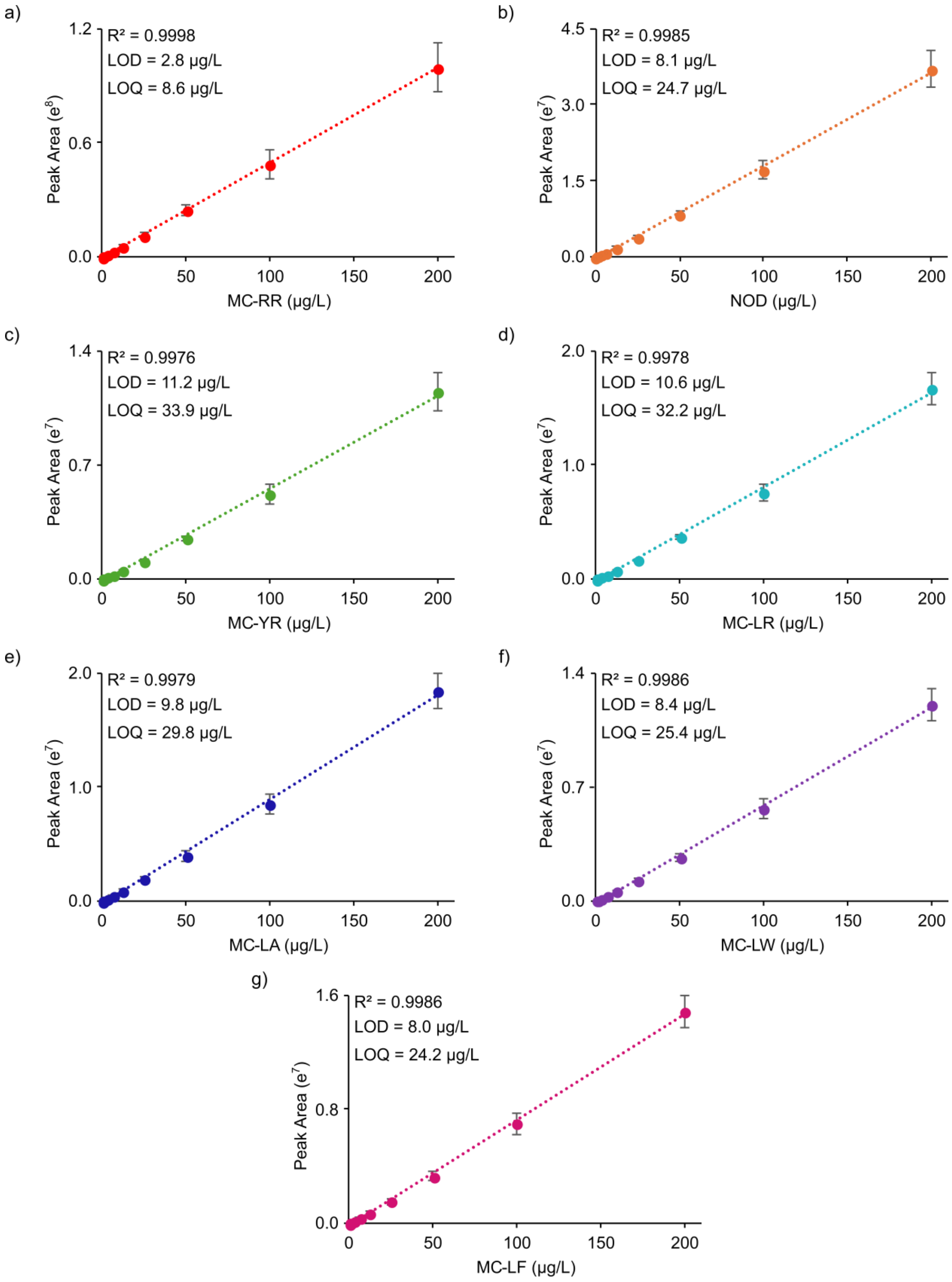

Figure S24: Calibration curves of the cyanotoxins MC-RR (a), NOD (b), MC-YR (c), MC-LR (d), MC-LA (e), MC-LW (f) and MC-LF (g). Standard deviation (n=3) is plotted alongside the regression of the line, the limit of detection (LOD) and limit of quantification (LOQ).

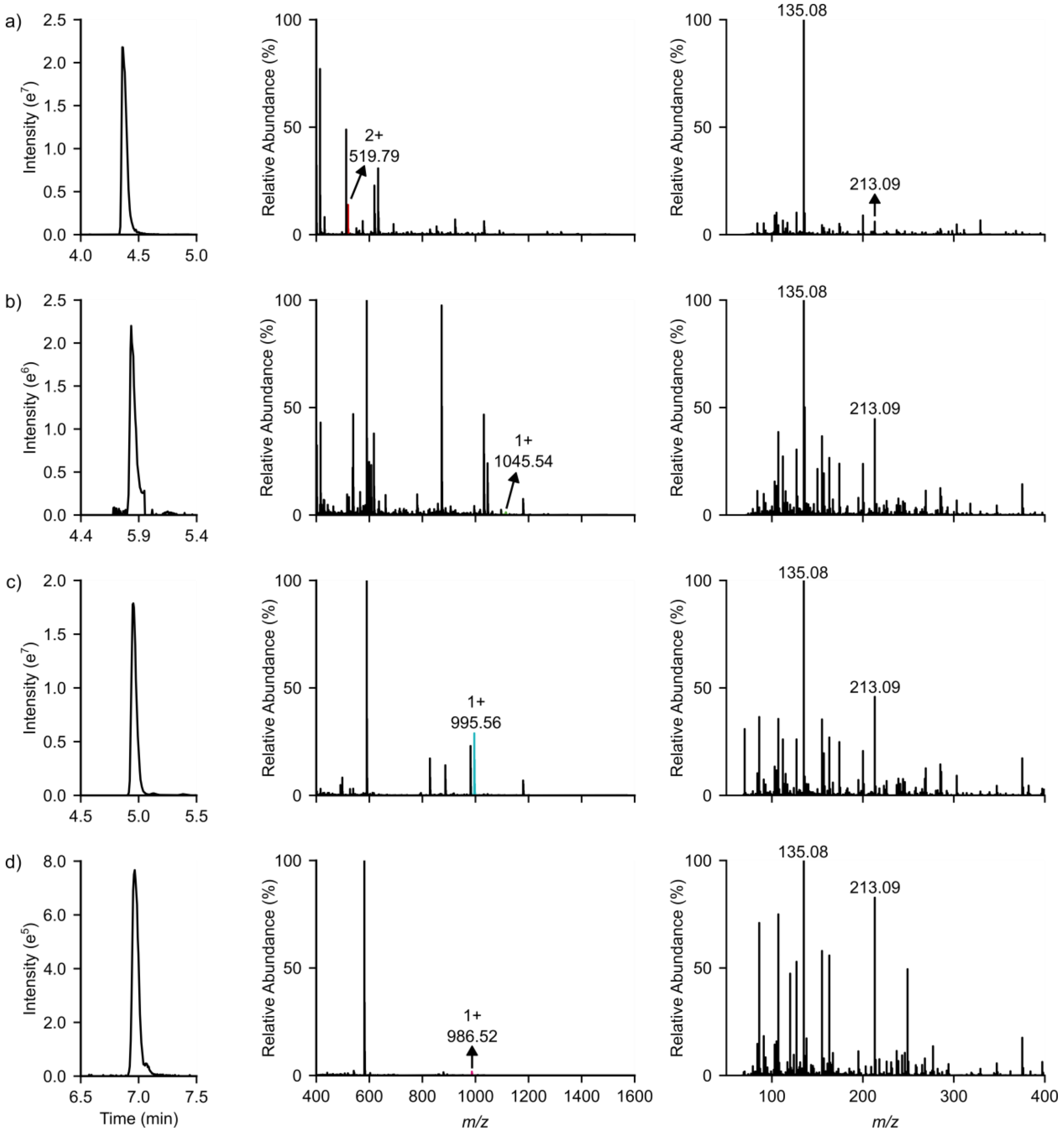

Figure S25: Chromatography (left), mass spectra (middle) and MS/MS (right) of MC-RR (a), MC-YR (b), MC-LR (c) and MC-LF (d) in lake water 6.

Table S1: Location of lake samples taken for analysis**.** An indication of cyanobacteria and the enumeration of cyanobacterial cells was shown by the absorbance of light at 750 nm and flow cytometry, respectively. * indicates where the lake sample was diluted 1000-fold before absorbance and flow cytometry measurements.

**Lake Site**	**Lake Location (UK)**	**Sampling Date**	**OD_{750}**	**Flow Cytometry (cells/mL)**
Lake water 1	Nottinghamshire	August 2024	0.11	72,500
Lake water 2	Leicestershire	August 2024	0.07	661,000
Lake water 3	Warwickshire	August 2024	0.03	162,000
Lake water 4	Leicestershire	August 2024	0.03	8,160
Lake water 5	Warwickshire	September 2024	0.10	193,000
Lake water 6	West Midlands	September 2024	0.10*	117,000*

Table S2: Experimental parameters for native MS detection of phycobiliprotein complexes from lake water. The parameters are specifically related to the instrument and the MS1 experiments performed.

**Native MS parameter**	**Instrument Setting**
Ionisation mode	Positive
Application mode	Intact protein
Pressure	High
In-source fragmentation	0
RF lens	120
Automatic gain control	100 %
Maximum injection time	100 ms
Microscans	10
Mass Analyzer	Orbitrap
m/z range	1,000-8,000 m/z
Resolution	7,500 at 200 m/z

Table S3: Phycobiliprotein dimers observed within the lake samples were matched to allophycocyanin (green) and phycocyanin (blue) dimers from known cyanobacterial strains calculated using the UniProt database. All matches were confirmed within ± 2 Da. * indicates where the matching mass was calculated with an additional Met-loss on the phycobiliprotein β subunit. # indicates species also detected in bottom-up proteomics (Note that the strain may differ).

**Lake 1**
**35455.7 Da**
Desmonostoc muscorum LEGE 12446*
**35577.6 Da**
Microcystis aeruginosa NIES-843 / IAM M-2473, NIES-2520, NIES-298, NIES-3787, NIES-3804, NIES-3807, NIES-4285, PCC 9701, PCC 9809, TAIHU98, PCC 9443*, PCC 9806*, PCC 9807*<br># ***Microcystis viridis*** NIES-102<br>***aff. Roholtiella sp.*** LEGE 12411*<br># ***Nostoc sp.*** PCC 7120 / SAG 25.82 / UTEX 2576*<br>***Gloeobacter kilaueensis*** ATCC BAA-2537 / CCAP 1431/1 / ULC 316 / JS1*<br># ***Pseudanabaena sp.*** lw0831*
**35666.6 Da**
Microcystis aeruginosa NIES-2519, PCC 9717, Sj<br># ***Microcystis wesenbergii*** Mw_MB_S_20031200_S109D<br># ***Chrysosporum bergii*** ANA360D*
**36790.3 Da**
Microchaete diplosiphon (*Fremyella diplosiphon*)*
**36862.3 Da**
Amazonocrinis nigriterrae CENA67*
**37412.0 Da**
filamentous cyanobacterium LEGE 07170
Microcystis aeruginosa PCC 9701, PCC 9432
**37457.2 Da**
Microcystis aeruginosa NIES-3804, PCC 9806, PCC 9808
**Lake 2**
**35709.8 Da**
Gloeobacter kilaueensis ATCC BAA-2537 / CCAP 1431/1 / ULC 316 / JS1<br>***Microcystis aeruginosa*** PCC 9443, PCC 9806, PCC 9807<br># ***Pseudanabaena sp.*** lw0831<br># ***Synechococcus sp.*** BIOS-U3-1*, BIOS-E4-1*, MIT S9504*
**36006.1 Da**
#***Oculatella sp.*** LEGE 06141
**Lake 3**
**36041.0 Da**
Chroococcidiopsis cubana SAG 39.79*
**36241.8 Da**
Pseudanabaena tenuis PCC 7409<br>***Phormidium rubidum****
**35975.4 Da**
Rivularia sp.<br>***Synechococcus sp.*** CC9311*

**37454.7 Da**
Microcystis aeruginosa NIES-843 / IAM M-2473, PCC 7806, NIES-4285, PCC 9809, TAIHU98, PCC 9717, NIES-3804, PCC 9806, PCC 9808<br>***Microcystis viridis*** NIES-102
**Lake 4**
**36843.7 Da**
Leptolyngbya boryana CZ1*
Arthrospira platensis qy3*<br>***Anabaena sp.*** 90*
**37486.7 Da**
Synechocystis sp. ATCC 27184 / PCC 6803 / Kazusa<br>***Chroococcidiopsis cubana*** SAG 39.79*<br>***Nostoc sp.*** PCC 7120 / SAG 25.82 / UTEX 2576*
**Lake 5**
**No strain matches at dimer level**
**Lake 6**
**36041.8 Da**
Chroococcidiopsis cubana SAG 39.79*
**36842.7 Da**
**# *Leptolyngbya boryana*** CZ1*
Arthrospira platensis qy3*<br>**# *Anabaena sp.*** 90*
**37454.3 Da**
**# *Microcystis aeruginosa*** NIES-843 / IAM M-2473, PCC 7806, NIES-4285, PCC 9809, TAIHU98, PCC 9717, NIES-3804, PCC 9806, PCC 9808<br>**# *Microcystis viridis*** NIES-102

Table S4: Phycobiliprotein monomers observed within the lake samples were matched **to** allophycocyanin (green) and phycocyanin (blue) monomers from known cyanobacterial strains calculated using the UniProt database. All matches were confirmed within ± 2 Da. * indicates where the matching mass was calculated with an additional Met-loss on the phycobiliprotein β subunit. # indicates species also detected in bottom-up proteomics (Note that these may not be the same identified strain).

	**Lake 1**	
**Subunit**	**17551.5 Da**	
	**α-APC**	# ***Anabaena sp.*** 90, AL09, LE011-02, MDT14b<br># ***Dolichospermum flos-aquae*** CCAP 1403/13F<br># ***Dolichospermum sp.*** DET73, UHCC 0260, UHCC 0315A, WA123<br># ***Nostocales*** cyanobacterium
	**17606.9 Da**	
	**α-APC**	# ***Anabaena sp.*** UBA12330, CRKS33<br># ***Aphanizomenon flos-aquae*** LD13, WA102<br># ***Nodosilinea sp.*** LEGE 07088
	**17774.0 Da**	
	**α-APC**	# ***Synechococcus sp.*** A15-60, A18-25c, BIOS-U3-1, MIT S9220, MIT S9508, NOUM97013<br># ***Microcystis aeruginosa*** NIES-843 / IAM M-2473, 11-30S32, DA14, KW, Ma_OC_H_19870700_S124, Ma_QC_B_20070730_S2, NIES-2519, NIES-2520, NIES-2521, NIES-2549, NIES-298, NIES-3787, NIES-3804, NIES-3807, NIES-4285, NIES-4325, NIES-44, PCC 9701, PCC 9717, PCC 9809, Sj, TAIHU98<br># ***Microcystis flos-aquae*** Mf_QC_C_20070823_S10D, TF09<br># ***Microcystis sp.*** 0824, M_QC_C_20170808_M3Col, M_QC_C_20170808_M9Col, MC19<br># ***Microcystis viridis*** NIES-102<br># ***Microcystis wesenbergii*** Mw_MB_S_20031200_S109D, Mw_QC_S_20081001_S30D, TW10
	**α-PC**	# Cyanobacteria bacterium UBA9273<br># **Phormidesmis sp.** RL_2_1<br># **Merismopedia glauca** CCAP 1448/3
	**β-APC**	# ***Oxynema aestuarii*** AP17*<br># ***Richelia sinica*** FACHB-800*<br># ***Nostocales*** cyanobacterium*<br># ***Nostoc sp.*** NIES-2111*, PCC 7120 / SAG 25.82 / UTEX 2576*<br># ***Trichormus variabilis*** NIES-23*, ATCC 29413 / PCC 7937*<br># ***Anabaena sp.*** YBS01*<br># ***Leptolyngbya sp.*** NIES-2104*, FACHB-16*<br># ***Synechococcaceae*** cyanobacterium SM2_3_2*<br># ***Nostoc azollae*** 0708*
	**18089.3 Da**	
	**α-APC**	# ***Leptolyngbya sp.*** LCM1.Bin17
	**α-PC**	# Cyanobacteria bacterium REEB444, WB6_1B_304<br># ***Phormidium ambiguum*** IAM M-71<br># ***Microcystis aeruginosa*** 11-30S32, NIES-2519, NIES-2520, BLCC-F158, PCC 9701<br># ***Microcystis sp.*** M_QC_C_20170808_M9Col<br># ***Microcystis flos-aquae*** Mf_QC_C_20070823_S10D<br># ***Planktothrix tepida*** PCC 9214
	**β-APC**	# ***Cyanobium sp.*** PLM2.Bin73<br># Cyanobacteria bacterium J083<br># ***Nodosilinea sp.*** LEGE 07298*
	**18118.5 Da**	

	**α-APC**	# ***Synechococcus sp.*** JA-2-3B'a(2-13), JA-3-3Ab), 60AY4M2<br>***Lusitaniella coriacea*** LEGE 07157
	**α-PC**	# ***Hydrococcus rivularis*** NIES-593<br># ***Thermoleptolyngbya oregonensis*** NK1-22<br># ***Pleurocapsa sp.*** PCC 7327<br># ***Microcystis panniformis*** FACHB-1757<br># ***Microcystis aeruginosa*** Sj, Ma_MB_S_20031200_S102, Ma_MB_F_20061100_S20D, NIES-3804, BLCC-F108, PCC 9806, PCC 9807, PCC 9808<br># ***Microcystis sp.*** M_OC_Ca_00000000_C217Col, FACHB-SPT15<br># ***Microcystis wesenbergii*** Mw_QC_S_20081001_S30D<br># ***Microcystis flos-aquae*** Mf_WU_F_19750830_S460<br># ***Nostoc sp.*** CENA543
	**β-APC**	# ***Leptolyngbyaceae*** cyanobacterium SM2_5_2<br># ***Synechococcus sp.*** GFB01<br># ***Cyanobium sp.*** NIES-981, PCC 7001
	**Lake 2**	
	**17622.8 Da**	
	**α-APC**	# ***Chlorogloeopsis fritschii*** PCC 6912<br># ***Aphanizomenon sp.*** UHCC 0183<br># ***Leptolyngbyaceae*** cyanobacterium RM1_405_57<br>***Nodosilinea sp.*** LEGE 07298
	**17724.0 Da**	
	**α-APC**	# ***Leptolyngbya boryana*** CZ1, NIES-2135
	**β-APC**	***Alkalinema sp.*** RU_4_3*<br># ***Pseudanabaena sp.*** lw0831*<br># ***Thermocoleostomius sinensis*** A174*
	**17855.1 Da**	
	**α-APC**	# ***Pseudanabaena sp.***<br># ***Synechococcus sp.*** A15-44, BS301-5m-G53, M16.1, RS9902, RS9907, TAK9802<br># ***Myxacorys almedinensis*** A<br># ***Symploca sp.*** SIO1C4, SIO3C6<br># ***Fortiea sp.*** LEGE XX443<br># ***Pseudanabaena sp.*** lw0831, SR411, UWO311<br># ***Leptolyngbya sp.*** NIES-2104<br># ***Richelia intracellularis*** HH01, HM01<br># ***Richelia sp.***<br># ***Synechococcus sp.*** CC9902
	**α-PC**	# ***Leptolyngbyaceae*** cyanobacterium SU_3_3, CSU_1_3<br># ***Calothrix sp.*** 336/3
	**β-APC**	***Alkalinema sp.*** RU_4_3<br># ***Pseudanabaena sp.*** lw0831<br># ***Thermocoleostomius sinensis*** A174<br># ***Synechococcales*** cyanobacterium RM1_1_8*<br>***Iningainema tapete*** BLCC-T55*
	**Lake 3**	
	**17613.6 Da**	
	**α-APC**	***Sphaerospermopsis sp.*** SIO1G1

	**α-PC**	***Anabaena* sp.** CRKS33<br>***Dolichospermum planctonicum***
	**18102.0 Da**	
	**α-PC**	***Calothrix parasitica*** NIES-267<br>***Nodularia* sp.**<br>***Microcystis flos-aquae*** TF09<br>***Microcystis aeruginosa*** DA14, NIES-4285, Ma_QC_Ca_00000000_S207, Ma_QC_Ch_20071001_S25D, FD4, PCC 7806, NIES-843 / IAM M-2473, PCC 9809, TAIHU98, SPC777, Ma_QC_B_20070730_S2, PCC 9717<br>***Microcystis viridis*** NIES-102, Mv_BB_P_19951000_S68D<br>***Microcystis* sp.** M_QC_C_20170808_M3Col, Msp_OC_L_20101000_S702<br>***Kamptonema* sp.** PCC 6506<br>***Hormoscilla* sp.** GUM202<br>***Cyanothece* sp.** ATCC 51142
	**β-APC**	***Synechococcaceae*** bacterium WB8_1B_136<br>***Fischerella* sp.** NIES-3754*<br>***Fischerella major*** NIES-592*<br>***Fischerella thermalis*** CCMEE 5330*, JSC-11*
	**19351.7 Da**	
	**β-APC**	***Atlanticothrix silvestris*** CENA357<br>***Lyngbya confervoides*** BDU141951<br>***Anabaena sphaerica*** FACHB-251
	**β-PC**	***Calothrix* sp.** C42_A2020_038<br>***Synechococcus* sp.** SynAce01<br>***Microcystis aeruginosa*** KW, NIES-298, DA14, NIES-4285, Ma_QC_C_20070703_M131, Ma_SC_T_19800800_S464, Ma_QC_B_20070730_S2, NIES-2520, PCC 9432, BLCC-F158, PCC 7806, NIES-843 / IAM M-2473, PCC 9717, PCC 9809, TAIHU98, SPC777,Ma_MB_S_20031200_S102<br>***Microcystis flos-aquae*** TF09<br>***Microcystis viridis*** NIES-102<br>***Microcystis* sp.** Msp_OC_L_20101000_S702<br>***Microcystis novacekii*** Mn_MB_F_20050700_S1D<br>***Microcystis wesenbergii*** Mw_MB_S_20031200_S109D<br>***Microcystis panniformis*** Mp_MB_F_20051200_S9<br>***Symploca* sp.** SIO2E9<br>***Planktothricoides* sp.** SpSt-374<br>***Anabaena sphaerica*** FACHB-251<br>***Leptolyngbyaceae*** cyanobacterium CSU_1_4<br>***Oscillatoriaceae*** cyanobacterium M33_DOE_052<br>***Nostoc* sp.** KVJ20<br>***Nostoc edaphicum*** CCNP1411<br>Cyanobacteria bacterium UBA11372*, CRU_2_1*<br>***Romeriopsis navalis*** LEGE 11480*
	**Lake 4**	
	**17551.8 Da**	
	**α-APC**	***Anabaena* sp.** 90, AL09, LE011-02, MDT14b<br>***Dolichospermum flos-aquae*** CCAP 1403/13F<br>***Dolichospermum* sp.** DET73, UHCC 0260, UHCC 0315A, WA123<br>***Nostocales*** cyanobacterium
	**17774.0 Da**	

	**α-APC**	***Synechococcus sp.*** A15-60, A18-25c, BIOS-U3-1, MIT S9220, MIT S9508, NOUM97013<br># ***Microcystis aeruginosa*** NIES-843 / IAM M-2473, 11-30S32, DA14, KW, Ma_OC_H_19870700_S124, Ma_QC_B_20070730_S2, NIES-2519, NIES-2520, NIES-2521, NIES-2549, NIES-298, NIES-3787, NIES-3804, NIES-3807, NIES-4285, NIES-4325, NIES-44, PCC 9701, PCC 9717, PCC 9809, Sj, TAIHU98<br># ***Microcystis flos-aquae*** Mf_QC_C_20070823_S10D, TF09<br># ***Microcystis sp.*** 0824, M_QC_C_20170808_M3Col, M_QC_C_20170808_M9Col, MC19<br># ***Microcystis viridis*** NIES-102<br># ***Microcystis wesenbergii*** Mw_MB_S_20031200_S109D, Mw_QC_S_20081001_S30D, TW10
	**α-PC**	Cyanobacteria bacterium UBA9273<br>***Phormidesmis sp.*** RL_2_1<br>***Merismopedia glauca*** CCAP 1448/3
	**β-APC**	***Richelia sinica*** FACHB-800*<br>***Nostocales*** cyanobacterium*<br>***Nostoc sp.*** NIES-2111*, PCC 7120 / SAG 25.82 / UTEX 2576*<br>***Trichormus variabilis*** NIES-23*, ATCC 29413 / PCC 7937*<br>***Anabaena sp.*** YBS01*<br>***Leptolyngbya sp.*** NIES-2104*, FACHB-16*<br>***Synechococcaceae*** cyanobacterium SM2_3_2*<br>***Nostoc azollae*** 0708*
	**18147.1 Da**	
	**α-PC**	***Synechococcales*** cyanobacterium C42_A2020_086, K44_A2020_017<br>***Leptolyngbya sp.*** DLM2.Bin15<br>***Phormidium sp.*** OSCR, SL48-SHIP<br>***Spirulina major***
	**β-APC**	***Cyanobium sp.***<br>***Leptolyngbya sp.*** SIO1E4
	**18452.9 Da**	
	**α-APC**	***Phormidesmis sp.*** LEGE 11477<br>***Plectonema cf. radiosum*** LEGE 06105<br>***Microcoleaceae*** cyanobacterium UBA11344<br>***Microcoleus sp.*** CSU_2_2<br>***Scytonema hofmannii*** PCC 7110<br>***Tychonema bourrellyi*** FEM_GT703<br>Cyanobacteria bacterium CRU_2_1, RU_5_0<br>***Fischerella thermalis*** CCMEE 5318<br># ***Microcystis aeruginosa*** NIES-843 / IAM M-2473, G11-04, Ma_QC_Ca_00000000_S207, Ma_QC_Ch_20071001_S25D, Ma_SC_T_19800800_S464<br># ***Microcystis sp.*** M_QC_C_20170808_M3Col, M_QC_C_20170808_M9Col
	**β-APC**	# ***Pleurocapsales*** cyanobacterium LEGE 10410
	**Lake 5**	
	**17606.1 Da**	
	**α-APC**	# ***Anabaena sp.*** UBA12330, CRKS33<br># ***Aphanizomenon flos-aquae*** LD13, WA102<br># ***Nodosilinea sp.*** LEGE 07088
	**Lake 6**	
	**17507.4 Da**	
	**α-APC**	# ***Cuspidothrix issatschenkoi*** CHARLIE-1<br>***Woronichinia naegeliana*** WA131

	**17551.1 Da**	
	**α-APC**	# ***Anabaena sp.*** 90, AL09, LE011-02, MDT14b<br># ***Dolichospermum flos-aquae*** CCAP 1403/13F<br># ***Dolichospermum sp.*** DET73, UHCC 0260, UHCC 0315A, WA123
	**17621.1 Da**	
	**α-APC**	# ***Anabaena sp.*** WA113<br>***Chlorogloeopsis fritschii*** PCC 6912
	**α-PC**	# ***Aphanizomenon flos-aquae*** WA102<br># ***Aphanizomenon sp.*** UHCC 0183
	**17664.2 Da**	
	**α-PC**	# uncultured ***Anabaena sp***<br># ***Anabaena*** sp. 90<br># ***Dolichospermum flos-aquae*** CCAP 1403/13F<br># ***Dolichospermum sp.*** UHCC 0260, WA123
	**β-APC**	# ***Mojavia pulchra*** JT2-VF2*
	**17817.5 Da**	
	**α-APC**	***Limnothrix rosea*** IAM M-220<br># ***Calothrix sp.*** C42_A2020_038<br>***Cyanobium gracile*** ATCC 27147 / PCC 6307<br># ***Microcystis aeruginosa*** PCC 7806, BLCC-F108, BLCC-F158, EAWAG127a, FD4, Ma_MB_F_20061100_S20D, Ma_MB_S_20031200_S102, Ma_QC_C_20070703_M131, Ma_SC_T_19800800_S464, PCC 9432, PCC 9443, PCC 9806, PCC 9807, PCC 9808, SPC777<br># ***Microcystis flos-aquae*** Mf_WU_F_19750830_S460<br># ***Microcystis novacekii*** Mn_MB_F_20050700_S1D<br># ***Microcystis panniformis*** FACHB-1757<br># ***Microcystis sp.*** M_OC_Ca_00000000_C217Col, Msp_OC_L_20101000_S702<br># ***Microcystis viridis*** Mv_BB_P_19951000_S68D<br># ***Nostoc sp.*** T09<br># ***Synechococcus sp.*** MED-G71
	**α-PC**	# ***Synechococcus sp.*** MIT S9508<br># Cyanobacteria bacterium UBA11367<br>***Pseudanabaena cinerea*** FACHB-1277<br>***Romeriopsis navalis*** LEGE 11480
	**β-APC**	# ***Leptolyngbya sp.*** FACHB-261<br># ***Synechocystis sp.*** B12, ATCC 27184 / PCC 6803 / Kazusa<br>***Okeania sp.*** KiyG1*<br># ***Leptolyngbya sp.*** NIES-3755*<br># ***Phormidesmis sp.*** RL_2_1*<br>***Brasilonema sennae*** CENA114*<br># ***Tolypothrix sp.*** NIES-4075*<br># ***Pseudanabaena sp.*** ABRG5-3*<br>***Hassalia byssoidea*** VB512170*

Table S5: Concentration of cyanotoxins in lake water samples 1-6. Instances where a cyanotoxin was not detected are denoted with 'ND'. '<LOD' indicates where the level of cyanotoxin fell below the limit of detection calculated from standard calibration curves but the presence of the cyanotoxin was verified manually by retention time, MS and MS/MS.

	Cyanotoxin concentration (µg/L)						
	MC-RR	NOD	MC-YR	MC-LR	MC-LA	MC-LW	MC-LF
Lake 1	ND	ND	<LOD	<LOD	<LOD	ND	ND
Lake 2	ND	ND	ND	ND	ND	ND	ND
Lake 3	<LOD	ND	ND	<LOD	ND	ND	ND
Lake 4	ND	ND	<LOD	<LOD	ND	ND	ND
Lake 5	<LOD	ND	ND	<LOD	ND	<LOD	<LOD
Lake 6	0.68	ND	0.66	3.45	<LOD	<LOD	0.21

Table S6: Inclusion list for triggering MS^2 data acquisition.

**Cyanotoxin**	**Expected** ***m/z***	**Charge state (*z*)**	**Retention Time Window (min)**
MC-RR	519.79	2	4.30 – 4.50
NOD-R	825.45	1	4.60 – 4.80
MC-YR	1045.54	1	4.75 – 4.95
MC-LR	995.56	1	4.85 – 5.05
MC-LA	910.49	1	5.95 – 6.15
MC-LW	1025.53	1	6.70 – 6.90
MC-LF	986.52	1	6.85 – 7.05
