# Supplementary material for: A Protein-Centric Mass Spectrometry Approach for Species Identification within Harmful Algal Blooms
Source: J Am Chem Soc. 2025 Jul 28;147(31):27974–80. doi: 10.1021/jacs.5c07419 (PMC12333325; doi:10.1021/jacs.5c07419)
Supplement: Supplementary file 3 [file ja5c07419_si_003.pdf]

<u>Species Identification as determined by bottom-up proteomics</u>

Table 1: Proteins from cyanobacterial strains detected by trypsin digestion followed by LC-MS/MS analysis from lake water 1.

Accession	Protein Name	Species	Coverage [%]	# Peptides	# PSMs
Acaryochloridaceae cyanobacterium					
A0A968YX85	Allophycocyanin	*Acaryochloridaceae cyanobacterium RU_4_10*	12	2	2
Aerosakkonema funiforme					
A0A926VEV9	Allophycocyanin	*Aerosakkonema funiforme FACHB-1375*	11	2	2
Aetokthonos hydrillicola					
A0AAP5I9Q5	Allophycocyanin subunit alpha	*Aetokthonos hydrillicola Thurmond2011*	32	5	5
A0AAP5I8M6	Phycocyanin subunit alpha	*Aetokthonos hydrillicola Thurmond2011*	10	2	6
A0AAP5I693	Phycocyanin subunit beta	*Aetokthonos hydrillicola Thurmond2011*	12	2	2
aff. Roholtiella sp.					
A0A8J7D6K0	Allophycocyanin subunit alpha	*aff. Roholtiella sp. LEGE 12411*	11	2	2
Aliterella atlantica					
A0A0D8ZXX5	Allophycocyanin	*Aliterella atlantica CENA595*	19	2	6
A0A0D8ZST4	Phycocyanin	*Aliterella atlantica CENA595*	10	2	5
A0A0D8ZUK5	Allophycocyanin	*Aliterella atlantica CENA595*	32	5	5
Alkalinema sp.					
A0A251WM95	Phycocyanin subunit alpha	*Alkalinema sp. CACIAM 70d*	19	3	7
A0A251WJA0	Allophycocyanin	*Alkalinema sp. CACIAM 70d*	12	2	2
A0A251WJT7	Allophycocyanin subunit beta	*Alkalinema sp. CACIAM 70d*	14	2	2
A0A251WDP1	Phycocyanin subunit alpha	*Alkalinema sp. CACIAM 70d*	10	2	6
A0A968X6G1	Allophycocyanin	*Alkalinema sp. RU_4_3*	12	3	3
A0A968X4F9	Allophycocyanin subunit beta	*Alkalinema sp. RU_4_3*	17	2	3
Amazonocrinis nigriterrae					
A0A8J7L6C9	Allophycocyanin subunit alpha	*Amazonocrinis nigriterrae CENA67*	19	2	2
Anabaena cylindrica					
P07325	Allophycocyanin alpha chain	*Anabaena cylindrica*	43	5	8
Anabaena sp.					
K7WMD9	Phycobilisome protein ApcA	*Anabaena sp. 90*	56	6	10
K7WTL4	Phycocyanin beta subunit	*Anabaena sp. 90*	61	8	10
A0A1B7V2J0	Phycocyanin	*Anabaena sp. AL09*	24	3	3
A0A1B7WMN4	Phycocyanin	*Anabaena sp. CRKS33*	52	7	10
A0A1B7WSR8	Allophycocyanin	*Anabaena sp. CRKS33*	57	6	10
A0A1B7WMQ4	Phycocyanin	*Anabaena sp. CRKS33*	49	7	14
A0A926ULC4	Allophycocyanin	*Anabaena sp. FACHB-1237*	42	5	8
A0A926UKC4	Allophycocyanin subunit beta	*Anabaena sp. FACHB-1237*	30	4	8
A0A1B7V0A1	Allophycocyanin	*Anabaena sp. LE011-02*	57	6	10
A0A9X1GP51	Allophycocyanin	*Anabaena sp. PCC 7938*	43	5	8
A0A9X1GQJ4	Phycocyanin subunit alpha	*Anabaena sp. PCC 7938*	23	2	2
A0A9X1GK69	Allophycocyanin subunit beta	*Anabaena sp. PCC 7938*	24	3	7
A0A3D4C397	Phycocyanin subunit alpha	*Anabaena sp. UBA12330*	34	4	7

A0A3D4C1K4	Allophycocyanin subunit beta	*Anabaena sp. UBA12330*	41	5	10
A0A1B7VSW7	Allophycocyanin	*Anabaena sp. WA113*	32	4	5
A0A455KZV8	Phycocyanin B	*uncultured Anabaena sp*	53	7	9
Anabaena sphaerica					
A0A927A4K0	Allophycocyanin	*Anabaena sphaerica FACHB-251*	50	5	9
A0A927A3E3	Allophycocyanin subunit beta	*Anabaena sphaerica FACHB-251*	24	3	7
A0A927A4I2	Phycocyanin subunit alpha	*Anabaena sphaerica FACHB-251*	19	3	6
A0A927A3U9	Phycocyanin subunit beta	*Anabaena sphaerica FACHB-251*	24	3	3
Anabaenopsis circularis					
A0A1Z4GCJ2	Phycobilisome protein	*Anabaenopsis circularis NIES-21*	17	3	3
Anabaenopsis elenkinii					
A0A7S6TZF6	Allophycocyanin subunit alpha	*Anabaenopsis elenkinii CCIBt3563*	26	4	4
A0A7S6RCY6	Phycocyanin subunit alpha	*Anabaenopsis elenkinii CCIBt3563*	17	2	2
A0A7S6RDD3	Phycocyanin subunit beta	*Anabaenopsis elenkinii CCIBt3563*	12	2	2
Anthocerotibacter panamensis					
A0AAJ6N6E0	ApcA2	*Anthocerotibacter panamensis*	11	2	2
Aphanizomenon flos-aquae					
A0A1B7WBM0	Phycocyanin	*Aphanizomenon flos-aquae WA102*	26	4	7
A0A1B7X4P2	Allophycocyanin	*Aphanizomenon flos-aquae WA102*	43	5	8
A0A1B7WZK6	Phycocyanin	*Aphanizomenon flos-aquae WA102*	48	6	8
Aphanizomenon sp.					
A0A844ICS2	Phycocyanin subunit beta	*Aphanizomenon sp. UHCC 0183*	36	5	5
Aphanothece hegewaldii					
A0A2T1LW90	Allophycocyanin subunit beta	*Aphanothece hegewaldii CCALA 016*	30	3	4
A0A2T1LW89	Allophycocyanin	*Aphanothece hegewaldii CCALA 016*	27	5	6
Aphanothece sacrum					
A0A401ICI1	Allophycocyanin b chain	*Aphanothece sacrum FPU1*	27	3	4
A0A401ICC4	Allophycocyanin subunit alpha apoprotein	*Aphanothece sacrum FPU1*	19	3	3
A0A401ILC5	Phycocyanin b subunit	*Aphanothece sacrum FPU1*	12	2	2
Arthrospira platensis					
Q208D0	Phycocyanin alpha chain	*Arthrospira platensis*	17	2	2
Atlanticothrix silvestris					
A0A8J7H5A3	Allophycocyanin subunit beta	*Atlanticothrix silvestris CENA357*	14	2	3
A0A8J7H708	Allophycocyanin subunit alpha	*Atlanticothrix silvestris CENA357*	25	3	3
Brunnivagina elsteri					
A0A2A2TIY1	Allophycocyanin subunit beta	*Brunnivagina elsteri CCALA 953*	14	2	3
A0A2A2TBP9	Phycocyanin subunit alpha	*Brunnivagina elsteri CCALA 953*	10	2	6
Calothrix sp.					
A0A0T7BME1	Allophycocyanin	*Calothrix sp. 336/3*	24	3	6
A0A0T7BMD2	Allophycocyanin	*Calothrix sp. 336/3*	25	3	3
A0A930XG57	Allophycocyanin subunit alpha	*Calothrix sp. C42 A2020 038*	26	3	3
A0A930T7Y5	Phycocyanin subunit alpha	*Calothrix sp. C42 A2020 038*	19	2	5
A0A9E5V8C2	Allophycocyanin	*Calothrix sp. CSU 2 0*	25	3	3
A0A9E5V5E7	Allophycocyanin subunit beta	*Calothrix sp. CSU 2 0*	14	2	3
A0A1Z4NJH9	Phycocyanin	*Calothrix sp. NIES-3974*	36	4	6
A0A1Z4NJK4	Allophycocyanin beta subunit	*Calothrix sp. NIES-3974*	14	2	3

A0A1Z4NQ20	C-phycocyanin-1 alpha chain	*Calothrix sp. NIES-3974*	10	2	6
A0A1Z4R810	Phycobilisome protein	*Calothrix sp. NIES-4101*	31	4	4
A0A1Z4RG19	Phycocyanin alpha subunit	*Calothrix sp. NIES-4101*	10	2	6
K9UXN4	Phycocyanin	*Calothrix sp. PCC 6303*	19	2	2
K9UZP4	Allophycocyanin, beta subunit	*Calothrix sp. PCC 6303*	14	2	3
K9V8G6	Phycocyanin, beta subunit	*Calothrix sp. PCC 6303*	12	2	2
Chamaesiphon polymorphus					
A0A2T1GIN7	Allophycocyanin	*Chamaesiphon polymorphus CCALA 037*	13	2	3
Chamaesiphon sp.					
A0A969QKP1	Allophycocyanin subunit beta	*Chamaesiphon sp. CSU_1_12*	19	2	5
A0A969QHW0	Allophycocyanin	*Chamaesiphon sp. CSU_1_12*	13	3	4
Chlorogloea sp.					
A0A2T1EA96	Phycocyanin subunit alpha	*Chlorogloea sp. CCALA 695*	10	2	5
A0A2T1EFR3	Allophycocyanin	*Chlorogloea sp. CCALA 695*	32	4	4
Chlorogloeopsis fritschii					
A0A3S0Y3A8	Allophycocyanin alpha chain	*Chlorogloeopsis fritschii PCC 6912*	25	3	3
A0A433NKR2	C-phycocyanin alpha chain	*Chlorogloeopsis fritschii PCC 6912*	23	2	2
Chondrocystis sp.					
A0A1Z4RRS0	Phycobilisome protein	*Chondrocystis sp. NIES-4102*	17	3	3
Chroococcidiopsis sp.					
A0A2P8QIZ5	Allophycocyanin	*Chroococcidiopsis sp. CCALA 051*	45	5	6
A0A2S6VGG8	Allophycocyanin	*Chroococcidiopsis sp. TS-821*	31	4	4
Chroococcus sp.					
A0A8J2TC53	Allophycocyanin, beta subunit	*Chroococcus sp. FPU101*	30	3	4
A0A8J2TI46	Phycobilisome protein	*Chroococcus sp. FPU101*	14	2	3
Chrysosporum bergii					
A0AA43GP68	Allophycocyanin subunit alpha	*Chrysosporum bergii ANA360D*	26	3	3
A0AA43KB61	Phycocyanin subunit alpha	*Chrysosporum bergii ANA360D*	17	2	2
Coleofasciculaceae cyanobacterium					
A0A968SN98	Allophycocyanin	*Coleofasciculaceae cyanobacterium SM2_1_6*	12	3	3
A0A968SHU1	Allophycocyanin subunit beta	*Coleofasciculaceae cyanobacterium SM2_1_6*	19	2	3
A0A968SF93	Phycocyanin subunit beta	*Coleofasciculaceae cyanobacterium SM2_1_6*	16	3	7
Coleofasciculus chthonoplastes					
B4VSN1	Allophycocyanin, beta subunit	*Coleofasciculus chthonoplastes PCC 7420*	24	3	6
B4VWT1	Phycocyanin, alpha subunit	*Coleofasciculus chthonoplastes PCC 7420*	19	3	7
Coleofasciculus sp.					
A0A926XI84	Allophycocyanin	*Coleofasciculus sp. FACHB-SPT36*	25	3	4
A0A929FQ32	Allophycocyanin subunit beta	*Coleofasciculus sp. LEGE 07092*	24	3	7
A0A929AQ34	Allophycocyanin subunit alpha	*Coleofasciculus sp. LEGE 07092*	12	2	2
A0A929AKM3	Phycocyanin subunit alpha	*Coleofasciculus sp. LEGE 07092*	10	2	6
A0A6J4IPJ0	Allophycocyanin alpha chain	*uncultured Coleofasciculus sp*	20	2	3
Cuspidothrix issatschenkoi					
A0A2S6CX50	Allophycocyanin	*Cuspidothrix issatschenkoi CHARLIE-1*	31	4	5
A0A2S6CQK6	Phycocyanin subunit alpha	*Cuspidothrix issatschenkoi CHARLIE-1*	31	3	3
A0A2S6CQK9	Phycocyanin subunit beta	*Cuspidothrix issatschenkoi CHARLIE-1*	45	6	8
Cyanobacteria bacterium					

A0A966F8Q4	Allophycocyanin subunit beta	*Cyanobacteria bacterium CG_2015-22_32_23*	19	2	3
A0A926Y5C9	Allophycocyanin subunit beta	*Cyanobacteria bacterium FACHB-502*	19	2	2
A0A926PQ48	Allophycocyanin	*Cyanobacteria bacterium FACHB-DQ100*	11	2	2
A0A3M1P8I6	Allophycocyanin subunit beta	*Cyanobacteria bacterium J069*	19	2	5
A0A3M1PIT8	Phycocyanin subunit alpha	*Cyanobacteria bacterium J069*	23	2	2
A0A3M1P9Q4	Allophycocyanin	*Cyanobacteria bacterium J069*	11	2	2
A0A3M1L8Y9	Phycocyanin subunit beta	*Cyanobacteria bacterium J083*	12	2	2
A0A2N5JMK4	Allophycocyanin	*Cyanobacteria bacterium M5B4*	19	3	3
A0A2T2RRT4	Allophycocyanin	*Cyanobacteria bacterium QH_9_48_43*	33	5	6
A0A2T2RY45	Allophycocyanin subunit beta	*Cyanobacteria bacterium QS_8_64_29*	22	2	3
A0A2T2RXX7	Allophycocyanin	*Cyanobacteria bacterium QS_8_64_29*	33	5	6
A0A969LWT5	Phycocyanin subunit alpha	*Cyanobacteria bacterium RU_5_0*	23	2	2
A0A969LW31	Phycocyanin subunit beta	*Cyanobacteria bacterium RU_5_0*	12	2	2
A0A9D9KAG3	Allophycocyanin subunit beta	*Cyanobacteria bacterium SID2*	19	2	3
A0A9D9KD37	Allophycocyanin subunit alpha	*Cyanobacteria bacterium SID2*	7	2	2
A0A2T2RE55	Allophycocyanin	*Cyanobacteria bacterium SW_9_44_58*	33	5	6
A0A355DKD1	Allophycocyanin subunit beta	*Cyanobacteria bacterium UBA11162*	35	4	8
A0A355DJA3	Allophycocyanin	*Cyanobacteria bacterium UBA11162*	27	4	4
A0A351KYP4	Allophycocyanin	*Cyanobacteria bacterium UBA11371*	17	3	3
A0A350XIP0	Phycocyanin subunit alpha	*Cyanobacteria bacterium UBA11372*	17	2	3
A0A3B8K348	Phycocyanin subunit alpha	*Cyanobacteria bacterium UBA8553*	19	3	6
A0A352AEK5	Allophycocyanin subunit beta	*Cyanobacteria bacterium UBA9273*	24	3	6
A0A352AEK4	Allophycocyanin	*Cyanobacteria bacterium UBA9273*	25	3	3
A0A965DJM1	Allophycocyanin	*Cyanobacteria bacterium WB6_1B_304*	11	2	2
Cyanobacterium aponinum					
K9Z642	Allophycocyanin alpha subunit apoprotein	*Cyanobacterium aponinum (strain PCC 10605)*	11	2	2
K9Z660	Phycocyanin, alpha subunit	*Cyanobacterium aponinum (strain PCC 10605)*	17	2	2
A0AAF0ZF69	Allophycocyanin subunit beta	*Cyanobacterium aponinum AL20115*	19	2	3
A0AAF1C585	Phycocyanin subunit beta	*Cyanobacterium aponinum AL20115*	12	2	2
Cyanobacterium sp.					
A0A2K8WSI2	Allophycocyanin beta subunit ApcB	*Cyanobacterium sp. HL-69*	19	2	3
A0A2K8WQM9	Phycocyanin beta subunit CpcB	*Cyanobacterium sp. HL-69*	12	2	2
A0A1E5QZJ0	Allophycocyanin	*Cyanobacterium sp. IPPAS B-1200*	22	3	4
A0A930SX45	Allophycocyanin subunit beta	*Cyanobacterium sp. T60_A2020_053*	19	2	3
A0A930T0W6	Allophycocyanin	*Cyanobacterium sp. T60_A2020_053*	16	2	3
A0A930XCN1	Phycocyanin subunit beta	*Cyanobacterium sp. T60_A2020_053*	12	2	2
Cyanobacterium stanieri					
K9YI32	Allophycocyanin, beta subunit	*Cyanobacterium stanieri (strain ATCC 29140 / PCC 7202)*	19	2	3
Cyanobium sp.					
B5IPK3	Phycocyanin, alpha subunit	*Cyanobium sp. PCC 7001*	10	2	6
Cyanomargarita calcarea					
A0A951QKN4	Allophycocyanin subunit alpha	*Cyanomargarita calcarea GSE-NOS-MK-12-04C*	26	4	4
A0A951QUE8	Phycocyanin subunit beta	*Cyanomargarita calcarea GSE-NOS-MK-12-04C*	12	2	2
Cyanothece sp.					
A0A3B8XYR2	Allophycocyanin subunit beta	*Cyanothece sp. UBA12306*	22	2	6

A0A3B8Y2T7	Allophycocyanin	*Cyanothece sp. UBA12306*	13	2	2
Cylindrospermopsis curvispora					
A0A7H0F0K5	Allophycocyanin	*Cylindrospermopsis curvispora GIHE-G1*	37	5	7
A0A7H0EY98	Phycocyanin subunit alpha	*Cylindrospermopsis curvispora GIHE-G1*	23	2	2
A0A7H0EY99	Phycocyanin subunit beta	*Cylindrospermopsis curvispora GIHE-G1*	17	2	2
Cylindrospermopsis raciborskii					
A0A9Q5QUG8	Allophycocyanin	*Cylindrospermopsis raciborskii CENA302*	43	5	7
A0A9Q5QZI3	Phycocyanin subunit alpha	*Cylindrospermopsis raciborskii CENA302*	19	2	2
A0A1X4G9K1	Phycocyanin subunit beta	*Cylindrospermopsis raciborskii CENA303*	17	2	2
A0A838WHR3	Allophycocyanin	*Cylindrospermopsis raciborskii CS-506_A*	25	3	3
A0A838WVU6	Allophycocyanin subunit beta	*Cylindrospermopsis raciborskii CS-506_A*	24	3	6
A0A838WGC8	Phycocyanin subunit beta	*Cylindrospermopsis raciborskii CS-506_A*	9	2	2
Cylindrospermopsis sp.					
A0A0R0MH79	Allophycocyanin	*Cylindrospermopsis sp. CR12*	37	5	7
A0A0R0M5Z0	Phycocyanin	*Cylindrospermopsis sp. CR12*	23	2	2
Cylindrospermum sp.					
A0A926WTG6	Allophycocyanin	*Cylindrospermum sp. FACHB-282*	31	4	5
A0A1Z4QKC3	Phycobilisome protein	*Cylindrospermum sp. NIES-4074*	17	3	4
Cylindrospermum stagnale					
K9WSW2	Allophycocyanin alpha subunit apoprotein	*Cylindrospermum stagnale PCC 7417*	12	2	3
Dendronalium phyllosphericum					
A0A8J7I918	Allophycocyanin subunit alpha	*Dendronalium phyllosphericum CENA369*	19	2	2
Desertifilum sp.					
A0A846E286	Allophycocyanin	*Desertifilum sp. SIO1I2*	12	2	2
Desmonostoc muscorum					
A0A8J6ZVU6	Allophycocyanin subunit alpha	*Desmonostoc muscorum LEGE 12446*	12	2	3
Dolichospermum compactum					
A0A1Z4VAR2	Phycocyanin alpha subunit	*Dolichospermum compactum NIES-806*	41	5	8
A0A1Z4V5K5	Phycocyanin	*Dolichospermum compactum NIES-806*	56	6	10
A0A1Z4V5I7	Allophycocyanin beta subunit	*Dolichospermum compactum NIES-806*	24	3	7
A0A1Z4VAT7	Phycocyanin beta subunit	*Dolichospermum compactum NIES-806*	36	5	5
Dolichospermum flos-aquae					
A0A6H2C1Q0	Phycocyanin subunit alpha	*Dolichospermum flos-aquae CCAP 1403/13F*	36	5	8
Dolichospermum sp.					
A0AAW6JTW9	Phycocyanin subunit alpha	*Dolichospermum sp. ST_sed8*	36	5	8
A0AAW6JT81	Allophycocyanin subunit alpha	*Dolichospermum sp. ST_sed8*	57	6	10
A0AAW6JT84	Allophycocyanin subunit beta	*Dolichospermum sp. ST_sed8*	24	3	7
A0A844IMK7	Allophycocyanin subunit beta	*Dolichospermum sp. UHCC 0260*	24	3	7
A0A5C0DQG9	C-phycocyanin alpha chain	*Dolichospermum sp. UHCC 0315A*	36	5	8
A0A5C0DNG5	C-phycocyanin beta chain	*Dolichospermum sp. UHCC 0315A*	36	5	7
Drouetiella hepatica					
A0A951Q8B1	Allophycocyanin subunit alpha	*Drouetiella hepatica Uher 2000/2452*	12	3	3
Dulcicalothrix desertica					
A0A3S1CAQ3	Allophycocyanin beta chain	*Dulcicalothrix desertica PCC 7102*	24	3	7
A0A433VBJ0	Allophycocyanin alpha chain	*Dulcicalothrix desertica PCC 7102*	25	3	3
filamentous cyanobacterium					

A0A2T1DPM6	Allophycocyanin subunit beta	*filamentous cyanobacterium CCP2*	19	2	2
A0A2T1DPM3	Allophycocyanin	*filamentous cyanobacterium CCP2*	12	2	2
A0A2T1D683	Phycocyanin subunit alpha	*filamentous cyanobacterium CCP2*	23	2	2
A0A2T2WBC5	Allophycocyanin	*filamentous cyanobacterium CCP3*	26	3	4
A0A2T2WB84	Allophycocyanin subunit beta	*filamentous cyanobacterium CCP3*	19	2	5
A0A2P8WFG0	Allophycocyanin	*filamentous cyanobacterium CCP5*	26	3	4
A0A929A3S2	Allophycocyanin subunit beta	*filamentous cyanobacterium LEGE 07170*	14	2	2
A0A929A5X8	Phycocyanin subunit alpha	*filamentous cyanobacterium LEGE 07170*	19	3	7
A0A929FD76	Allophycocyanin subunit alpha	*filamentous cyanobacterium LEGE 07170*	12	2	2
A0A929FFA0	Phycocyanin subunit alpha	*filamentous cyanobacterium LEGE 07170*	10	2	6
A0A2T1EZJ2	Allophycocyanin	*filamentous cyanobacterium Phorm 46*	19	3	3
Fischerella major					
A0A1U7H0W4	Allophycocyanin	*Fischerella major NIES-592*	25	3	4
Fischerella muscicola					
A0A2N6K780	Allophycocyanin	*Fischerella muscicola CCMEE 5323*	25	3	4
Fischerella thermalis					
A0A2N6KBI8	Allophycocyanin	*Fischerella thermalis CCMEE 5268*	25	3	4
G6FU34	Phycocyanin	*Fischerella thermalis JSC-11*	31	4	5
Fortiea sp.					
A0A8J7DCH0	Allophycocyanin subunit beta	*Fortiea sp. LEGE XX443*	19	2	6
A0A8J7A5B9	Allophycocyanin subunit alpha	*Fortiea sp. LEGE XX443*	30	3	5
A0A8J7D8J8	Phycocyanin subunit alpha	*Fortiea sp. LEGE XX443*	23	2	2
Geitlerinema sp.					
A0A1Y5QE20	Phycocyanin alpha subunit	*Geitlerinema sp. H8DM*	10	2	6
Geminocystis sp.					
A0A978T651	Allophycocyanin	*Geminocystis sp. M7585 C2015 104*	27	5	7
A0A0D6AHF2	Allophycocyanin alpha chain	*Geminocystis sp. NIES-3708*	12	2	2
A0A0D6ANS0	Allophycocyanin beta chain	*Geminocystis sp. NIES-3709*	19	2	3
A0A0D6ANR5	Allophycocyanin alpha chain	*Geminocystis sp. NIES-3709*	19	4	4
Gloeobacter kilaueensis					
U5QFD5	Allophycocyanin, beta subunit	*Gloeobacter kilaueensis (strain ATCC BAA-2537 / CCAP 1431/1 / ULC 316 / JS1)*	12	3	3
Gloeobacter violaceus					
Q7NL80	Allophycocyanin alpha subunit	*Gloeobacter violaceus (strain ATCC 29082 / PCC 7421)*	19	4	5
Gloeobacterales cyanobacterium					
A0A925XSP1	Allophycocyanin	*Gloeobacterales cyanobacterium ES-bin-141*	17	3	4
A0A925ISE1	Allophycocyanin	*Gloeobacterales cyanobacterium ES-bin-313*	17	3	4
Gloeocapsa sp.					
A0A6P2AG54	Phycocyanin subunit alpha	*Gloeocapsa sp. DLM2.Bin57*	19	2	5
A0A6P2ACY3	Allophycocyanin subunit beta	*Gloeocapsa sp. DLM2.Bin57*	30	3	5
A0A6P2AEI5	Allophycocyanin	*Gloeocapsa sp. DLM2.Bin57*	12	3	3
L8LPE1	Allophycocyanin beta subunit apoprotein	*Gloeocapsa sp. PCC 73106*	19	2	3
Gloeocapsopsis dulcis					
A0A6N8G1A9	Allophycocyanin	*Gloeocapsopsis dulcis AAB1*	26	3	3
Gloeocapsopsis sp.					
A0A2G7GIJ4	Allophycocyanin	*Gloeocapsopsis sp. IPPAS B-1203*	31	4	4

Gloeomargaritaceae cyanobacterium					
A0A930U927	Allophycocyanin	*Gloeomargaritaceae cyanobacterium C42_A2020_066*	21	2	3
Gloeothece citriformis					
B7KKS9	Phycobilisome protein	*Gloeothece citriformis (strain PCC 7424)*	34	4	6
B7KKT0	Allophycocyanin, beta subunit	*Gloeothece citriformis (strain PCC 7424)*	30	3	4
Gloeothece verrucosa					
E0UA88	Phycocyanin	*Gloeothece verrucosa (strain PCC 7822)*	35	4	7
E0UA89	Allophycocyanin, beta subunit	*Gloeothece verrucosa (strain PCC 7822)*	30	3	4
E0U7E5	Phycocyanin, alpha subunit	*Gloeothece verrucosa (strain PCC 7822)*	17	2	2
Gomphosphaeria aponina					
A0A941GQA6	Allophycocyanin subunit beta	*Gomphosphaeria aponina SAG 52.96*	19	2	4
A0A941GXV2	Allophycocyanin subunit alpha	*Gomphosphaeria aponina SAG 52.96*	12	3	3
Hassallia byssoidea					
A0A846H2L7	Allophycocyanin	*Hassallia byssoidea VB512170*	25	3	4
Hydrococcus rivularis					
A0A1U7HQ10	Allophycocyanin subunit beta	*Hydrococcus rivularis NIES-593*	39	4	5
Hydrococcus sp.					
A0A968Z758	Allophycocyanin	*Hydrococcus sp. RU_2_2*	21	2	3
Kamptonema sp.					
A0A6P1AC41	Allophycocyanin subunit beta	*Kamptonema sp. SIO1D9*	19	2	2
A0A6P0JYL8	Allophycocyanin subunit beta	*Kamptonema sp. SIO4C4*	17	2	3
Leptolyngbya boryana					
A0AA96WUE2	Allophycocyanin subunit alpha	*Leptolyngbya boryana CZ1*	23	4	4
A0A1Z4JFC6	Allophycocyanin beta subunit	*Leptolyngbya boryana NIES-2135*	25	3	4
Leptolyngbya cf. ectocarpi					
A0A929FCN5	Allophycocyanin subunit alpha	*Leptolyngbya cf. ectocarpi LEGE 11479*	12	2	2
Leptolyngbya foveolarum					
A0A2W4WL51	Allophycocyanin	*Leptolyngbya foveolarum*	19	4	4
Leptolyngbya sp					
A0A2W7BS63	Allophycocyanin	*Leptolyngbya sp*	13	3	3
A0A2W7ATZ9	Allophycocyanin	*Leptolyngbya sp*	19	4	4
A0A2W7BV24	Allophycocyanin subunit beta	*Leptolyngbya sp*	19	2	5
A0A2W7A1E1	Allophycocyanin	*Leptolyngbya sp*	27	3	4
A0A7T5E5H0	Phycocyanin subunit alpha	*Leptolyngbya sp. BL0902*	10	2	6
A0A651EBC1	Allophycocyanin subunit beta	*Leptolyngbya sp. DLM2.Bin15*	19	2	5
A0A651DVI7	Allophycocyanin	*Leptolyngbya sp. DLM2.Bin15*	12	3	3
A0A5Q4EBV8	Allophycocyanin	*Leptolyngbya sp. DLM2.Bin27*	7	2	2
A0A926ZCU8	Allophycocyanin subunit beta	*Leptolyngbya sp. FACHB-16*	14	2	2
A0A926TNE1	Phycocyanin subunit alpha	*Leptolyngbya sp. FACHB-321*	18	2	3
A0A926YFT9	Allophycocyanin subunit beta	*Leptolyngbya sp. FACHB-36*	16	2	5
A0A926YFD0	Allophycocyanin	*Leptolyngbya sp. FACHB-36*	11	2	2
A0A926TEU7	Phycocyanin subunit alpha	*Leptolyngbya sp. FACHB-36*	10	2	5
A0A1Q8ZG86	Phycocyanin subunit alpha	*Leptolyngbya sp. 'hensonii'*	10	2	6
A0A1Q8ZG75	Phycocyanin subunit alpha	*Leptolyngbya sp. 'hensonii'*	19	3	7
A0A1Q8ZG15	Allophycocyanin	*Leptolyngbya sp. 'hensonii'*	19	3	3
U9VQP6	Allophycocyanin subunit alpha	*Leptolyngbya sp. Heron Island J*	11	2	2

U9VUN9	C-phycocyanin beta chain	*Leptolyngbya sp. Heron Island J*	12	2	2
A0A6H2NJD9	Allophycocyanin subunit beta	*Leptolyngbya sp. LCM1.Bin17*	19	2	5
A0A6H2NLB3	Phycocyanin subunit alpha	*Leptolyngbya sp. LCM1.Bin17*	10	2	6
A0A6H2NIZ8	Allophycocyanin	*Leptolyngbya sp. LCM1.Bin17*	21	2	3
A0A0N7Z574	Allophycocyanin alpha chain	*Leptolyngbya sp. NIES-2104*	17	3	3
A0A0S3TXT1	Allophycocyanin alpha subunit	*Leptolyngbya sp. NIES-3755*	12	2	2
A0AA96WDM6	Allophycocyanin subunit beta	*Leptolyngbya sp. NK1-12*	19	2	2
A0AA96WKI9	Allophycocyanin	*Leptolyngbya sp. NK1-12*	12	2	2
A0AAT8XUK1	C-phycocyanin alpha chain	*Leptolyngbya sp. O-77*	23	2	2
K9Q0W1	Allophycocyanin, beta subunit	*Leptolyngbya sp. PCC 7376*	22	2	3
A0A969AXA5	Allophycocyanin	*Leptolyngbya sp. RL_3_1*	12	3	3
A0A6G3YUP6	Allophycocyanin	*Leptolyngbya sp. SIO1D8*	12	2	2
A0A6I5RCW1	Allophycocyanin	*Leptolyngbya sp. SIO1E4*	19	4	4
A0A6I5NF67	Allophycocyanin subunit beta	*Leptolyngbya sp. SIO4C1*	19	2	5
A0A6I5N4Q9	Phycocyanin subunit beta	*Leptolyngbya sp. SIO4C1*	12	2	2
A0A6I5NSQ1	Phycocyanin subunit alpha	*Leptolyngbya sp. SIOISBB*	10	2	6
A0A6I5NL99	Allophycocyanin	*Leptolyngbya sp. SIOISBB*	20	2	3
Leptolyngbyaceae cyanobacterium					
A0A969RT93	Allophycocyanin	*Leptolyngbyaceae cyanobacterium CRU_2_3*	11	2	2
A0A969UNB4	Allophycocyanin	*Leptolyngbyaceae cyanobacterium CSU_1_3*	11	2	2
A0A969QMQ5	Allophycocyanin subunit beta	*Leptolyngbyaceae cyanobacterium CSU_1_4*	30	3	4
A0A925BUN4	Allophycocyanin	*Leptolyngbyaceae cyanobacterium LF-bin-113*	11	2	2
A0A978TH61	Allophycocyanin subunit beta	*Leptolyngbyaceae cyanobacterium M65_K2018_010*	19	2	5
A0A978TH62	Allophycocyanin subunit alpha	*Leptolyngbyaceae cyanobacterium M65_K2018_010*	21	3	4
A0A968ZW78	Allophycocyanin subunit beta	*Leptolyngbyaceae cyanobacterium RM1_1_2*	19	2	5
A0A968ZUC0	Allophycocyanin	*Leptolyngbyaceae cyanobacterium RM1_1_2*	12	2	2
A0A9E5RLI8	Allophycocyanin subunit beta	*Leptolyngbyaceae cyanobacterium RM1_406_9*	19	2	5
A0A9E5UWK1	Phycocyanin subunit beta	*Leptolyngbyaceae cyanobacterium RM2_2_4*	12	2	2
A0A969TMT1	Allophycocyanin	*Leptolyngbyaceae cyanobacterium RU_5_1*	11	2	2
A0A969P8E8	Phycocyanin subunit alpha	*Leptolyngbyaceae cyanobacterium RU_5_1*	19	3	7
A0A969GW41	Allophycocyanin subunit beta	*Leptolyngbyaceae cyanobacterium SL_5_14*	19	2	5
A0A968NRG9	Allophycocyanin	*Leptolyngbyaceae cyanobacterium SM1_3_5*	12	2	2
A0A968U328	Allophycocyanin	*Leptolyngbyaceae cyanobacterium SM1_4_3*	19	3	3
A0A968PWS3	Phycocyanin subunit beta	*Leptolyngbyaceae cyanobacterium SM1_4_3*	12	2	2
A0A968QC93	Allophycocyanin subunit beta	*Leptolyngbyaceae cyanobacterium SM2_5_2*	19	2	5
A0A968QBT3	Allophycocyanin	*Leptolyngbyaceae cyanobacterium SM2_5_2*	21	3	4
A0A968UCT1	Phycocyanin subunit alpha	*Leptolyngbyaceae cyanobacterium SM2_5_2*	10	2	6
A0A930SQK6	Allophycocyanin subunit alpha	*Leptolyngbyaceae cyanobacterium T60_A2020_046*	12	2	2
Limnofasciculus baicalensis					
A0AAE3GSN4	Allophycocyanin subunit beta	*Limnofasciculus baicalensis BBK-W-15*	24	3	6
A0AAE3GU58	Allophycocyanin	*Limnofasciculus baicalensis BBK-W-15*	12	3	3
Limnoraphis robusta					
A0A0F5YL91	Allophycocyanin	*Limnoraphis robusta CS-951*	27	4	4
Limnoraphis sp.					
A0A9E4JMK2	Allophycocyanin subunit beta	*Limnoraphis sp. WC205*	22	2	6

A0A9E4JH66	Allophycocyanin subunit alpha	*Limnoraphis sp. WC205*	20	3	3
Limnothrix rosea					
A0A1Q4R179	Allophycocyanin subunit beta	*Limnothrix rosea IAM M-220*	22	2	3
A0A1Q4R1B7	Allophycocyanin	*Limnothrix rosea IAM M-220*	27	3	4
Limnothrix sp.					
A0A1C0VDV2	Allophycocyanin subunit beta	*Limnothrix sp. P13C2*	22	2	6
A0A969KEN0	Allophycocyanin	*Limnothrix sp. RL_2_0*	21	3	4
A0A969KEW9	Allophycocyanin subunit beta	*Limnothrix sp. RL_2_0*	22	2	3
Lyngbya aestuarii					
U7QGP0	Allophycocyanin, beta subunit	*Lyngbya aestuarii BL J*	22	2	6
U7QGG4	Allophycocyanin alpha chain	*Lyngbya aestuarii BL J*	13	3	3
Lyngbya sp.					
A0YY95	Allophycocyanin beta subunit	*Lyngbya sp. (strain PCC 8106)*	22	2	6
Mastigocladus laminosus					
P00315	Allophycocyanin alpha chain	*Mastigocladus laminosus*	33	5	5
Merismopedia glauca					
A0A2T1BZ20	Allophycocyanin subunit beta	*Merismopedia glauca CCAP 1448/3*	30	3	7
A0A2T1BZC4	Allophycocyanin	*Merismopedia glauca CCAP 1448/3*	12	2	2
Merismopedia sp.					
A0A6P0YT94	Phycocyanin subunit alpha	*Merismopedia sp. SIO2A8*	10	2	6
A0A6P0Z1J5	Allophycocyanin	*Merismopedia sp. SIO2A8*	12	2	2
Microcoleaceae cyanobacterium					
A0A355CEP6	Allophycocyanin	*Microcoleaceae cyanobacterium UBA10368*	19	4	4
A0A349JQC7	Allophycocyanin	*Microcoleaceae cyanobacterium UBA11344*	19	4	4
Microcoleus sp.					
A0A970AMN8	Allophycocyanin	*Microcoleus sp. CSU_2_2*	17	3	3
A0A926YRL8	Allophycocyanin subunit beta	*Microcoleus sp. FACHB-1515*	19	2	5
A0A926TZ30	Allophycocyanin	*Microcoleus sp. FACHB-1515*	11	2	2
A0A926YAQ0	Allophycocyanin	*Microcoleus sp. FACHB-68*	17	3	3
A0A926Y8G0	Allophycocyanin subunit beta	*Microcoleus sp. FACHB-831*	19	3	3
A0A926Y7Z8	Allophycocyanin	*Microcoleus sp. FACHB-831*	12	2	3
A0A926S9B4	Allophycocyanin subunit beta	*Microcoleus sp. FACHB-SPT15*	19	2	5
A0A964FY72	Allophycocyanin subunit alpha	*Microcoleus sp. PH2017_37_MFU_D_B*	7	2	2
Microcystis aeruginosa					
A0A510PK91	Phycocyanin subunit beta	*Microcystis aeruginosa 11-30S32*	33	3	6
A0A841ULE8	Allophycocyanin subunit beta	*Microcystis aeruginosa BLCC-F108*	44	5	9
A0A857CZT7	Allophycocyanin subunit beta	*Microcystis aeruginosa FD4*	44	5	6
A0A857D0R4	Phycocyanin subunit alpha	*Microcystis aeruginosa FD4*	27	4	8
A0A857D0J1	Phycocyanin subunit beta	*Microcystis aeruginosa FD4*	33	3	6
A0A966FZR1	Allophycocyanin subunit beta	*Microcystis aeruginosa G11-04*	44	5	9
A0A966L378	Phycocyanin subunit alpha	*Microcystis aeruginosa G11-04*	27	4	8
A0A1V4BS86	Phycocyanin subunit alpha	*Microcystis aeruginosa KW*	19	3	7
A0A552EV89	Phycocyanin subunit beta	*Microcystis aeruginosa Ma_MB_F_20061100_S20D*	33	3	6
A0A552EXM2	Allophycocyanin subunit beta	*Microcystis aeruginosa Ma_MB_S_20031200_S102*	44	5	9
A0A552E7C2	Phycocyanin subunit beta	*Microcystis aeruginosa Ma_MB_S_20031200_S102*	33	3	6

A0A552AN53	Allophycocyanin subunit beta	*Microcystis aeruginosa Ma_QC_H_19870700_S124*	44	5	6
A0A552DNI5	Phycocyanin subunit alpha	*Microcystis aeruginosa Ma_QC_B_20070730_S2*	28	4	8
A0A551YMG3	Phycocyanin subunit alpha	*Microcystis aeruginosa Ma_QC_C_20070703_M131*	28	4	8
A0A552FHF8	Allophycocyanin	*Microcystis aeruginosa Ma_QC_Ca_00000000_S207*	34	4	6
A0A552G5E2	Allophycocyanin subunit beta	*Microcystis aeruginosa Ma_QC_Ch_20071001_S25D*	44	5	6
A0A552G5E8	Allophycocyanin	*Microcystis aeruginosa Ma_QC_Ch_20071001_S25D*	35	5	7
A0A5A5RV58	C-phycocyanin alpha chain	*Microcystis aeruginosa NIES-2520*	27	3	4
A0A2H6BXJ4	Phycocyanin alpha subunit	*Microcystis aeruginosa NIES-298*	18	2	3
A0A6H9GGD1	Phycocyanin alpha subunit	*Microcystis aeruginosa NIES-3787*	27	4	8
A0A6H9FZU6	Phycocyanin alpha subunit	*Microcystis aeruginosa NIES-3804*	36	4	7
A0A6H9GLU2	Phycocyanin beta subunit	*Microcystis aeruginosa NIES-3804*	33	3	6
A0A0A1W052	Phycocyanin alpha chain	*Microcystis aeruginosa NIES-44*	27	4	8
A0A822LDG6	C-phycocyanin alpha chain	*Microcystis aeruginosa PCC 9432*	28	4	8
I4G9Z4	C-phycocyanin alpha chain	*Microcystis aeruginosa PCC 9443*	27	3	6
I4HAS2	C-phycocyanin beta chain	*Microcystis aeruginosa PCC 9807*	33	3	6
S3KI04	Allophycocyanin beta chain	*Microcystis aeruginosa SPC777*	44	5	6
Microcystis flos-aquae					
A0A552KUX6	Phycocyanin subunit alpha	*Microcystis flos-aquae Mf_QC_C_20070823_S10D*	36	5	9
A0A3E0KYJ4	Phycocyanin subunit beta	*Microcystis flos-aquae TF09*	37	4	7
Microcystis novacekii					
A0A552IP97	Phycocyanin subunit alpha	*Microcystis novacekii Mn_MB_F_20050700_S1D*	28	4	8
Microcystis sp.					
A0A2L2XPC6	Phycocyanin alpha chain	*Microcystis sp. 0824*	26	3	3
A0A2L2XUH8	Phycocyanin beta chain	*Microcystis sp. 0824*	33	3	6
A0A552ARD9	Allophycocyanin	*Microcystis sp. M_OC_Ca_00000000_C217Col*	35	4	6
A0A2P1UGR1	Phycocyanin subunit beta	*Microcystis sp. MC19*	33	3	6
Microcystis viridis					
A0A3G9JUA6	Allophycocyanin alpha subunit	*Microcystis viridis NIES-102*	42	5	7
A0A3G9JQA9	Phycocyanin alpha subunit	*Microcystis viridis NIES-102*	18	2	3
Microcystis wesenbergii					
A0A552LTQ6	Phycocyanin subunit alpha	*Microcystis wesenbergii Mw_MB_S_20031200_S109D*	27	4	8
A0A552LTQ4	Phycocyanin subunit beta	*Microcystis wesenbergii Mw_MB_S_20031200_S109D*	20	2	3
Microseira wollei					
A0AAV3X7D5	Allophycocyanin alpha subunit	*Microseira wollei NIES-4236*	12	2	2
Mojavia pulchra					
A0A951UFQ2	Phycocyanin subunit alpha	*Mojavia pulchra JT2-VF2*	23	2	2
A0A951UF51	Allophycocyanin subunit alpha	*Mojavia pulchra JT2-VF2*	13	3	3
A0A951UFB7	Phycocyanin subunit beta	*Mojavia pulchra JT2-VF2*	12	2	2
Neosynechococcus sphagnicola					
A0A098TLR2	Allophycocyanin	*Neosynechococcus sphagnicola sy1*	13	3	3
Nodosilinea sp.					
A0AA97EBH8	Allophycocyanin	*Nodosilinea sp. E11*	21	3	4
A0A8J7EHH2	Allophycocyanin subunit alpha	*Nodosilinea sp. LEGE 06152*	20	2	3

A0A8J7E7L2	Allophycocyanin subunit beta	*Nodosilinea sp. LEGE 07088*	24	3	6
Nodularia harveyana					
A0A8J9TPA9	Allophycocyanin alpha chain	*Nodularia harveyana CCAP 1452/1*	25	3	3
Nodularia sp.					
A0A6P1ZYF1	Allophycocyanin	*Nodularia sp.*	26	3	3
A0A218Q2C8	Phycocyanin	*Nodularia sp. NIES-3585*	25	3	3
Nodularia spumigena					
A0A166K5J4	Allophycocyanin	*Nodularia spumigena CENA596*	25	3	3
Nostoc azollae					
D7DY32	Phycocyanin	*Nostoc azollae (strain 0708)*	20	2	3
D7DY33	Allophycocyanin, beta subunit	*Nostoc azollae (strain 0708)*	24	3	7
D7E337	Phycocyanin, alpha subunit	*Nostoc azollae (strain 0708)*	19	3	6
D7E338	Phycocyanin, beta subunit	*Nostoc azollae (strain 0708)*	13	2	2
Nostoc cycadae					
A0A2H6LMW1	Allophycocyanin subunit alpha apoprotein	*Nostoc cycadae WK-1*	19	3	3
Nostoc edaphicum					
A0A7D7QL00	Allophycocyanin	*Nostoc edaphicum CCNP1411*	19	3	4
Nostoc minutum					
A0A367RWU6	Allophycocyanin	*Nostoc minutum NIES-26*	27	4	4
Nostoc piscinale					
A0A0M4TKC4	Phycocyanin	*Nostoc piscinale CENA21*	23	2	2
Nostoc sp.					
K9QZL0	Allophycocyanin alpha subunit apoprotein	*Nostoc sp. (strain ATCC 29411 / PCC 7524)*	30	3	5
K9R0X7	Allophycocyanin beta subunit apoprotein	*Nostoc sp. (strain ATCC 29411 / PCC 7524)*	24	3	7
A0A252DGX6	Allophycocyanin	*Nostoc sp. 106C*	12	2	2
A0A367PVT9	Allophycocyanin	*Nostoc sp. ATCC 43529*	19	4	5
A0A2I8A176	Allophycocyanin	*Nostoc sp. CENA543*	32	4	4
A0A2I8AB13	Phycocyanin subunit alpha	*Nostoc sp. CENA543*	18	2	3
A0A926VVH8	Allophycocyanin	*Nostoc sp. FACHB-888*	12	2	3
A0A9D7VE10	Allophycocyanin	*Nostoc sp. GBBB01*	12	2	3
A0A1E2WL85	Allophycocyanin	*Nostoc sp. KVJ20*	12	2	3
A0A2L2NUU7	Allophycocyanin subunit alpha	*Nostoc sp. 'Lobaria pulmonaria (5183) cyanobiont'*	19	3	4
A0A939IF90	Allophycocyanin	*Nostoc sp. LPT*	19	4	5
A0A1C0VME8	Allophycocyanin subunit beta	*Nostoc sp. MBR 210*	24	3	7
A0A1C0VMD8	Allophycocyanin	*Nostoc sp. MBR 210*	43	6	8
A0A1Z4IDX0	Phycobilisome protein	*Nostoc sp. NIES-2111*	17	3	3
A0A2D0HKN0	Allophycocyanin	*Nostoc sp. 'Peltigera malacea cyanobiont' DB3992*	12	2	3
A0A235IB78	Allophycocyanin	*Nostoc sp. 'Peltigera membranacea cyanobiont' 210A*	17	3	4
A0A235IB31	Phycocyanin subunit alpha	*Nostoc sp. 'Peltigera membranacea cyanobiont' 210A*	10	2	6
A0A235J6M6	Allophycocyanin	*Nostoc sp. 'Peltigera membranacea cyanobiont' 232*	17	3	4
A0A2L2NAQ3	Allophycocyanin subunit alpha	*Nostoc sp. 'Peltigera membranacea cyanobiont' N6*	17	3	4
A0A252E112	Allophycocyanin	*Nostoc sp. T09*	12	2	2
A0A841VJM5	Allophycocyanin	*Nostoc sp. UCD120*	19	3	4
A0A4Y5PW22	Allophycocyanin alpha	*Nostoc sp. WR13*	43	6	8

A0A4Y5PW23	Allophycocyanin beta	*Nostoc sp. WR13*	24	3	7
Nostoc sphaeroide					
A0A5P8W6Q0	ApcA, allophycocyanin alpha subunit	*Nostoc sphaeroides CCNUC1*	17	3	4
Nostocaceae cyanobacterium					
A0A838VMI0	Allophycocyanin subunit beta	*Nostocaceae cyanobacterium*	19	2	6
A0A838VMA7	Phycocyanin subunit beta	*Nostocaceae cyanobacterium*	12	2	2
Nostocales cyanobacterium					
A0A9E6RYN0	Allophycocyanin	*Nostocales cyanobacterium*	30	3	5
A0A9E6UT21	Phycocyanin subunit alpha	*Nostocales cyanobacterium*	23	2	2
A0A9E6RXB7	Allophycocyanin subunit beta	*Nostocales cyanobacterium*	16	2	5
A0A9E6V111	Phycocyanin subunit beta	*Nostocales cyanobacterium*	16	2	2
A0A1Y0RS29	Allophycocyanin	*Nostocales cyanobacterium HT-58-2*	19	3	3
A0A1Y0REZ5	Phycocyanin subunit alpha	*Nostocales cyanobacterium HT-58-2*	23	2	2
A0A928WCI3	Allophycocyanin subunit alpha	*Nostocales cyanobacterium LEGE 11386*	26	3	3
A0A928WPS0	Phycocyanin subunit alpha	*Nostocales cyanobacterium LEGE 11386*	17	2	2
Oculatella sp.					
A0A8J7JSZ9	Allophycocyanin subunit beta	*Oculatella sp. LEGE 06141*	24	3	6
A0A8J7JX08	Phycocyanin subunit alpha	*Oculatella sp. LEGE 06141*	10	2	5
Oscillatoria acuminata					
K9TDG1	Allophycocyanin alpha subunit apoprotein	*Oscillatoria acuminata PCC 6304*	20	2	4
Oscillatoria nigro-viridis					
A0A8J9XFI1	Allophycocyanin alpha chain	*Oscillatoria nigro-viridis*	32	4	6
Oscillatoria sp.					
A0A926X3L2	Allophycocyanin	*Oscillatoria sp. FACHB-1407*	20	2	3
A0A926X6J0	Phycocyanin subunit alpha	*Oscillatoria sp. FACHB-1407*	17	2	3
Oscillatoriaceae cyanobacterium					
A0A978S9U2	Allophycocyanin subunit alpha	*Oscillatoriaceae cyanobacterium M33_DOE_052*	21	2	4
A0A978STU2	Allophycocyanin subunit alpha	*Oscillatoriaceae cyanobacterium M7585_C2015_266*	7	2	2
Oscillatoriales cyanobacterium					
A0A976CFJ9	Allophycocyanin	*Oscillatoriales cyanobacterium*	7	2	2
A0A1J5GIY8	Allophycocyanin subunit beta	*Oscillatoriales cyanobacterium CG2_30_44_21*	27	3	7
A0A1J5GL20	Allophycocyanin	*Oscillatoriales cyanobacterium CG2_30_44_21*	19	4	4
A0A969MZ16	Allophycocyanin	*Oscillatoriales cyanobacterium RM1_1_9*	7	2	2
A0A968Y292	Allophycocyanin	*Oscillatoriales cyanobacterium RU_3_3*	12	3	3
A0A968IZQ6	Allophycocyanin	*Oscillatoriales cyanobacterium SM2_1_8*	12	2	2
A0A968M204	Phycocyanin subunit alpha	*Oscillatoriales cyanobacterium SM2_1_8*	10	2	6
A0A968HQM9	Allophycocyanin subunit beta	*Oscillatoriales cyanobacterium SM2_2_1*	17	2	3
A0A7C3PFJ8	Allophycocyanin	*Oscillatoriales cyanobacterium SpSt-418*	12	3	3
A0A1C0VYM6	Allophycocyanin	*Oscillatoriales cyanobacterium USR001*	19	3	3
Pannus brasiliensis					
A0AAW9QT48	Allophycocyanin subunit alpha	*Pannus brasiliensis CCIBt3594*	26	3	4
A0AAW9QFW6	Allophycocyanin subunit beta	*Pannus brasiliensis CCIBt3594*	30	3	4
Pantanalinema sp.					
A0A937JZ07	Phycocyanin subunit alpha	*Pantanalinema sp. GBBB05*	19	2	5
A0A937EXK1	Allophycocyanin	*Pantanalinema sp. GBBB05*	12	2	2

Parathermosynechococcus lividus					
A0A2D2Q321	Allophycocyanin	*Parathermosynechococcus lividus PCC 6715*	12	2	2
A0A2D2Q3N1	C-phycocyanin beta subunit	*Parathermosynechococcus lividus PCC 6715*	12	2	2
Pegethrix bostrychoides					
A0A951U6B3	Phycocyanin subunit alpha	*Pegethrix bostrychoides GSE-TBD4-15B*	19	3	7
A0A951U519	Allophycocyanin subunit alpha	*Pegethrix bostrychoides GSE-TBD4-15B*	12	3	3
Pelatocladus maniniholoensis					
A0A9E3HAM7	Allophycocyanin subunit alpha	*Pelatocladus maniniholoensis HA4357-MV3*	19	4	5
Phormidium ambiguum					
A0A1U7IEE7	Allophycocyanin subunit beta	*Phormidium ambiguum IAM M-71*	19	2	5
A0A1U7IE87	Allophycocyanin	*Phormidium ambiguum IAM M-71*	17	3	3
Phormidium sp.					
A0A522XEV4	Allophycocyanin	*Phormidium sp. SL48-SHIP*	13	3	3
Phormidium tenue					
A0A1U7JAT3	Allophycocyanin	*Phormidium tenue NIES-30*	27	4	5
Picosynechococcus sp.					
A0AAE8LPP7	Allophycocyanin alpha subunit apoprotein	*Picosynechococcus sp. OG1*	20	2	3
Planktothricoides raciborskii					
A0AAU8JH72	Allophycocyanin subunit beta	*Planktothricoides raciborskii GIHE-MW2*	25	3	5
Planktothricoides sp.					
A0A7C3VLG6	Allophycocyanin subunit beta	*Planktothricoides sp. SpSt-374*	19	2	4
A0A0M1JPI9	Allophycocyanin	*Planktothricoides sp. SR001*	12	2	2
Planktothrix agardhii					
A0A4P5ZCB3	Allophycocyanin alpha subunit	*Planktothrix agardhii CCAP 1459/11A*	13	3	3
Planktothrix paucivesiculata					
A0A7Z9DWQ8	Allophycocyanin alpha chain	*Planktothrix paucivesiculata PCC 9631*	13	2	2
A0A7Z9BJ54	Allophycocyanin beta chain	*Planktothrix paucivesiculata PCC 9631*	30	3	4
Planktothrix pseudagardhii					
A0A9W4G280	Allophycocyanin alpha chain	*Planktothrix pseudagardhii*	32	5	5
Planktothrix serta					
A0A7Z9BR97	Allophycocyanin beta chain	*Planktothrix serta PCC 8927*	30	3	7
A0A7Z9BR96	Allophycocyanin alpha chain	*Planktothrix serta PCC 8927*	25	3	3
Planktothrix sp.					
A0A354WH48	Allophycocyanin	*Planktothrix sp. UBA10369*	12	2	2
A0A3C1RUJ6	Allophycocyanin subunit beta	*Planktothrix sp. UBA8407*	30	3	4
Planktothrix tepida					
A0A1J1LN43	Allophycocyanin beta chain	*Planktothrix tepida PCC 9214*	30	3	7
Plectolyngbya sp.					
A0A951NWS1	Allophycocyanin subunit alpha	*Plectolyngbya sp. WJT66-NPBG17*	12	3	3
Plectonema cf. radiosum					
A0A8J7K299	Phycocyanin subunit alpha	*Plectonema cf. radiosum LEGE 06105*	19	3	6
Pleurocapsa sp.					
A0A969NNN1	Allophycocyanin subunit beta	*Pleurocapsa sp. CRU_1_2*	19	2	3
K9TAS6	Allophycocyanin alpha subunit apoprotein	*Pleurocapsa sp. PCC 7327*	35	4	6
A0A968IRB4	Allophycocyanin	*Pleurocapsa sp. SU_5_0*	17	3	3

Pleurocapsales cyanobacterium					
A0A929FTT8	Allophycocyanin subunit beta	*Pleurocapsales cyanobacterium LEGE 06147*	30	3	4
A0A929FWD4	Allophycocyanin	*Pleurocapsales cyanobacterium LEGE 06147*	20	2	3
A0A928ZDL8	Allophycocyanin subunit beta	*Pleurocapsales cyanobacterium LEGE 10410*	19	2	3
Pseudanabaena catenata					
A0A9X4RIW4	Allophycocyanin subunit beta	*Pseudanabaena catenata USMAC16*	17	2	3
Pseudanabaena cinerea					
A0A926UST9	Allophycocyanin subunit beta	*Pseudanabaena cinerea FACHB-1277*	27	3	7
A0A926UTP6	Allophycocyanin	*Pseudanabaena cinerea FACHB-1277*	11	2	2
Pseudanabaena frigida					
A0A2W4W8I7	Allophycocyanin subunit beta	*Pseudanabaena frigida*	27	3	7
A0A2W4Y8U6	Allophycocyanin	*Pseudanabaena frigida*	19	4	4
Pseudanabaena sp					
A0A2W7AAG3	Allophycocyanin subunit beta	*Pseudanabaena sp*	22	2	6
A0A2W6ZMB9	Allophycocyanin subunit beta	*Pseudanabaena sp*	27	3	7
A0A352JCX6	Allophycocyanin	*Pseudanabaena sp*	12	2	2
A0A2W6Z5S6	Allophycocyanin	*Pseudanabaena sp*	19	4	4
A0A2W7C4P5	Allophycocyanin	*Pseudanabaena sp*	19	3	3
A0A2Z5X2N9	Allophycocyanin alpha subunit	*Pseudanabaena sp. ABRG5-3*	19	4	4
A0A970B1A0	Phycocyanin subunit alpha	*Pseudanabaena sp. CRU_2_10*	10	2	6
A0A926WAZ3	Allophycocyanin subunit beta	*Pseudanabaena sp. FACHB-2040*	19	2	5
V5NXK8	ApcB	*Pseudanabaena sp. lw0831*	22	2	6
K9SNR5	Allophycocyanin beta subunit apoprotein	*Pseudanabaena sp. PCC 7367*	35	4	8
A0A256B7Q9	Allophycocyanin subunit beta	*Pseudanabaena sp. SR411*	27	3	7
A0A968Q0N3	Allophycocyanin subunit beta	*Pseudanabaena sp. SU_2_4*	25	3	4
A0A6C2DZJ7	Allophycocyanin	*Pseudanabaena sp. UWO310*	12	2	2
A0A652Z030	Allophycocyanin subunit beta	*Pseudanabaena sp. UWO311*	22	2	6
A0A652Z043	Allophycocyanin	*Pseudanabaena sp. UWO311*	19	4	4
Pseudanabaenaceae cyanobacterium					
A0A928VES5	Allophycocyanin subunit alpha	*Pseudanabaenaceae cyanobacterium LEGE 13415*	19	4	4
Pseudocalidococcus azoricus					
A0AAE4FQ17	Allophycocyanin subunit alpha	*Pseudocalidococcus azoricus BACA0444*	12	2	2
A0AAE4FUT5	Phycocyanin subunit beta	*Pseudocalidococcus azoricus BACA0444*	12	2	2
Richelia sinica					
A0A975T7D3	C-phycocyanin alpha chain	*Richelia sinica FACHB-800*	26	2	2
Richelia sp					
A0A3B9F727	Allophycocyanin	*Richelia sp*	13	2	2
A0A969QCR1	Allophycocyanin	*Richelia sp. CSU_2_1*	17	3	3
A0A969AE68	Allophycocyanin subunit beta	*Richelia sp. RM1_1_1*	19	2	6
A0A969MJ06	Allophycocyanin	*Richelia sp. RM2_1_2*	25	3	3
A0A968WAI6	Phycocyanin subunit alpha	*Richelia sp. SM1_7_0*	19	3	6
Rippkaea orientalis					
B7K5Q4	Phycobilisome protein	*Rippkaea orientalis (strain PCC 8801 / RF-1)*	11	2	2
B7K5Q5	Allophycocyanin, beta subunit	*Rippkaea orientalis (strain PCC 8801 / RF-1)*	30	3	7
B7JX68	Phycocyanin, alpha subunit	*Rippkaea orientalis (strain PCC 8801 / RF-1)*	10	2	6

Rivularia sp.					
A0A949THS9	Allophycocyanin subunit beta	*Rivularia sp.*	19	2	6
A0A949TLI6	Phycocyanin subunit alpha	*Rivularia sp.*	19	3	6
A0A949T6V0	Allophycocyanin subunit alpha	*Rivularia sp. MS3*	31	4	4
K9RFJ2	Allophycocyanin beta subunit apoprotein	*Rivularia sp. PCC 7116*	19	2	6
K9REV4	Phycocyanin, alpha subunit	*Rivularia sp. PCC 7116*	19	3	6
Scytonema hofmannii					
A0A139WXF7	Allophycocyanin	*Scytonema hofmannii PCC 7110*	32	4	4
Scytonema sp.					
A0A969SD13	Allophycocyanin subunit beta	*Scytonema sp. CRU_2_7*	14	2	3
A0AA91GVQ4	Allophycocyanin	*Scytonema sp. HK-05*	19	4	5
A0A969CK74	Allophycocyanin	*Scytonema sp. RU_4_4*	19	2	2
A0A968YRH4	Phycocyanin subunit alpha	*Scytonema sp. RU_4_4*	19	3	7
A0A969CLK2	Phycocyanin subunit alpha	*Scytonema sp. RU_4_4*	10	2	6
A0A844MPL2	Allophycocyanin	*Scytonema sp. UIC 10036*	19	2	2
Snowella sp					
A0A2W7BUA5	Allophycocyanin subunit beta	*Snowella sp*	22	2	3
A0A2W7BC75	Allophycocyanin	*Snowella sp*	19	3	3
Sphaerospermopsis reniformis					
A0A479ZWB6	Phycocyanin subunit alpha	*Sphaerospermopsis reniformis*	34	4	7
A0A480A8R2	Phycocyanin	*Sphaerospermopsis reniformis*	22	3	5
A0A479ZSX1	Phycocyanin, beta subunit	*Sphaerospermopsis reniformis*	21	4	6
Sphaerospermopsis sp.					
A0A846EIF2	Allophycocyanin	*Sphaerospermopsis sp. SIO1G1*	19	2	2
A0A846EIS8	Phycocyanin subunit alpha	*Sphaerospermopsis sp. SIO1G1*	23	2	2
A0A846EI73	Phycocyanin subunit beta	*Sphaerospermopsis sp. SIO1G1*	20	3	3
Spirulina major					
A0A8J9SZF1	Allophycocyanin beta chain	*Spirulina major*	19	2	2
Spirulinaceae cyanobacterium					
A0A969I468	Allophycocyanin	*Spirulinaceae cyanobacterium RM2_2_10*	21	3	4
A0A968T7X6	Allophycocyanin subunit beta	*Spirulinaceae cyanobacterium SM2_1_0*	28	3	5
Stanieria cyanosphaera					
K9XS05	Allophycocyanin beta subunit apoprotein	*Stanieria cyanosphaera (strain ATCC 29371 / PCC 7437)*	30	3	4
Stanieria sp.					
A0A140KB32	Phycobilisome protein	*Stanieria sp. NIES-3757*	20	2	3
Stenomitos frigidus					
A0A2T1DX81	Allophycocyanin	*Stenomitos frigidus ULC18*	12	2	2
Symplocastrum torsivum					
A0A951UD14	Allophycocyanin subunit beta	*Symplocastrum torsivum CPER-KK1*	19	2	5
Synechococcaceae bacterium					
A0A966T0S8	Phycocyanin subunit alpha	*Synechococcaceae bacterium WBB_3_034*	10	2	6
Synechococcaceae cyanobacterium					
A0A969KH47	Allophycocyanin	*Synechococcaceae cyanobacterium RL_1_2*	7	2	2
A0A969KIN5	Allophycocyanin subunit beta	*Synechococcaceae cyanobacterium RL_1_2*	22	2	6
A0A969NBQ1	Allophycocyanin	*Synechococcaceae cyanobacterium RM1_1_27*	7	2	2

A0A930TJP2	Phycocyanin subunit alpha	*Synechococcales cyanobacterium C42_A2020_086*	23	2	2
A0A930XIE9	Allophycocyanin subunit alpha	*Synechococcales cyanobacterium C42_A2020_086*	12	2	2
A0A966B0S7	Phycocyanin subunit alpha	*Synechococcales cyanobacterium H12SWP_bin.12*	10	2	6
A0A930U2A6	Allophycocyanin subunit alpha	*Synechococcales cyanobacterium K44_A2020_017*	12	2	2
A0A978U6M1	Allophycocyanin subunit beta	*Synechococcales cyanobacterium M55_K2018_004*	25	3	4
A0A978U294	Phycocyanin subunit alpha	*Synechococcales cyanobacterium M55_K2018_004*	19	2	5
A0A978U6M2	Allophycocyanin subunit alpha	*Synechococcales cyanobacterium M55_K2018_004*	12	3	3
A0A969DZ07	Phycocyanin subunit alpha	*Synechococcales cyanobacterium RM1_1_8*	10	2	6
A0A969DTV0	Allophycocyanin	*Synechococcales cyanobacterium RM1_1_8*	7	2	2
A0A969DSH7	Allophycocyanin subunit beta	*Synechococcales cyanobacterium RM1_1_8*	19	2	4
A0A930TT59	Allophycocyanin subunit beta	*Synechococcales cyanobacterium T60_A2020_003*	30	3	7
A0A930TPG9	Allophycocyanin subunit alpha	*Synechococcales cyanobacterium T60_A2020_003*	27	3	5
Synechococcus lacustris					
A0A2P7EIB9	Phycocyanin subunit beta	*Synechococcus lacustris str. Tous*	12	2	2
Synechococcus sp					
Q76N39	Alpha-phycocyanin	*Synechococcus sp*	17	2	3
K9RWE6	Phycocyanin, beta subunit	*Synechococcus sp. (strain ATCC 27167 / PCC 6312)*	12	2	2
A0A2G8PFN9	Allophycocyanin subunit alpha	*Synechococcus sp. 60AY4M2*	13	3	3
Q05ZB1	Phycocyanin, alpha subunit	*Synechococcus sp. BL107*	10	2	6
A0A937HWM7	Phycocyanin subunit alpha	*Synechococcus sp. BS307-5m-G38*	10	2	6
A0A1Q2TZY0	Allophycocyanin, beta subunit	*Synechococcus sp. NIES-970*	22	2	3
A0A1Q2U063	Allophycocyanin alpha subunit	*Synechococcus sp. NIES-970*	27	4	5
A0A1L6BXH6	Allophycocyanin subunit alpha (Fragment)	*Synechococcus sp. OH20*	13	3	3
A0A1L6BXH8	Allophycocyanin subunit alpha (Fragment)	*Synechococcus sp. OH28*	13	3	3
K9SUP9	Allophycocyanin beta subunit apoprotein	*Synechococcus sp. PCC 7502*	17	2	3
A0A1J0PDQ2	Phycocyanin subunit beta	*Synechococcus sp. SynAce01*	12	2	2
A0A024CHA8	R-phycocyanin II, alpha subunit	*uncultured Synechococcus sp*	10	2	6
A0A024CH50	R-phycocyanin II, alpha subunit	*uncultured Synechococcus sp*	10	2	6
A0A024CHA6	R-phycocyanin II, alpha subunit	*uncultured Synechococcus sp*	10	2	6
Synechocystis sp.					
Q01951	Allophycocyanin alpha chain	*Synechocystis sp. (strain ATCC 27184 / PCC 6803 / Kazusa)*	13	3	3
A0A068N0S6	Allophycocyanin beta chain	*Synechocystis sp. (strain PCC 6714)*	19	2	3
Q02923	Allophycocyanin alpha chain	*Synechocystis sp. (strain PCC 6714)*	12	2	2
Thermoleptolyngbya oregonensis					
A0AA97BC92	Allophycocyanin subunit beta	*Thermoleptolyngbya oregonensis NK1-22*	19	2	5
A0AA96Y7U5	Allophycocyanin	*Thermoleptolyngbya oregonensis NK1-22*	12	2	2
A0AA96Y2Z6	Phycocyanin subunit alpha	*Thermoleptolyngbya oregonensis NK1-22*	23	2	2
Thermosynechococcus sp					
A0A915V2U9	C-phycocyanin beta chain	*Thermosynechococcus sp*	12	2	2
A0A5C2M6M9	Allophycocyanin	*Thermosynechococcus sp. CL-1*	19	4	4
Thermosynechococcus vestitus					
P50030	Allophycocyanin alpha chain	*Thermosynechococcus vestitus (strain NIES-2133 / IAM M-273 / BP-1)*	19	4	4

Tolypothrix bouteillei					
A0A0C1QT48	Allophycocyanin	*Tolypothrix bouteillei VB521301*	32	5	5
Tolypothrix sp.					
A0A218QHG9	Phycobilisome protein	*Tolypothrix sp. NIES-4075*	25	3	4
A0A218QHD7	Allophycocyanin beta subunit	*Tolypothrix sp. NIES-4075*	14	2	3
A0A218QT84	Phycocyanin beta subunit	*Tolypothrix sp. NIES-4075*	16	3	5
A0A0D6KRK5	Allophycocyanin alpha subunit	*Tolypothrix sp. PCC 7601*	13	2	2
Trichormus variabilis					
Q3M9V1	Phycobilisome protein	*Trichormus variabilis (strain ATCC 29413 / PCC 7937)*	25	3	3
A0A3S1CEI7	Allophycocyanin alpha chain	*Trichormus variabilis SAG 1403-4b*	43	6	9
A0A433V1P0	Allophycocyanin beta chain	*Trichormus variabilis SAG 1403-4b*	24	3	7
Tumidithrix elongata					
A0AAW9Q8W0	Allophycocyanin	*Tumidithrix elongata BACA0141*	13	2	2
A0AAW9PVD1	Allophycocyanin subunit beta	*Tumidithrix elongata BACA0141*	16	2	5
Tychonema bourrellyi					
A0A2G4EVV9	Allophycocyanin	*Tychonema bourrellyi FEM_GT703*	19	4	4
Umezakia ovalisporum					
A0AA43GWQ4	Allophycocyanin subunit beta	*Umezakia ovalisporum FSS-62*	14	2	3
A0AA43GX24	Allophycocyanin subunit alpha	*Umezakia ovalisporum FSS-62*	26	4	4
A0AA43KFZ5	Phycocyanin subunit alpha	*Umezakia ovalisporum FSS-62*	17	2	2
Woronichinia naegeliana					
A0A977L2D6	Allophycocyanin	*Woronichinia naegeliana WA131*	12	3	3
A0A977PZ20	Allophycocyanin subunit beta	*Woronichinia naegeliana WA131*	19	2	3

Table 2: Proteins from cyanobacterial strains detected by trypsin digestion followed by LC-MS/MS analysis from lake water 2.

Accession	Description	Species	Coverage [%]	# Peptides	# PSMs
Aerosakkonema funiforme					
A0A926VEV9	Allophycocyanin	*Aerosakkonema funiforme FACHB-1375*	11	2	2
Aetokthonos hydrillicola					
A0AAP5I9Q5	Allophycocyanin subunit alpha	*Aetokthonos hydrillicola Thurmond2011*	17	3	3
aff. Roholtiella sp.					
A0A8J7D6K0	Allophycocyanin subunit alpha	*aff. Roholtiella sp. LEGE 12411*	17	3	3
Aliterella atlantica					
A0A0D8ZUK5	Allophycocyanin	*Aliterella atlantica CENA595*	17	3	3
Anabaena cylindrica					
P07325	Allophycocyanin alpha chain	*Anabaena cylindrica*	11	2	2
Anabaena sp.					
K7WMD9	Phycobilisome protein ApcA	*Anabaena sp. 90*	11	2	2
K7WTL4	Phycocyanin beta subunit	*Anabaena sp. 90*	39	4	4
A0A1B7V2J0	Phycocyanin	*Anabaena sp. AL09*	19	2	2
A0A1B7WMN4	Phycocyanin	*Anabaena sp. CRKS33*	34	3	3
A0A1B7WSR8	Allophycocyanin	*Anabaena sp. CRKS33*	11	2	2
A0A1B7WMQ4	Phycocyanin	*Anabaena sp. CRKS33*	19	2	2
A0A926ULC4	Allophycocyanin	*Anabaena sp. FACHB-1237*	11	2	2
A0A9X1GQJ4	Phycocyanin subunit alpha	*Anabaena sp. PCC 7938*	23	2	2
A0A9X1GP51	Allophycocyanin	*Anabaena sp. PCC 7938*	11	2	2
A0A9X1GNR2	Phycocyanin subunit beta	*Anabaena sp. PCC 7938*	19	2	2
A0A3D4C397	Phycocyanin subunit alpha	*Anabaena sp. UBA12330*	34	3	3
A0A1B7VSW7	Allophycocyanin	*Anabaena sp. WA113*	11	2	2
A0A455KZV8	Phycocyanin B	*uncultured Anabaena sp*	39	4	4
Anabaena sphaerica					
A0A927A4I2	Phycocyanin subunit alpha	*Anabaena sphaerica FACHB-251*	19	2	2
A0A927A3U9	Phycocyanin subunit beta	*Anabaena sphaerica FACHB-251*	31	3	4
Anabaenopsis circularis					
A0A1Z4GCJ2	Phycobilisome protein	*Anabaenopsis circularis NIES-21*	17	3	3
Anabaenopsis elenkinii					
A0A7S6TZF6	Allophycocyanin subunit alpha	*Anabaenopsis elenkinii CCIBt3563*	11	2	2
Anthocerotibacter panamensis					
A0AAJ6N6E0	ApcA2	*Anthocerotibacter panamensis*	11	2	2
Aphanizomenon flos-aquae					
A0A1B7WBM0	Phycocyanin	*Aphanizomenon flos-aquae WA102*	19	2	2
A0A1B7X4P2	Allophycocyanin	*Aphanizomenon flos-aquae WA102*	11	2	2
A0A1B7WZK6	Phycocyanin	*Aphanizomenon flos-aquae WA102*	31	3	3
Aphanizomenon sp.					
A0A844ICS2	Phycocyanin subunit beta	*Aphanizomenon sp. UHCC 0183*	52	6	7
Aphanothece hegewaldii					
A0A2T1LW89	Allophycocyanin	*Aphanothece hegewaldii CCALA 016*	12	2	2
Atlanticothrix silvestris					
A0A8J7H708	Allophycocyanin subunit alpha	*Atlanticothrix silvestris CENA357*	11	2	2

Calothrix sp.					
A0A0T7BMD2	Allophycocyanin	*Calothrix sp. 336/3*	12	2	2
A0A9E5V8C2	Allophycocyanin	*Calothrix sp. CSU_2_0*	12	2	2
A0A1Z4NJH9	Phycocyanin	*Calothrix sp. NIES-3974*	12	2	2
A0A1Z4R810	Phycobilisome protein	*Calothrix sp. NIES-4101*	17	3	3
Chlorogloea sp.					
A0A2T1EFR3	Allophycocyanin	*Chlorogloea sp. CCALA 695*	12	2	2
Chlorogloeopsis fritschii					
A0A3S0Y3A8	Allophycocyanin alpha chain	*Chlorogloeopsis fritschii PCC 6912*	11	2	2
A0A433NKR2	C-phycocyanin alpha chain	*Chlorogloeopsis fritschii PCC 6912*	23	2	2
A0A3S1AKY7	C-phycocyanin beta chain	*Chlorogloeopsis fritschii PCC 6912*	18	2	2
Chondrocystis sp.					
A0A1Z4RRS0	Phycobilisome protein	*Chondrocystis sp. NIES-4102*	23	4	4
Chroococcidiopsis sp.					
A0A2P8QIZ5	Allophycocyanin	*Chroococcidiopsis sp. CCALA 051*	17	3	3
A0A2S6VGG8	Allophycocyanin	*Chroococcidiopsis sp. TS-821*	17	3	3
Coleofasciculaceae cyanobacterium					
A0A968SN98	Allophycocyanin	*Coleofasciculaceae cyanobacterium SM2_1_6*	11	2	2
A0A968SF93	Phycocyanin subunit beta	*Coleofasciculaceae cyanobacterium SM2_1_6*	18	2	2
Coleofasciculus chthonoplastes					
B4VSN2	Phycobilisome protein	*Coleofasciculus chthonoplastes PCC 7420*	12	2	2
Coleofasciculus sp.					
A0A926XI84	Allophycocyanin	*Coleofasciculus sp. FACHB-SPT36*	11	2	2
A0A929AQ34	Allophycocyanin subunit alpha	*Coleofasciculus sp. LEGE 07092*	12	2	2
Cronbergia siamensis					
B5A5J8	Phycocyanin beta subunit	*Cronbergia siamensis TISTR 8012*	22	2	3
Cuspidothrix issatschenkoi					
A0A2S6CX50	Allophycocyanin	*Cuspidothrix issatschenkoi CHARLIE-1*	17	3	3
A0A2S6CQK6	Phycocyanin subunit alpha	*Cuspidothrix issatschenkoi CHARLIE-1*	23	2	2
A0A2S6CQK9	Phycocyanin subunit beta	*Cuspidothrix issatschenkoi CHARLIE-1*	39	4	4
Cyanobacteria bacterium					
A0A926PQ48	Allophycocyanin	*Cyanobacteria bacterium FACHB-DQ100*	11	2	2
A0A3M1PIT8	Phycocyanin subunit alpha	*Cyanobacteria bacterium J069*	23	2	2
A0A3M1P9Q4	Allophycocyanin	*Cyanobacteria bacterium J069*	11	2	2
A0A2N5JMK4	Allophycocyanin	*Cyanobacteria bacterium M5B4*	17	3	3
A0A2T2RRT4	Allophycocyanin	*Cyanobacteria bacterium QH_9_48_43*	17	3	3
A0A2T2RXX7	Allophycocyanin	*Cyanobacteria bacterium QS_8_64_29*	17	3	3
A0A969LWT5	Phycocyanin subunit alpha	*Cyanobacteria bacterium RU_5_0*	23	2	2
A0A2T2RE55	Allophycocyanin	*Cyanobacteria bacterium SW_9_44_58*	17	3	3
A0A355DJA3	Allophycocyanin	*Cyanobacteria bacterium UBA11162*	12	2	2
A0A351KYP4	Allophycocyanin	*Cyanobacteria bacterium UBA11371*	17	3	3
A0A3B8K348	Phycocyanin subunit alpha	*Cyanobacteria bacterium UBA8553*	19	2	2
A0A352AEK4	Allophycocyanin	*Cyanobacteria bacterium UBA9273*	12	2	2
A0A965DJM1	Allophycocyanin	*Cyanobacteria bacterium WB6_1B_304*	11	2	2
Cyanomargarita calcarea					
A0A951QKN4	Allophycocyanin subunit alpha	*Cyanomargarita calcarea GSE-NOS-MK-12-04C*	11	2	2

Cylindrospermopsis curvispora					
A0A7H0F0K5	Allophycocyanin	*Cylindrospermopsis curvispora GIHE-G1*	11	2	2
A0A7H0EY98	Phycocyanin subunit alpha	*Cylindrospermopsis curvispora GIHE-G1*	23	2	2
A0A7H0EY99	Phycocyanin subunit beta	*Cylindrospermopsis curvispora GIHE-G1*	19	2	2
Cylindrospermopsis raciborskii					
A0A9Q5QUG8	Allophycocyanin	*Cylindrospermopsis raciborskii CENA302*	12	2	2
A0A1X4G9K1	Phycocyanin subunit beta	*Cylindrospermopsis raciborskii CENA303*	19	2	2
A0A853MD01	Phycocyanin subunit beta	*Cylindrospermopsis raciborskii CS-505*	19	2	2
A0A838WHR3	Allophycocyanin	*Cylindrospermopsis raciborskii CS-506_A*	12	2	2
A0A838WGC8	Phycocyanin subunit beta	*Cylindrospermopsis raciborskii CS-506_A*	31	3	4
Cylindrospermopsis sp.					
A0A0R0M5Z0	Phycocyanin	*Cylindrospermopsis sp. CR12*	23	2	2
A0A0R0MH79	Allophycocyanin	*Cylindrospermopsis sp. CR12*	11	2	2
Cylindrospermum sp.					
A0A926WTG6	Allophycocyanin	*Cylindrospermum sp. FACHB-282*	11	2	2
A0A1Z4QKC3	Phycobilisome protein	*Cylindrospermum sp. NIES-4074*	11	2	2
Desertifilum sp.					
A0A846E286	Allophycocyanin	*Desertifilum sp. SIO1I2*	12	2	2
Dolichospermum compactum					
A0A1Z4VAR2	Phycocyanin alpha subunit	*Dolichospermum compactum NIES-806*	34	3	3
A0A1Z4V5K5	Phycocyanin	*Dolichospermum compactum NIES-806*	17	3	3
A0A1Z4VAT7	Phycocyanin beta subunit	*Dolichospermum compactum NIES-806*	52	6	7
Dolichospermum flos-aquae					
A0A6H2C1Q0	Phycocyanin subunit alpha	*Dolichospermum flos-aquae CCAP 1403/13F*	19	2	2
Dolichospermum sp.					
A0AAW6JTW9	Phycocyanin subunit alpha	*Dolichospermum sp. ST_sed8*	19	2	2
A0AAW6JT81	Allophycocyanin subunit alpha	*Dolichospermum sp. ST_sed8*	11	2	2
A0A5C0DQG9	C-phycocyanin alpha chain	*Dolichospermum sp. UHCC 0315A*	19	2	2
A0A5C0DNG5	C-phycocyanin beta chain	*Dolichospermum sp. UHCC 0315A*	31	3	3
Drouetiella hepatica					
A0A951Q8B1	Allophycocyanin subunit alpha	*Drouetiella hepatica Uher 2000/2452*	11	2	2
Dulcicalothrix desertica					
A0A433VBJ0	Allophycocyanin alpha chain	*Dulcicalothrix desertica PCC 7102*	11	2	2
filamentous cyanobacterium					
A0A2T1DPM3	Allophycocyanin	*filamentous cyanobacterium CCP2*	12	2	2
A0A2T1D683	Phycocyanin subunit alpha	*filamentous cyanobacterium CCP2*	23	2	2
A0A2T2WBC5	Allophycocyanin	*filamentous cyanobacterium CCP3*	12	2	2
A0A2P8WFG0	Allophycocyanin	*filamentous cyanobacterium CCP5*	12	2	2
A0A2T1EZJ2	Allophycocyanin	*filamentous cyanobacterium Phorm 46*	12	2	2
Fischerella thermalis					
G6FU34	Phycocyanin	*Fischerella thermalis JSC-11*	11	2	2
Fortiea sp.					
A0A8J7D8J8	Phycocyanin subunit alpha	*Fortiea sp. LEGE XX443*	23	2	2
Geitlerinema sp.					
K9S897	Allophycocyanin alpha subunit apoprotein	*Geitlerinema sp. PCC 7407*	11	2	2
Geminocystis sp.					

A0A0D6AHF2	Allophycocyanin alpha chain	*Geminocystis sp. NIES-3708*	12	2	2
A0A0D6ANR5	Allophycocyanin alpha chain	*Geminocystis sp. NIES-3709*	12	2	2
Gloeobacter kilaueensis					
U5QFD5	Allophycocyanin, beta subunit	*Gloeobacter kilaueensis (strain ATCC BAA-2537 / CCAP 1431/1 / ULC 316 / JS1)*	11	2	2
Gloeobacter violaceus					
Q7NL80	Allophycocyanin alpha subunit	*Gloeobacter violaceus (strain ATCC 29082 / PCC 7421)*	11	2	2
Gloeobacterales cyanobacterium					
A0A925XSP1	Allophycocyanin	*Gloeobacterales cyanobacterium ES-bin-141*	17	3	3
A0A925ISE1	Allophycocyanin	*Gloeobacterales cyanobacterium ES-bin-313*	11	2	2
Gloeocapsa sp.					
A0A6P2AEI5	Allophycocyanin	*Gloeocapsa sp. DLM2.Bin57*	17	3	3
L8LMG2	Allophycocyanin alpha subunit apoprotein	*Gloeocapsa sp. PCC 73106*	11	2	2
Gloeocapsopsis sp.					
A0A2G7GIJ4	Allophycocyanin	*Gloeocapsopsis sp. IPPAS B-1203*	17	3	3
Gomphosphaeria aponina					
A0A941GXV2	Allophycocyanin subunit alpha	*Gomphosphaeria aponina SAG 52.96*	17	3	3
Leptolyngbya boryana					
A0AA96WUE2	Allophycocyanin subunit alpha	*Leptolyngbya boryana CZ1*	17	3	3
Leptolyngbya cf. ectocarpi					
A0A929FCN5	Allophycocyanin subunit alpha	*Leptolyngbya cf. ectocarpi LEGE 11479*	12	2	2
Leptolyngbya foveolarum					
A0A2W4WL51	Allophycocyanin	*Leptolyngbya foveolarum*	17	3	3
Leptolyngbya sp					
A0A2W7BS63	Allophycocyanin	*Leptolyngbya sp*	12	2	2
A0A2W7ATZ9	Allophycocyanin	*Leptolyngbya sp*	17	3	3
A0A651DVI7	Allophycocyanin	*Leptolyngbya sp. DLM2.Bin15*	11	2	2
A0A926U7C6	Allophycocyanin	*Leptolyngbya sp. FACHB-261*	11	2	2
A0A926YFD0	Allophycocyanin	*Leptolyngbya sp. FACHB-36*	11	2	2
A0A1Q8ZG15	Allophycocyanin	*Leptolyngbya sp. 'hensonii'*	12	2	2
U9VQP6	Allophycocyanin subunit alpha	*Leptolyngbya sp. Heron Island J*	11	2	2
A0A0N7Z574	Allophycocyanin alpha chain	*Leptolyngbya sp. NIES-2104*	17	3	3
A0A0S3TXT1	Allophycocyanin alpha subunit	*Leptolyngbya sp. NIES-3755*	12	2	2
A0AAT8XUK1	C-phycocyanin alpha chain	*Leptolyngbya sp. O-77*	23	2	2
A0A969AXA5	Allophycocyanin	*Leptolyngbya sp. RL_3_1*	11	2	2
A0A6I5RCW1	Allophycocyanin	*Leptolyngbya sp. SIO1E4*	17	3	3
Leptolyngbyaceae cyanobacterium					
A0A969RT93	Allophycocyanin	*Leptolyngbyaceae cyanobacterium CRU_2_3*	11	2	2
A0A969UNB4	Allophycocyanin	*Leptolyngbyaceae cyanobacterium CSU_1_3*	11	2	2
A0A925BUN4	Allophycocyanin	*Leptolyngbyaceae cyanobacterium LF-bin-113*	11	2	2
A0A969TMT1	Allophycocyanin	*Leptolyngbyaceae cyanobacterium RU_5_1*	11	2	2
A0A968U328	Allophycocyanin	*Leptolyngbyaceae cyanobacterium SM1_4_3*	12	2	2
Limnofasciculus baicalensis					
A0AAE3GU58	Allophycocyanin	*Limnofasciculus baicalensis BBK-W-15*	11	2	2
Limnoraphis robusta					
A0A0F5YL91	Allophycocyanin	*Limnoraphis robusta CS-951*	12	2	2

Lyngbya aestuarii					
U7QGG4	Allophycocyanin alpha chain	*Lyngbya aestuarii BL J*	12	2	2
Mastigocladus laminosus					
P00315	Allophycocyanin alpha chain	*Mastigocladus laminosus*	18	3	3
Merismopedia glauca					
A0A2T1BZC4	Allophycocyanin	*Merismopedia glauca CCAP 1448/3*	12	2	2
Merismopedia sp.					
A0A6P0Z1J5	Allophycocyanin	*Merismopedia sp. SIO2A8*	11	2	2
Microcoleaceae cyanobacterium					
A0A355CEP6	Allophycocyanin	*Microcoleaceae cyanobacterium UBA10368*	17	3	3
A0A349JQC7	Allophycocyanin	*Microcoleaceae cyanobacterium UBA11344*	17	3	3
Microcoleus sp.					
A0A970AMN8	Allophycocyanin	*Microcoleus sp. CSU_2_2*	17	3	3
A0A926TZ30	Allophycocyanin	*Microcoleus sp. FACHB-1515*	11	2	2
A0A926YAQ0	Allophycocyanin	*Microcoleus sp. FACHB-68*	17	3	3
Microseira wollei					
A0AAV3X7D5	Allophycocyanin alpha subunit	*Microseira wollei NIES-4236*	12	2	2
Mojavia pulchra					
A0A951UFQ2	Phycocyanin subunit alpha	*Mojavia pulchra JT2-VF2*	23	2	2
A0A951UF51	Allophycocyanin subunit alpha	*Mojavia pulchra JT2-VF2*	12	2	2
A0A951UFB7	Phycocyanin subunit beta	*Mojavia pulchra JT2-VF2*	21	2	3
Neosynechococcus sphagnicola					
A0A098TLR2	Allophycocyanin	*Neosynechococcus sphagnicola sy1*	12	2	2
Nodularia harveyana					
A0A8J9TPA9	Allophycocyanin alpha chain	*Nodularia harveyana CCAP 1452/1*	11	2	2
Nodularia sp.					
A0A218Q2C8	Phycocyanin	*Nodularia sp. NIES-3585*	12	2	2
Nodularia spumigena					
A0A166K5J4	Allophycocyanin	*Nodularia spumigena CENA596*	17	3	3
Nostoc azollae					
D7E337	Phycocyanin, alpha subunit	*Nostoc azollae (strain 0708)*	19	2	2
Nostoc cycadae					
A0A2H6LMW1	Allophycocyanin subunit alpha apoprotein	*Nostoc cycadae WK-1*	12	2	2
Nostoc edaphicum					
A0A7D7QL00	Allophycocyanin	*Nostoc edaphicum CCNP1411*	11	2	2
Nostoc minutum					
A0A367RWU6	Allophycocyanin	*Nostoc minutum NIES-26*	12	2	2
Nostoc piscinale					
A0A0M4TKC4	Phycocyanin	*Nostoc piscinale CENA21*	23	2	2
Nostoc sp.					
A0A252DGX6	Allophycocyanin	*Nostoc sp. 106C*	12	2	2
A0A367PVT9	Allophycocyanin	*Nostoc sp. ATCC 43529*	17	3	3
A0A2I8A176	Allophycocyanin	*Nostoc sp. CENA543*	12	2	2
A0A926VVH8	Allophycocyanin	*Nostoc sp. FACHB-888*	11	2	2
A0A1E2WL85	Allophycocyanin	*Nostoc sp. KVJ20*	11	2	2
A0A939IF90	Allophycocyanin	*Nostoc sp. LPT*	11	2	2

A0A1C0VMD8	Allophycocyanin	*Nostoc sp. MBR 210*	17	3	3
A0A1Z4IDX0	Phycobilisome protein	*Nostoc sp. NIES-2111*	17	3	3
A0A235IB78	Allophycocyanin	*Nostoc sp. 'Peltigera membranacea cyanobiont' 210A*	11	2	2
A0A235J6M6	Allophycocyanin	*Nostoc sp. 'Peltigera membranacea cyanobiont' 232*	11	2	2
A0A2L2NAQ3	Allophycocyanin subunit alpha	*Nostoc sp. 'Peltigera membranacea cyanobiont' N6*	11	2	2
A0A252E112	Allophycocyanin	*Nostoc sp. T09*	12	2	2
A0A4Y5PW22	Allophycocyanin alpha	*Nostoc sp. WR13*	17	3	3
Nostoc sphaeroides					
A0A5P8W6Q0	ApcA, allophycocyanin alpha subunit	*Nostoc sphaeroides CCNUC1*	17	3	3
Nostocales cyanobacterium					
A0A9E6RYN0	Allophycocyanin	*Nostocales cyanobacterium*	11	2	2
A0A9E6UT21	Phycocyanin subunit alpha	*Nostocales cyanobacterium*	23	2	2
A0A1Y0RS29	Allophycocyanin	*Nostocales cyanobacterium HT-58-2*	12	2	2
A0A1Y0REZ5	Phycocyanin subunit alpha	*Nostocales cyanobacterium HT-58-2*	23	2	2
Oculatella sp.					
A0A8J6VP43	Phycocyanin subunit beta	*Oculatella sp. FACHB-28*	19	2	2
Oscillatoria nigro-viridis					
A0A8J9XFI1	Allophycocyanin alpha chain	*Oscillatoria nigro-viridis*	17	3	3
Oscillatoriales cyanobacterium					
A0A1J5GL20	Allophycocyanin	*Oscillatoriales cyanobacterium CG2 30 44 21*	23	4	4
A0A968Y292	Allophycocyanin	*Oscillatoriales cyanobacterium RU 3 3*	11	2	2
A0A968IZQ6	Allophycocyanin	*Oscillatoriales cyanobacterium SM2 1 8*	17	3	3
A0A7C3PFJ8	Allophycocyanin	*Oscillatoriales cyanobacterium SpSt-418*	11	2	2
A0A1C0VYM6	Allophycocyanin	*Oscillatoriales cyanobacterium USR001*	12	2	2
Pantanalinema sp.					
A0A937EXK1	Allophycocyanin	*Pantanalinema sp. GBBB05*	11	2	2
Parathermosynechococcus lividus					
A0A2D2Q321	Allophycocyanin	*Parathermosynechococcus lividus PCC 6715*	12	2	2
Pelatocladus maniniholoensis					
A0A9E3HAM7	Allophycocyanin subunit alpha	*Pelatocladus maniniholoensis HA4357-MV3*	11	2	2
Phormidium ambiguum					
A0A1U7IE87	Allophycocyanin	*Phormidium ambiguum IAM M-71*	17	3	3
Phormidium sp.					
A0A522XEV4	Allophycocyanin	*Phormidium sp. SL48-SHIP*	12	2	2
Phormidium tenue					
A0A1U7JAT3	Allophycocyanin	*Phormidium tenue NIES-30*	12	2	2
Planktothricoides sp.					
A0A0M1JPI9	Allophycocyanin	*Planktothricoides sp. SR001*	12	2	2
Planktothrix agardhii					
A0A4P5ZCB3	Allophycocyanin alpha subunit	*Planktothrix agardhii CCAP 1459/11A*	12	2	2
Planktothrix pseudagardhii					
A0A9W4G280	Allophycocyanin alpha chain	*Planktothrix pseudagardhii*	12	2	2
Planktothrix sp.					
A0A354WH48	Allophycocyanin	*Planktothrix sp. UBA10369*	12	2	2
Plectolyngbya sp.					

A0A951NWS1	Allophycocyanin subunit alpha	*Plectolyngbya sp. WJT66-NPBG17*	17	3	3
Plectonema cf. radiosum					
A0A8J7K299	Phycocyanin subunit alpha	*Plectonema cf. radiosum LEGE 06105*	19	2	2
Pleurocapsa sp.					
A0A968IRB4	Allophycocyanin	*Pleurocapsa sp. SU_5_0*	23	4	4
Pleurocapsales cyanobacterium					
A0A929FWD4	Allophycocyanin	*Pleurocapsales cyanobacterium LEGE 06147*	11	2	2
A0A928ZCZ4	Allophycocyanin	*Pleurocapsales cyanobacterium LEGE 10410*	11	2	2
Pseudanabaena cinerea					
A0A926UTP6	Allophycocyanin	*Pseudanabaena cinerea FACHB-1277*	17	3	3
Pseudanabaena frigida					
A0A2W4Y8U6	Allophycocyanin	*Pseudanabaena frigida*	23	4	4
A0A2W4W8I7	Allophycocyanin subunit beta	*Pseudanabaena frigida*	19	2	2
Pseudanabaena sp					
A0A2W7AAG3	Allophycocyanin subunit beta	*Pseudanabaena sp*	19	2	2
A0A2W6ZMB9	Allophycocyanin subunit beta	*Pseudanabaena sp*	19	2	2
A0A352JCX6	Allophycocyanin	*Pseudanabaena sp*	17	3	3
A0A2W6Z5S6	Allophycocyanin	*Pseudanabaena sp*	23	4	4
A0A2W7C4P5	Allophycocyanin	*Pseudanabaena sp*	17	3	3
A0A2Z5X2N9	Allophycocyanin alpha subunit	*Pseudanabaena sp. ABRG5-3*	23	4	4
V5NXK8	ApcB	*Pseudanabaena sp. lw0831*	19	2	2
V5NZF0	Phycocyanin beta chain	*Pseudanabaena sp. lw0831*	15	2	2
A0A256BGA7	Phycocyanin subunit beta	*Pseudanabaena sp. SR411*	15	2	2
A0A6C2DZJ7	Allophycocyanin	*Pseudanabaena sp. UWO310*	17	3	3
A0A652Z043	Allophycocyanin	*Pseudanabaena sp. UWO311*	23	4	4
A0A6N2JZB3	Phycocyanin subunit beta	*Pseudanabaena sp. UWO311*	16	2	2
Pseudanabaena tenuis					
Q52446	C-phycocyanin-1 beta subunit	*Pseudanabaena tenuis (strain PCC 7409)*	15	2	2
Pseudanabaenaceae cyanobacterium					
A0A928VES5	Allophycocyanin subunit alpha	*Pseudanabaenaceae cyanobacterium LEGE 13415*	17	3	3
Pseudocalidococcus azoricus					
A0AAE4FQ17	Allophycocyanin subunit alpha	*Pseudocalidococcus azoricus BACA0444*	12	2	2
Richelia sinica					
A0A975T907	Allophycocyanin alpha chain	*Richelia sinica FACHB-800*	12	2	2
A0A975T7E6	Phycocyanin beta subunit	*Richelia sinica FACHB-800*	21	2	3
Richelia sp.					
A0A969QCR1	Allophycocyanin	*Richelia sp. CSU 2 1*	17	3	3
A0A969MJ06	Allophycocyanin	*Richelia sp. RM2 1 2*	11	2	2
A0A968WAI6	Phycocyanin subunit alpha	*Richelia sp. SM1 7 0*	19	2	2
Rivularia sp.					
A0A949TLI6	Phycocyanin subunit alpha	*Rivularia sp.*	19	2	2
A0A949T6V0	Allophycocyanin subunit alpha	*Rivularia sp. MS3*	17	3	3
K9REV4	Phycocyanin, alpha subunit	*Rivularia sp. PCC 7116*	19	2	2
Scytonema hofmannii					
A0A139WXF7	Allophycocyanin	*Scytonema hofmannii PCC 7110*	12	2	2
Scytonema sp.					

A0AA91GVQ4	Allophycocyanin	*Scytonema sp. HK-05*	17	3	3
Sphaerospermopsis reniformis					
A0A479ZWB6	Phycocyanin subunit alpha	*Sphaerospermopsis reniformis*	34	3	3
A0A480A8R2	Phycocyanin	*Sphaerospermopsis reniformis*	11	2	2
Sphaerospermopsis sp.					
A0A846EIF2	Allophycocyanin	*Sphaerospermopsis sp. SIO1G1*	11	2	2
A0A846EIS8	Phycocyanin subunit alpha	*Sphaerospermopsis sp. SIO1G1*	23	2	2
Stanieria sp.					
A0A140KB32	Phycobilisome protein	*Stanieria sp. NIES-3757*	12	2	2
Stenomitos frigidus					
A0A2T1DX81	Allophycocyanin	*Stenomitos frigidus ULC18*	17	3	3
Synechococcales cyanobacterium					
A0A930TJP2	Phycocyanin subunit alpha	*Synechococcales cyanobacterium C42_A2020_086*	23	2	2
A0A978U6M2	Allophycocyanin subunit alpha	*Synechococcales cyanobacterium M55_K2018_004*	11	2	2
Synechococcus sp.					
A0A2G8PFN9	Allophycocyanin subunit alpha	*Synechococcus sp. 60AY4M2*	12	2	2
A0A7T1HWE2	Phycocyanin subunit beta	*Synechococcus sp. CBW1004*	12	2	2
A0A1Q2U063	Allophycocyanin alpha subunit	*Synechococcus sp. NIES-970*	12	2	2
A0A1L6BXH6	Allophycocyanin subunit alpha (Fragment)	*Synechococcus sp. OH20*	12	2	2
A0A1L6BXH8	Allophycocyanin subunit alpha (Fragment)	*Synechococcus sp. OH28*	12	2	2
K9STK0	Allophycocyanin alpha subunit apoprotein	*Synechococcus sp. PCC 7502*	11	2	2
Synechocystis sp.					
Q01951	Allophycocyanin alpha chain	*Synechocystis sp. (strain ATCC 27184 / PCC 6803 / Kazusa)*	12	2	2
Q02923	Allophycocyanin alpha chain	*Synechocystis sp. (strain PCC 6714)*	12	2	2
Thermoleptolyngbya oregonensis					
A0AA96Y2Z6	Phycocyanin subunit alpha	*Thermoleptolyngbya oregonensis NK1-22*	23	2	2
Thermosynechococcus sp.					
A0A5C2M6M9	Allophycocyanin	*Thermosynechococcus sp. CL-1*	17	3	3
Thermosynechococcus vestitus					
P50030	Allophycocyanin alpha chain	*Thermosynechococcus vestitus (strain NIES-2133 / IAM M-273 / BP-1)*	17	3	3
Tolypothrix bouteillei					
A0A0C1QT48	Allophycocyanin	*Tolypothrix bouteillei VB521301*	17	3	3
Tolypothrix sp.					
A0A218QT84	Phycocyanin beta subunit	*Tolypothrix sp. NIES-4075*	20	2	2
Trichormus variabilis					
Q3M9V1	Phycobilisome protein	*Trichormus variabilis (strain ATCC 29413 / PCC 7937)*	12	2	2
A0A3S1CEI7	Allophycocyanin alpha chain	*Trichormus variabilis SAG 1403-4b*	17	3	3
Tychonema bourrellyi					
A0A2G4EVV9	Allophycocyanin	*Tychonema bourrellyi FEM_GT703*	17	3	3
Umezakia ovalisporum					
A0AA43GX24	Allophycocyanin subunit alpha	*Umezakia ovalisporum FSS-62*	11	2	2

Table 3: Proteins from cyanobacterial strains detected by trypsin digestion followed by LC-MS/MS analysis from lake water 4.

Accession	Description	Species	Coverage [%]	# Peptides	# PSMs
Aphanothece hegewaldii					
A0A2T1LW89	Allophycocyanin	*Aphanothece hegewaldii CCALA 016*	14	2	2
Chroococcus sp.					
A0A8J2TI46	Phycobilisome protein	*Chroococcus sp. FPU101*	14	2	2
Geminocystis sp.					
A0A978T651	Allophycocyanin	*Geminocystis sp. M7585_C2015_104*	14	2	2
Gloeothece citriformis					
B7KKS9	Phycobilisome protein	*Gloeothece citriformis (strain PCC 7424)*	14	2	2
Gloeothece verrucosa					
E0UA88	Phycocyanin	*Gloeothece verrucosa (strain PCC 7822)*	14	2	2
Microcystis aeruginosa					
A0A510PK91	Phycocyanin subunit beta	*Microcystis aeruginosa 11-30S32*	25	2	2
A0A857D0J1	Phycocyanin subunit beta	*Microcystis aeruginosa FD4*	25	2	2
A0A552EV89	Phycocyanin subunit beta	*Microcystis aeruginosa Ma_MB_F_20061100_S20D*	25	2	2
A0A552E7C2	Phycocyanin subunit beta	*Microcystis aeruginosa Ma_MB_S_20031200_S102*	25	2	2
A0A552FHF8	Allophycocyanin	*Microcystis aeruginosa Ma_QC_Ca_00000000_S207*	14	2	2
A0A552G5E8	Allophycocyanin	*Microcystis aeruginosa Ma_QC_Ch_20071001_S25D*	14	2	2
A0A6H9GLU2	Phycocyanin beta subunit	*Microcystis aeruginosa NIES-3804*	25	2	2
I4HAS2	C-phycocyanin beta chain	*Microcystis aeruginosa PCC 9807*	25	2	2
Microcystis flos-aquae					
A0A3E0KYJ4	Phycocyanin subunit beta	*Microcystis flos-aquae TF09*	25	2	2
Microcystis sp.					
A0A2L2XUH8	Phycocyanin beta chain	*Microcystis sp. 0824*	25	2	2
A0A552ARD9	Allophycocyanin	*Microcystis sp. M_OC_Ca_00000000_C217Col*	14	2	2
A0A2P1UGR1	Phycocyanin subunit beta	*Microcystis sp. MC19*	25	2	2
Microcystis viridis					
A0A3G9JUA6	Allophycocyanin alpha subunit	*Microcystis viridis NIES-102*	14	2	2
Pleurocapsa sp.					
K9TAS6	Allophycocyanin alpha subunit apoprotein	*Pleurocapsa sp. PCC 7327*	14	2	2

Table 4: Proteins from cyanobacterial strains detected by trypsin digestion followed by LC-MS/MS analysis from lake water 5.

Accession	Description	Species	Coverage [%]	# Peptides	# PSMs
Adonisia turfae					
A0A6M0RU12	Allophycocyanin subunit beta	*Adonisia turfae CCMR0081*	19	2	5
Aetokthonos hydrillicola					
A0AAP5I9Q5	Allophycocyanin subunit alpha	*Aetokthonos hydrillicola Thurmond2011*	25	3	4
A0AAP5I8M6	Phycocyanin subunit alpha	*Aetokthonos hydrillicola Thurmond2011*	10	2	4
A0AAP5I693	Phycocyanin subunit beta	*Aetokthonos hydrillicola Thurmond2011*	12	2	2
aff. Roholtiella sp.					
A0A8J7D6K0	Allophycocyanin subunit alpha	*aff. Roholtiella sp. LEGE 12411*	11	2	2
A0A8J7A524	Allophycocyanin	*aff. Roholtiella sp. LEGE 12411*	32	3	5
Aliterella atlantica					
A0A0D8ZXX5	Allophycocyanin	*Aliterella atlantica CENA595*	19	2	6
A0A0D8ZST4	Phycocyanin	*Aliterella atlantica CENA595*	10	2	9
A0A0D8ZUK5	Allophycocyanin	*Aliterella atlantica CENA595*	25	3	4
Alkalinema sp.					
A0A251WM95	Phycocyanin subunit alpha	*Alkalinema sp. CACIAM 70d*	10	2	4
A0A251WJT7	Allophycocyanin subunit beta	*Alkalinema sp. CACIAM 70d*	14	2	2
A0A251WDP1	Phycocyanin subunit alpha	*Alkalinema sp. CACIAM 70d*	10	2	4
A0A968X4F9	Allophycocyanin subunit beta	*Alkalinema sp. RU_4_3*	17	2	3
Amazonocrinis nigriterrae					
A0A8J7HZF8	Allophycocyanin	*Amazonocrinis nigriterrae CENA67*	19	2	4
Anabaena cylindrica					
P07325	Allophycocyanin alpha chain	*Anabaena cylindrica*	42	5	10
K9ZNR0	Phycocyanin	*Anabaena cylindrica (strain ATCC 27899 / PCC 7122)*	24	3	5
Anabaena sp.					
K7WMD9	Phycobilisome protein ApcA	*Anabaena sp. 90*	42	5	10
K7WTL4	Phycocyanin beta subunit	*Anabaena sp. 90*	14	2	3
A0A1B7WMN4	Phycocyanin	*Anabaena sp. CRKS33*	44	6	14
A0A1B7WSR8	Allophycocyanin	*Anabaena sp. CRKS33*	64	7	12
A0A1B7WMQ4	Phycocyanin	*Anabaena sp. CRKS33*	59	8	19
A0A926ULC4	Allophycocyanin	*Anabaena sp. FACHB-1237*	36	4	9
A0A926UKC4	Allophycocyanin subunit beta	*Anabaena sp. FACHB-1237*	24	3	7
A0A926ULV0	Allophycocyanin	*Anabaena sp. FACHB-1237*	22	4	6
A0A1B7V0A1	Allophycocyanin	*Anabaena sp. LE011-02*	42	5	10
A0A9X1GP51	Allophycocyanin	*Anabaena sp. PCC 7938*	42	5	10
A0A9X1GK69	Allophycocyanin subunit beta	*Anabaena sp. PCC 7938*	24	3	7
A0A3D4C397	Phycocyanin subunit alpha	*Anabaena sp. UBA12330*	26	3	11
A0A3D4C1K4	Allophycocyanin subunit beta	*Anabaena sp. UBA12330*	71	8	18
A0A1B7VSW7	Allophycocyanin	*Anabaena sp. WA113*	48	5	6
A0A455KZV8	Phycocyanin B	*uncultured Anabaena sp*	14	2	3
Anabaena sphaerica					
A0A927A4K0	Allophycocyanin	*Anabaena sphaerica FACHB-251*	53	5	10
A0A927A3E3	Allophycocyanin subunit beta	*Anabaena sphaerica FACHB-251*	24	3	7

A0A927A4I2	Phycocyanin subunit alpha	*Anabaena sphaerica FACHB-251*	11	2	6
A0A926WM41	Allophycocyanin	*Anabaena sphaerica FACHB-251*	41	6	10
A0A927A3U9	Phycocyanin subunit beta	*Anabaena sphaerica FACHB-251*	21	2	4
Anabaenopsis circularis					
A0A1Z4GCJ2	Phycobilisome protein	*Anabaenopsis circularis NIES-21*	12	2	2
Anabaenopsis elenkinii					
A0A7S6TZF6	Allophycocyanin subunit alpha	*Anabaenopsis elenkinii CCIBt3563*	19	2	3
Aphanizomenon flos-aquae					
A0A1B7VQT7	Allophycocyanin	*Aphanizomenon flos-aquae LD13*	18	2	4
A0A1B7WBM0	Phycocyanin	*Aphanizomenon flos-aquae WA102*	18	3	10
A0A1B7X4P2	Allophycocyanin	*Aphanizomenon flos-aquae WA102*	58	6	11
A0A1B7X5R9	Allophycocyanin	*Aphanizomenon flos-aquae WA102*	18	2	4
A0A1B7WZK6	Phycocyanin	*Aphanizomenon flos-aquae WA102*	21	2	4
Aphanizomenon sp.					
A0A844ICS2	Phycocyanin subunit beta	*Aphanizomenon sp. UHCC 0183*	14	2	3
Aphanothece hegewaldii					
A0A2T1LW90	Allophycocyanin subunit beta	*Aphanothece hegewaldii CCALA 016*	30	3	5
A0A2T1LW89	Allophycocyanin	*Aphanothece hegewaldii CCALA 016*	15	2	2
Aphanothece sacrum					
A0A401ICI1	Allophycocyanin b chain	*Aphanothece sacrum FPU1*	27	3	5
A0A401ILC5	Phycocyanin b subunit	*Aphanothece sacrum FPU1*	12	2	2
Atlanticothrix silvestris					
A0A8J7H5A3	Allophycocyanin subunit beta	*Atlanticothrix silvestris CENA357*	14	2	3
A0A8J7H708	Allophycocyanin subunit alpha	*Atlanticothrix silvestris CENA357*	19	2	3
Brunnivagina elsteri					
A0A2A2TIY1	Allophycocyanin subunit beta	*Brunnivagina elsteri CCALA 953*	14	2	3
A0A2A2TBP9	Phycocyanin subunit alpha	*Brunnivagina elsteri CCALA 953*	10	2	4
Calothrix sp					
A0A0T7BME1	Allophycocyanin	*Calothrix sp. 336/3*	24	3	6
A0A0T7BMD2	Allophycocyanin	*Calothrix sp. 336/3*	25	3	4
A0A930XG57	Allophycocyanin subunit alpha	*Calothrix sp. C42 A2020 038*	19	2	3
A0A9E5V8C2	Allophycocyanin	*Calothrix sp. CSU 2 0*	25	3	4
A0A9E5V5E7	Allophycocyanin subunit beta	*Calothrix sp. CSU 2 0*	14	2	3
A0A1Z4NJH9	Phycocyanin	*Calothrix sp. NIES-3974*	36	4	9
A0A1Z4NJK4	Allophycocyanin beta subunit	*Calothrix sp. NIES-3974*	14	2	3
A0A1Z4NQ20	C-phycocyanin-1 alpha chain	*Calothrix sp. NIES-3974*	10	2	4
A0A1Z4R810	Phycobilisome protein	*Calothrix sp. NIES-4101*	25	3	4
A0A1Z4R805	Allophycocyanin, beta subunit ApcB	*Calothrix sp. NIES-4101*	16	2	4
A0A1Z4RG19	Phycocyanin alpha subunit	*Calothrix sp. NIES-4101*	10	2	4
K9UXN4	Phycocyanin	*Calothrix sp. PCC 6303*	19	2	3
K9UZP4	Allophycocyanin, beta subunit	*Calothrix sp. PCC 6303*	14	2	3
K9V8G6	Phycocyanin, beta subunit	*Calothrix sp. PCC 6303*	12	2	2
K9PPW9	Allophycocyanin alpha-B subunit apoprotein	*Calothrix sp. PCC 7507*	32	3	5
Chamaesiphon sp.					
A0A969QKP1	Allophycocyanin subunit beta	*Chamaesiphon sp. CSU_1_12*	19	2	5
Chloroflexaceae bacterium					

A0A968V434	Allophycocyanin subunit beta	*Chloroflexaceae bacterium*	30	3	6
Chlorogloea sp.					
A0A2T1EA96	Phycocyanin subunit alpha	*Chlorogloea sp. CCALA 695*	10	2	9
A0A2T1EFR3	Allophycocyanin	*Chlorogloea sp. CCALA 695*	25	3	4
Chlorogloeopsis fritschii					
A0A3S0Y3A8	Allophycocyanin alpha chain	*Chlorogloeopsis fritschii PCC 6912*	34	3	4
A0A433NFC3	Allophycocyanin-B	*Chlorogloeopsis fritschii PCC 6912*	19	2	4
Chondrocystis sp.					
A0A1Z4RRS0	Phycobilisome protein	*Chondrocystis sp. NIES-4102*	12	2	2
Chroococcidiopsis sp.					
A0A2P8QIZ5	Allophycocyanin	*Chroococcidiopsis sp. CCALA 051*	40	4	6
A0A2S6VGH5	Allophycocyanin subunit beta	*Chroococcidiopsis sp. TS-821*	19	2	4
A0A2S6VGG8	Allophycocyanin	*Chroococcidiopsis sp. TS-821*	25	3	4
Chroococcus sp.					
A0A8J2TC53	Allophycocyanin, beta subunit	*Chroococcus sp. FPU101*	30	3	5
Chrysosporum bergii					
A0AA43GP68	Allophycocyanin subunit alpha	*Chrysosporum bergii ANA360D*	19	2	3
Coleofasciculaceae cyanobacterium					
A0A969FL11	Allophycocyanin subunit beta	*Coleofasciculaceae cyanobacterium RL_1_1*	28	3	5
A0A968SHU1	Allophycocyanin subunit beta	*Coleofasciculaceae cyanobacterium SM2_1_6*	19	2	3
A0A968SF93	Phycocyanin subunit beta	*Coleofasciculaceae cyanobacterium SM2_1_6*	26	4	9
Coleofasciculus chthonoplastes					
B4VSN2	Phycobilisome protein	*Coleofasciculus chthonoplastes PCC 7420*	21	2	3
B4VSN1	Allophycocyanin, beta subunit	*Coleofasciculus chthonoplastes PCC 7420*	24	3	6
B4VWT1	Phycocyanin, alpha subunit	*Coleofasciculus chthonoplastes PCC 7420*	10	2	4
Coleofasciculus sp.					
A0A926XI84	Allophycocyanin	*Coleofasciculus sp. FACHB-SPT36*	20	2	3
A0A929FQ32	Allophycocyanin subunit beta	*Coleofasciculus sp. LEGE 07092*	24	3	7
A0A929AQ34	Allophycocyanin subunit alpha	*Coleofasciculus sp. LEGE 07092*	12	2	2
A0A929AKM3	Phycocyanin subunit alpha	*Coleofasciculus sp. LEGE 07092*	10	2	4
A0A6J4IPJ0	Allophycocyanin alpha chain	*uncultured Coleofasciculus sp*	20	2	3
Cuspidothrix issatschenkoi					
A0A2S6CX50	Allophycocyanin	*Cuspidothrix issatschenkoi CHARLIE-1*	31	4	5
A0A2S6CQK6	Phycocyanin subunit alpha	*Cuspidothrix issatschenkoi CHARLIE-1*	23	2	3
A0A2S6CYN1	Allophycocyanin	*Cuspidothrix issatschenkoi CHARLIE-1*	32	5	8
A0A2S6CQK9	Phycocyanin subunit beta	*Cuspidothrix issatschenkoi CHARLIE-1*	21	2	4
Cyanobacteria bacterium					
A0A966F8Q4	Allophycocyanin subunit beta	*Cyanobacteria bacterium CG_2015-22_32_23*	30	3	5
A0A926Y5C9	Allophycocyanin subunit beta	*Cyanobacteria bacterium FACHB-502*	19	2	3
A0A3M1P8I6	Allophycocyanin subunit beta	*Cyanobacteria bacterium J069*	19	2	5
A0A3M1P9Q4	Allophycocyanin	*Cyanobacteria bacterium J069*	20	2	2
A0A2N5JMN3	Allophycocyanin subunit beta	*Cyanobacteria bacterium M5B4*	22	2	4
A0A2N5JMK4	Allophycocyanin	*Cyanobacteria bacterium M5B4*	12	2	2
A0A2T2RRT4	Allophycocyanin	*Cyanobacteria bacterium QH_9_48_43*	26	3	4
A0A2T2RY45	Allophycocyanin subunit beta	*Cyanobacteria bacterium QS_8_64_29*	22	2	4
A0A2T2RXX7	Allophycocyanin	*Cyanobacteria bacterium QS_8_64_29*	26	3	4

A0A969IYW1	Allophycocyanin subunit beta	*Cyanobacteria bacterium RU_5_0*	19	2	4
A0A969LW31	Phycocyanin subunit beta	*Cyanobacteria bacterium RU_5_0*	22	3	4
A0A9D9KAG3	Allophycocyanin subunit beta	*Cyanobacteria bacterium SID2*	19	2	3
A0A2T2RE55	Allophycocyanin	*Cyanobacteria bacterium SW_9_44_58*	26	3	4
A0A355DKD1	Allophycocyanin subunit beta	*Cyanobacteria bacterium UBA11162*	52	6	11
A0A355DJA3	Allophycocyanin	*Cyanobacteria bacterium UBA11162*	20	2	3
A0A351KYP4	Allophycocyanin	*Cyanobacteria bacterium UBA11371*	12	2	2
A0A3B8K348	Phycocyanin subunit alpha	*Cyanobacteria bacterium UBA8553*	10	2	9
A0A352AEK5	Allophycocyanin subunit beta	*Cyanobacteria bacterium UBA9273*	24	3	6
A0A352AEK4	Allophycocyanin	*Cyanobacteria bacterium UBA9273*	25	3	4
A0A965DK79	Allophycocyanin subunit beta	*Cyanobacteria bacterium WB6_1B_304*	22	2	4
Cyanobacterium aponinum					
A0AAF0ZF69	Allophycocyanin subunit beta	*Cyanobacterium aponinum AL20115*	30	3	5
Cyanobacterium sp.					
A0A2K8WSI2	Allophycocyanin beta subunit ApcB	*Cyanobacterium sp. HL-69*	19	2	3
A0A1E5QZJ0	Allophycocyanin	*Cyanobacterium sp. IPPAS B-1200*	15	2	2
A0A930SX45	Allophycocyanin subunit beta	*Cyanobacterium sp. T60_A2020_053*	19	2	3
Cyanobacterium stanieri					
K9YI32	Allophycocyanin, beta subunit	*Cyanobacterium stanieri (strain ATCC 29140 / PCC 7202)*	30	3	5
Cyanobium sp					
A0A2W6ZZR9	Phycocyanin subunit alpha	*Cyanobium sp*	11	2	2
A0A081GN36	Phycocyanin	*Cyanobium sp. CACIAM 14*	11	2	2
B5IPK3	Phycocyanin, alpha subunit	*Cyanobium sp. PCC 7001*	10	2	4
Cyanobium usitatum					
A0A2P7MXC7	Phycocyanin subunit beta	*Cyanobium usitatum str. Tous*	7	2	2
Cyanomargarita calcarea					
A0A951QKN4	Allophycocyanin subunit alpha	*Cyanomargarita calcarea GSE-NOS-MK-12-04C*	19	2	3
Cyanothece sp.					
A0A3B8XYR2	Allophycocyanin subunit beta	*Cyanothece sp. UBA12306*	22	2	6
Cylindrospermopsis curvispora					
A0A7H0F0K5	Allophycocyanin	*Cylindrospermopsis curvispora GIHE-G1*	30	3	8
A0A7H0EY78	Allophycocyanin	*Cylindrospermopsis curvispora GIHE-G1*	25	3	5
Cylindrospermopsis raciborskii					
A0A9Q5QUG8	Allophycocyanin	*Cylindrospermopsis raciborskii CENA302*	36	4	9
A0A1X4G555	Allophycocyanin	*Cylindrospermopsis raciborskii CENA303*	15	2	3
A0A838WHR3	Allophycocyanin	*Cylindrospermopsis raciborskii CS-506_A*	25	3	4
A0A838WVU6	Allophycocyanin subunit beta	*Cylindrospermopsis raciborskii CS-506_A*	24	3	6
A0A838WGC8	Phycocyanin subunit beta	*Cylindrospermopsis raciborskii CS-506_A*	14	2	3
Cylindrospermopsis sp.					
A0A0R0MH79	Allophycocyanin	*Cylindrospermopsis sp. CR12*	30	3	8
Cylindrospermum sp.					
A0A926WUA2	Allophycocyanin subunit beta	*Cylindrospermum sp. FACHB-282*	14	2	5
A0A926WTG6	Allophycocyanin	*Cylindrospermum sp. FACHB-282*	25	3	4
A0A927A7C9	Allophycocyanin	*Cylindrospermum sp. FACHB-282*	32	3	5
A0A1Z4QKC3	Phycobilisome protein	*Cylindrospermum sp. NIES-4074*	12	2	2
A0A1Z4QIP7	Phycobilisome protein	*Cylindrospermum sp. NIES-4074*	32	3	5

Cylindrospermum stagnale					
K9WSW2	Allophycocyanin alpha subunit apoprotein	*Cylindrospermum stagnale PCC 7417*	12	2	2
K9WQF7	Allophycocyanin alpha-B subunit apoprotein	*Cylindrospermum stagnale PCC 7417*	32	3	5
Dactylococcopsis salina					
K9YXC1	Allophycocyanin alpha subunit apoprotein	*Dactylococcopsis salina (strain PCC 8305)*	20	2	2
Desertifilum sp.					
A0A846E286	Allophycocyanin	*Desertifilum sp. SIO1I2*	12	2	2
Dolichospermum compactum					
A0A1Z4VAR2	Phycocyanin alpha subunit	*Dolichospermum compactum NIES-806*	33	4	12
A0A1Z4V5K5	Phycocyanin	*Dolichospermum compactum NIES-806*	47	6	11
A0A1Z4V5I7	Allophycocyanin beta subunit	*Dolichospermum compactum NIES-806*	24	3	7
A0A1Z4UYP4	Phycobilisome protein	*Dolichospermum compactum NIES-806*	41	6	10
A0A1Z4VAT7	Phycocyanin beta subunit	*Dolichospermum compactum NIES-806*	14	2	3
Dolichospermum flos-aquae					
A0A6H2C1Q0	Phycocyanin subunit alpha	*Dolichospermum flos-aquae CCAP 1403/13F*	18	3	10
Dolichospermum planctonicum					
A0A480ANF7	Phycobilisome protein	*Dolichospermum planctonicum*	54	7	11
Dolichospermum sp.					
A0AAW6JTW9	Phycocyanin subunit alpha	*Dolichospermum sp. ST sed8*	18	3	10
A0AAW6JT81	Allophycocyanin subunit alpha	*Dolichospermum sp. ST sed8*	47	6	11
A0AAW6JT84	Allophycocyanin subunit beta	*Dolichospermum sp. ST sed8*	19	2	6
A0AAW6JNU7	Allophycocyanin	*Dolichospermum sp. ST sed8*	31	5	8
A0A844IMK7	Allophycocyanin subunit beta	*Dolichospermum sp. UHCC 0260*	24	3	7
A0A5C0DQG9	C-phycocyanin alpha chain	*Dolichospermum sp. UHCC 0315A*	18	3	10
A0A944CV26	Allophycocyanin	*Dolichospermum sp. WA123*	32	5	8
Drouetiella hepatica					
A0A951Q782	Allophycocyanin subunit beta	*Drouetiella hepatica Uher 2000/2452*	19	2	4
Dulcicalothrix desertica					
A0A3S1CAQ3	Allophycocyanin beta chain	*Dulcicalothrix desertica PCC 7102*	24	3	7
A0A433VBJ0	Allophycocyanin alpha chain	*Dulcicalothrix desertica PCC 7102*	19	2	3
filamentous cyanobacterium					
A0A2T1DPM6	Allophycocyanin subunit beta	*filamentous cyanobacterium CCP2*	19	2	3
A0A2T1DPM3	Allophycocyanin	*filamentous cyanobacterium CCP2*	12	2	2
A0A2T1DC75	Allophycocyanin	*filamentous cyanobacterium CCP2*	7	2	3
A0A2T2WBC5	Allophycocyanin	*filamentous cyanobacterium CCP3*	20	2	3
A0A2T2WB84	Allophycocyanin subunit beta	*filamentous cyanobacterium CCP3*	19	2	5
A0A2P8WFG0	Allophycocyanin	*filamentous cyanobacterium CCP5*	20	2	3
A0A2P8VUL2	Allophycocyanin	*filamentous cyanobacterium CCP5*	7	2	3
A0A929A3S2	Allophycocyanin subunit beta	*filamentous cyanobacterium LEGE 07170*	14	2	2
A0A929FFA0	Phycocyanin subunit alpha	*filamentous cyanobacterium LEGE 07170*	10	2	4
A0A929A5X8	Phycocyanin subunit alpha	*filamentous cyanobacterium LEGE 07170*	10	2	4
A0A2T1EZJ2	Allophycocyanin	*filamentous cyanobacterium Phorm 46*	12	2	2
Fischerella major					
A0A1U7H0W4	Allophycocyanin	*Fischerella major NIES-592*	25	3	4
Fischerella muscicola					

A0A2N6K780	Allophycocyanin	*Fischerella muscicola CCMEE 5323*	25	3	4
A0A2N6K004	Allophycocyanin	*Fischerella muscicola CCMEE 5323*	19	2	4
Fischerella thermalis					
A0A2N6KBI8	Allophycocyanin	*Fischerella thermalis CCMEE 5268*	25	3	4
G6FU34	Phycocyanin	*Fischerella thermalis JSC-11*	25	3	4
G6FV62	Phycocyanin	*Fischerella thermalis JSC-11*	19	2	4
Fortiea sp.					
A0A8J7DCH0	Allophycocyanin subunit beta	*Fortiea sp. LEGE XX443*	19	2	6
A0A8J7A5B9	Allophycocyanin subunit alpha	*Fortiea sp. LEGE XX443*	30	3	8
A0A8J7CWD3	Allophycocyanin	*Fortiea sp. LEGE XX443*	19	2	4
Geitlerinema sp.					
A0A1Y5QE20	Phycocyanin alpha subunit	*Geitlerinema sp. H8DM*	10	2	4
Geminocystis sp.					
A0A978T652	Allophycocyanin subunit beta	*Geminocystis sp. M7585_C2015_104*	22	2	5
A0A978T651	Allophycocyanin	*Geminocystis sp. M7585_C2015_104*	15	2	2
A0A0D6ANS0	Allophycocyanin beta chain	*Geminocystis sp. NIES-3709*	30	3	5
Gloeobacter violaceus					
Q7NL80	Allophycocyanin alpha subunit	*Gloeobacter violaceus (strain ATCC 29082 / PCC 7421)*	12	2	2
Gloeobacterales cyanobacterium					
A0A925XSP1	Allophycocyanin	*Gloeobacterales cyanobacterium ES-bin-141*	12	2	2
A0A925ISE1	Allophycocyanin	*Gloeobacterales cyanobacterium ES-bin-313*	12	2	2
Gloeocapsa sp.					
A0A6P2ACY3	Allophycocyanin subunit beta	*Gloeocapsa sp. DLM2.Bin57*	30	3	6
L8LPE1	Allophycocyanin beta subunit apoprotein	*Gloeocapsa sp. PCC 73106*	19	2	4
K9XC24	Allophycocyanin beta subunit apoprotein	*Gloeocapsa sp. PCC 7428*	19	2	4
Gloeocapsopsis dulcis					
A0A6N8G1A9	Allophycocyanin	*Gloeocapsopsis dulcis AAB1*	19	2	3
Gloeocapsopsis sp.					
A0A2G7GIJ4	Allophycocyanin	*Gloeocapsopsis sp. IPPAS B-1203*	25	3	4
Gloeomargarita lithophora					
A0A1J0AG52	Allophycocyanin subunit beta	*Gloeomargarita lithophora Alchichica-D10*	21	2	5
Gloeomargaritaceae cyanobacterium					
A0A930U743	Allophycocyanin subunit beta	*Gloeomargaritaceae cyanobacterium C42_A2020_066*	19	2	4
Gloeomargaritaceae cyanobacterium					
B7KKT0	Allophycocyanin, beta subunit	*Gloeothece citriformis (strain PCC 7424)*	30	3	5
B7KKS9	Phycobilisome protein	*Gloeothece citriformis (strain PCC 7424)*	23	2	3
Gloeothece verrucosa					
E0UA89	Allophycocyanin, beta subunit	*Gloeothece verrucosa (strain PCC 7822)*	30	3	5
E0UA88	Phycocyanin	*Gloeothece verrucosa (strain PCC 7822)*	29	3	4
Gomphosphaeria aponina					
A0A941GQA6	Allophycocyanin subunit beta	*Gomphosphaeria aponina SAG 52.96*	19	2	4
A0A941GXV2	Allophycocyanin subunit alpha	*Gomphosphaeria aponina SAG 52.96*	20	2	2
Halothece sp.					
K9YBF7	Allophycocyanin alpha subunit apoprotein	*Halothece sp. (strain PCC 7418)*	20	2	2
Hassallia byssoidea					

A0A846H2L7	Allophycocyanin	*Hassallia byssoidea VB512170*	25	3	4
A0A846H616	Allophycocyanin	*Hassallia byssoidea VB512170*	19	2	4
Hydrococcus rivularis					
A0A1U7HQ10	Allophycocyanin subunit beta	*Hydrococcus rivularis NIES-593*	39	4	6
Hydrococcus sp.					
A0A968Z758	Allophycocyanin	*Hydrococcus sp. RU_2_2*	23	2	3
Kamptonema sp.					
A0A6P1AC41	Allophycocyanin subunit beta	*Kamptonema sp. SIO1D9*	19	2	3
A0A6P0JYL8	Allophycocyanin subunit beta	*Kamptonema sp. SIO4C4*	17	2	3
Leptolyngbya boryana					
A0AA96WUE2	Allophycocyanin subunit alpha	*Leptolyngbya boryana CZ1*	12	2	2
A0A1Z4JFC6	Allophycocyanin beta subunit	*Leptolyngbya boryana NIES-2135*	25	3	4
Leptolyngbya cf. ectocarpi					
A0A929FCN5	Allophycocyanin subunit alpha	*Leptolyngbya cf. ectocarpi LEGE 11479*	12	2	2
Leptolyngbya foveolarum					
A0A2W4UP22	Allophycocyanin subunit beta	*Leptolyngbya foveolarum*	19	2	3
A0A2W4WL51	Allophycocyanin	*Leptolyngbya foveolarum*	12	2	2
Leptolyngbya sp					
A0A2W7ATZ9	Allophycocyanin	*Leptolyngbya sp*	26	3	3
A0A2W7BV24	Allophycocyanin subunit beta	*Leptolyngbya sp*	19	2	5
A0A2W7A1E1	Allophycocyanin	*Leptolyngbya sp*	20	2	3
A0A2W7B0A4	Allophycocyanin	*Leptolyngbya sp*	7	2	3
A0A2W6ZGK0	Allophycocyanin	*Leptolyngbya sp*	7	2	3
A0A7T5E5H0	Phycocyanin subunit alpha	*Leptolyngbya sp. BL0902*	10	2	4
A0A651EBC1	Allophycocyanin subunit beta	*Leptolyngbya sp. DLM2.Bin15*	19	2	5
A0A651DVI7	Allophycocyanin	*Leptolyngbya sp. DLM2.Bin15*	20	2	2
A0A5Q4DZG5	Allophycocyanin	*Leptolyngbya sp. DLM2.Bin27*	7	2	3
A0A926ZCU8	Allophycocyanin subunit beta	*Leptolyngbya sp. FACHB-16*	14	2	2
A0A926YL87	Phycocyanin subunit alpha	*Leptolyngbya sp. FACHB-321*	11	2	2
A0A926TQ39	Allophycocyanin	*Leptolyngbya sp. FACHB-321*	7	2	3
A0A926YFD0	Allophycocyanin	*Leptolyngbya sp. FACHB-36*	20	2	2
A0A926TEU7	Phycocyanin subunit alpha	*Leptolyngbya sp. FACHB-36*	10	2	9
A0A1Q8ZG86	Phycocyanin subunit alpha	*Leptolyngbya sp. 'hensonii'*	10	2	4
A0A1Q8ZG75	Phycocyanin subunit alpha	*Leptolyngbya sp. 'hensonii'*	10	2	4
A0A1Q8ZG15	Allophycocyanin	*Leptolyngbya sp. 'hensonii'*	26	3	3
A0A6H2NJD9	Allophycocyanin subunit beta	*Leptolyngbya sp. LCM1.Bin17*	19	2	5
A0A6H2NLB3	Phycocyanin subunit alpha	*Leptolyngbya sp. LCM1.Bin17*	10	2	4
A0A0N7Z574	Allophycocyanin alpha chain	*Leptolyngbya sp. NIES-2104*	12	2	2
A0A0S3TXT1	Allophycocyanin alpha subunit	*Leptolyngbya sp. NIES-3755*	12	2	2
A0AA96WDM6	Allophycocyanin subunit beta	*Leptolyngbya sp. NK1-12*	19	2	3
K9Q0W1	Allophycocyanin, beta subunit	*Leptolyngbya sp. PCC 7376*	22	2	4
A0A6G3YTS9	Allophycocyanin subunit beta	*Leptolyngbya sp. SIO1D8*	35	4	6
A0A6I5RE87	Allophycocyanin subunit beta	*Leptolyngbya sp. SIO1E4*	19	2	3
A0A6I5RCW1	Allophycocyanin	*Leptolyngbya sp. SIO1E4*	12	2	2
A0A6I5NF67	Allophycocyanin subunit beta	*Leptolyngbya sp. SIO4C1*	19	2	5
A0A6I5NSQ1	Phycocyanin subunit alpha	*Leptolyngbya sp. SIOISBB*	10	2	4

A0A6I5NL99	Allophycocyanin	*Leptolyngbya sp. SIOISBB*	20	2	3
Leptolyngbyaceae cyanobacterium					
A0A969UWY4	Allophycocyanin subunit beta	*Leptolyngbyaceae cyanobacterium CRU_2_3*	19	2	4
A0A969RBA5	Allophycocyanin subunit beta	*Leptolyngbyaceae cyanobacterium CSU_1_3*	17	2	3
A0A969QMQ5	Allophycocyanin subunit beta	*Leptolyngbyaceae cyanobacterium CSU_1_4*	30	3	5
K8GI72	Phycobilisome protein	*Leptolyngbyaceae cyanobacterium JSC-12*	20	2	2
A0A925BUN4	Allophycocyanin	*Leptolyngbyaceae cyanobacterium LF-bin-113*	20	2	2
A0A978TH61	Allophycocyanin subunit beta	*Leptolyngbyaceae cyanobacterium M65_K2018_010*	19	2	5
A0A968ZW78	Allophycocyanin subunit beta	*Leptolyngbyaceae cyanobacterium RM1_1_2*	19	2	5
A0A9E5RLI8	Allophycocyanin subunit beta	*Leptolyngbyaceae cyanobacterium RM1_406_9*	19	2	5
A0A9E5UWK1	Phycocyanin subunit beta	*Leptolyngbyaceae cyanobacterium RM2_2_4*	12	2	2
A0A969P8E8	Phycocyanin subunit alpha	*Leptolyngbyaceae cyanobacterium RU_5_1*	10	2	4
A0A969GW41	Allophycocyanin subunit beta	*Leptolyngbyaceae cyanobacterium SL_5_14*	19	2	5
A0A968U328	Allophycocyanin	*Leptolyngbyaceae cyanobacterium SM1_4_3*	12	2	2
A0A968PWS3	Phycocyanin subunit beta	*Leptolyngbyaceae cyanobacterium SM1_4_3*	12	2	2
A0A968QC93	Allophycocyanin subunit beta	*Leptolyngbyaceae cyanobacterium SM2_5_2*	19	2	5
A0A968UCT1	Phycocyanin subunit alpha	*Leptolyngbyaceae cyanobacterium SM2_5_2*	10	2	4
A0A930X5L0	Allophycocyanin subunit beta	*Leptolyngbyaceae cyanobacterium T60_A2020_046*	19	2	3
Limnofasciculus baicalensis					
A0AAE3GSN4	Allophycocyanin subunit beta	*Limnofasciculus baicalensis BBK-W-15*	24	3	6
Limnoraphis robusta					
A0A0F5YL91	Allophycocyanin	*Limnoraphis robusta CS-951*	20	2	3
Limnoraphis sp.					
A0A9E4JMK2	Allophycocyanin subunit beta	*Limnoraphis sp. WC205*	22	2	6
Limnothrix rosea					
A0A1Q4R179	Allophycocyanin subunit beta	*Limnothrix rosea IAM M-220*	39	4	7
A0A1Q4R1B7	Allophycocyanin	*Limnothrix rosea IAM M-220*	20	2	3
Limnothrix sp.					
A0A1C0VDV2	Allophycocyanin subunit beta	*Limnothrix sp. P13C2*	22	2	6
A0A969KEW9	Allophycocyanin subunit beta	*Limnothrix sp. RL_2_0*	39	4	7
Lyngbya aestuarii					
U7QGP0	Allophycocyanin, beta subunit	*Lyngbya aestuarii BL J*	22	2	6
Lyngbya sp.					
A0YY95	Allophycocyanin beta subunit	*Lyngbya sp. (strain PCC 8106)*	22	2	6
Mastigocladus laminosus					
P00315	Allophycocyanin alpha chain	*Mastigocladus laminosus*	26	3	4
Merismopedia glauca					
A0A2T1BZ20	Allophycocyanin subunit beta	*Merismopedia glauca CCAP 1448/3*	30	3	7
A0A2T1C7V6	Phycocyanin subunit beta	*Merismopedia glauca CCAP 1448/3*	12	2	2
Merismopedia sp.					
A0A6P0YT94	Phycocyanin subunit alpha	*Merismopedia sp. SIO2A8*	10	2	4
Microchaete diplosiphon					
P08801	Allophycocyanin alpha-B chain	*Microchaete diplosiphon*	25	3	5
Microcoleaceae cyanobacterium					
A0A355CEP6	Allophycocyanin	*Microcoleaceae cyanobacterium UBA10368*	12	2	2
A0A349JQC7	Allophycocyanin	*Microcoleaceae cyanobacterium UBA11344*	12	2	2

Microcoleus sp.					
A0A970AMN8	Allophycocyanin	*Microcoleus sp. CSU_2_2*	12	2	2
A0A926YRL8	Allophycocyanin subunit beta	*Microcoleus sp. FACHB-1515*	19	2	5
A0A926YAQ0	Allophycocyanin	*Microcoleus sp. FACHB-68*	12	2	2
A0A926Y8G0	Allophycocyanin subunit beta	*Microcoleus sp. FACHB-831*	14	2	2
A0A926Y7Z8	Allophycocyanin	*Microcoleus sp. FACHB-831*	12	2	2
A0A926S9B4	Allophycocyanin subunit beta	*Microcoleus sp. FACHB-SPT15*	19	2	5
Microcystis aeruginosa					
A0A510PK91	Phycocyanin subunit beta	*Microcystis aeruginosa 11-30S32*	40	5	6
A0A841ULE8	Allophycocyanin subunit beta	*Microcystis aeruginosa BLCC-F108*	39	4	8
A0A857CZT7	Allophycocyanin subunit beta	*Microcystis aeruginosa FD4*	44	5	7
A0A857D0R4	Phycocyanin subunit alpha	*Microcystis aeruginosa FD4*	20	3	5
A0A857D0J1	Phycocyanin subunit beta	*Microcystis aeruginosa FD4*	40	5	6
A0A966FZR1	Allophycocyanin subunit beta	*Microcystis aeruginosa G11-04*	44	5	9
A0A966L378	Phycocyanin subunit alpha	*Microcystis aeruginosa G11-04*	20	3	5
A0A1V4BS86	Phycocyanin subunit alpha	*Microcystis aeruginosa KW*	10	2	4
A0A552EV89	Phycocyanin subunit beta	*Microcystis aeruginosa Ma_MB_F_20061100_S20D*	40	5	6
A0A552EXM2	Allophycocyanin subunit beta	*Microcystis aeruginosa Ma_MB_S_20031200_S102*	39	4	8
A0A552E7C2	Phycocyanin subunit beta	*Microcystis aeruginosa Ma_MB_S_20031200_S102*	40	5	6
A0A552AN53	Allophycocyanin subunit beta	*Microcystis aeruginosa Ma_OC_H_19870700_S124*	44	5	7
A0A552DNI5	Phycocyanin subunit alpha	*Microcystis aeruginosa Ma_QC_B_20070730_S2*	10	2	4
A0A551YMG3	Phycocyanin subunit alpha	*Microcystis aeruginosa Ma_QC_C_20070703_M131*	10	2	4
A0A552FHF8	Allophycocyanin	*Microcystis aeruginosa Ma_QC_Ca_00000000_S207*	23	2	3
A0A552G5E2	Allophycocyanin subunit beta	*Microcystis aeruginosa Ma_QC_Ch_20071001_S25D*	44	5	7
A0A552G5E8	Allophycocyanin	*Microcystis aeruginosa Ma_QC_Ch_20071001_S25D*	29	3	4
A0A2H6BXJ4	Phycocyanin alpha subunit	*Microcystis aeruginosa NIES-298*	19	2	2
A0A6H9GGD1	Phycocyanin alpha subunit	*Microcystis aeruginosa NIES-3787*	10	2	4
A0A6H9GLU2	Phycocyanin beta subunit	*Microcystis aeruginosa NIES-3804*	40	5	6
A0A0A1W052	Phycocyanin alpha chain	*Microcystis aeruginosa NIES-44*	10	2	4
A0A0A1VYM3	Phycocyanin beta chain	*Microcystis aeruginosa NIES-44*	15	3	3
A0A822LDG6	C-phycocyanin alpha chain	*Microcystis aeruginosa PCC 9432*	10	2	4
I4HAS2	C-phycocyanin beta chain	*Microcystis aeruginosa PCC 9807*	40	5	6
S3KI04	Allophycocyanin beta chain	*Microcystis aeruginosa SPC777*	44	5	7
Microcystis flos-aquae					
A0A552KUX6	Phycocyanin subunit alpha	*Microcystis flos-aquae Mf_QC_C_20070823_S10D*	10	2	4
A0A3E0KYJ4	Phycocyanin subunit beta	*Microcystis flos-aquae TF09*	44	6	7
Microcystis novacekii					
A0A552IP97	Phycocyanin subunit alpha	*Microcystis novacekii Mn_MB_F_20050700_S1D*	10	2	4
Microcystis sp.					
A0A2L2XUH8	Phycocyanin beta chain	*Microcystis sp. 0824*	40	5	6
A0A552ARD9	Allophycocyanin	*Microcystis sp. M_OC_Ca_00000000_C217Col*	35	4	5
A0A2P1UGR1	Phycocyanin subunit beta	*Microcystis sp. MC19*	40	5	6
Microcystis viridis					

A0A3G9JUA6	Allophycocyanin alpha subunit	*Microcystis viridis NIES-102*	29	3	4
A0A3G9JQA9	Phycocyanin alpha subunit	*Microcystis viridis NIES-102*	19	2	2
Microcystis wesenbergii					
A0A552LTQ6	Phycocyanin subunit alpha	*Microcystis wesenbergii Mw_MB_S_20031200_S109D*	10	2	4
A0A552LTQ4	Phycocyanin subunit beta	*Microcystis wesenbergii Mw_MB_S_20031200_S109D*	27	4	4
Microseira wollei					
A0AAV3X7D5	Allophycocyanin alpha subunit	*Microseira wollei NIES-4236*	12	2	2
Mojavia pulchra					
A0A951UFB7	Phycocyanin subunit beta	*Mojavia pulchra JT2-VF2*	14	2	3
Nodosilinea sp.					
A0AA97EGY8	Allophycocyanin subunit alpha-B	*Nodosilinea sp. E11*	7	2	3
A0A8J6W8R7	Allophycocyanin	*Nodosilinea sp. FACHB-13*	7	2	3
A0A8J7EHH2	Allophycocyanin subunit alpha	*Nodosilinea sp. LEGE 06152*	20	2	3
A0A8J7E7L2	Allophycocyanin subunit beta	*Nodosilinea sp. LEGE 07088*	24	3	6
A0A8J7JJ34	Allophycocyanin subunit alpha	*Nodosilinea sp. LEGE 07088*	20	2	2
A0A8J7AZ86	Allophycocyanin	*Nodosilinea sp. LEGE 07298*	7	2	3
Nodularia harveyana					
A0A8J9TPA9	Allophycocyanin alpha chain	*Nodularia harveyana CCAP 1452/1*	19	2	3
Nodularia sp.					
A0A6P1ZYF1	Allophycocyanin	*Nodularia sp.*	19	2	3
A0A218Q2C8	Phycocyanin	*Nodularia sp. NIES-3585*	25	3	4
Nodularia spumigena					
A0A166K5J4	Allophycocyanin	*Nodularia spumigena CENA596*	31	4	5
Nostoc azollae					
D7DY32	Phycocyanin	*Nostoc azollae (strain 0708)*	20	2	3
D7DY33	Allophycocyanin, beta subunit	*Nostoc azollae (strain 0708)*	41	5	10
D7E337	Phycocyanin, alpha subunit	*Nostoc azollae (strain 0708)*	10	2	9
D7E3Q1	Phycocyanin	*Nostoc azollae (strain 0708)*	41	6	10
Nostoc commune					
A0A2R5FJE9	Phycobilisome protein	*Nostoc commune NIES-4072*	32	3	5
Nostoc cycadae					
A0A2H6LMW1	Allophycocyanin subunit alpha apoprotein	*Nostoc cycadae WK-1*	12	2	2
Nostoc edaphicum					
A0A7D7QL00	Allophycocyanin	*Nostoc edaphicum CCNP1411*	17	3	3
A0A7D7LCI8	Allophycocyanin	*Nostoc edaphicum CCNP1411*	19	2	4
Nostoc flagelliforme					
A0A2K8SM82	ApcD, allophycocyanin-B	*Nostoc flagelliforme CCNUN1*	32	3	5
Nostoc linckia					
A0A9Q5ZC51	Allophycocyanin	*Nostoc linckia z8*	32	3	5
Nostoc minutum					
A0A367RWU6	Allophycocyanin	*Nostoc minutum NIES-26*	20	2	3
A0A367RNY7	Allophycocyanin	*Nostoc minutum NIES-26*	23	2	3
Nostoc piscinale					
A0A0M4T6A6	Allophycocyanin	*Nostoc piscinale CENA21*	25	4	7
Nostoc punctiforme					

B2IU44	Phycobilisome protein	*Nostoc punctiforme (strain ATCC 29133 / PCC 73102)*	32	3	5
Nostoc sp.					
K9QZL0	Allophycocyanin alpha subunit apoprotein	*Nostoc sp. (strain ATCC 29411 / PCC 7524)*	30	3	8
K9R0X7	Allophycocyanin beta subunit apoprotein	*Nostoc sp. (strain ATCC 29411 / PCC 7524)*	24	3	7
K9QVA2	Allophycocyanin alpha-B subunit apoprotein	*Nostoc sp. (strain ATCC 29411 / PCC 7524)*	24	3	5
P80556	Allophycocyanin subunit alpha-B	*Nostoc sp. (strain PCC 7120 / SAG 25.82 / UTEX 2576)*	28	3	4
A0A252E1I2	Allophycocyanin	*Nostoc sp. 106C*	19	2	4
A0A367PVT9	Allophycocyanin	*Nostoc sp. ATCC 43529*	17	3	3
A0A367PSG6	Allophycocyanin	*Nostoc sp. ATCC 43529*	19	2	4
A0A2I8A176	Allophycocyanin	*Nostoc sp. CENA543*	25	3	4
A0A926VVH8	Allophycocyanin	*Nostoc sp. FACHB-888*	17	3	3
A0A926VSN9	Allophycocyanin	*Nostoc sp. FACHB-888*	32	3	5
A0A9D7VE10	Allophycocyanin	*Nostoc sp. GBBB01*	12	2	2
A0A9D7VA36	Allophycocyanin	*Nostoc sp. GBBB01*	19	2	4
A0A2Z6CYH8	Phycobilisome protein	*Nostoc sp. HK-01*	32	3	5
A0A938ZJT0	Allophycocyanin	*Nostoc sp. JL23*	32	3	5
A0A939CQQ7	Allophycocyanin	*Nostoc sp. JL34*	19	2	4
A0A1E2WL85	Allophycocyanin	*Nostoc sp. KVJ20*	17	3	3
A0A1E2WP45	Allophycocyanin	*Nostoc sp. KVJ20*	19	2	4
A0A2L2NUU7	Allophycocyanin subunit alpha	*Nostoc sp. 'Lobaria pulmonaria (5183) cyanobiont'*	12	2	2
A0A2L2NN49	Phycobilisome protein	*Nostoc sp. 'Lobaria pulmonaria (5183) cyanobiont'*	22	2	3
A0A939IF90	Allophycocyanin	*Nostoc sp. LPT*	12	2	2
A0A939CXE4	Allophycocyanin	*Nostoc sp. LPT*	22	2	3
A0A1C0VME8	Allophycocyanin subunit beta	*Nostoc sp. MBR 210*	24	3	7
A0A1C0VMD8	Allophycocyanin	*Nostoc sp. MBR 210*	36	4	9
A0A1C0UTT9	Allophycocyanin	*Nostoc sp. MBR 210*	25	4	7
A0A1Z4IDX0	Phycobilisome protein	*Nostoc sp. NIES-2111*	12	2	2
A0A1Z4I4E1	Phycobilisome protein	*Nostoc sp. NIES-2111*	22	2	3
K9QEP5	Allophycocyanin alpha-B subunit apoprotein	*Nostoc sp. PCC 7107*	32	3	5
A0A2D0HKN0	Allophycocyanin	*Nostoc sp. 'Peltigera malacea cyanobiont' DB3992*	12	2	2
A0A2D0HLN0	Allophycocyanin	*Nostoc sp. 'Peltigera malacea cyanobiont' DB3992*	32	3	5
A0A235IB78	Allophycocyanin	*Nostoc sp. 'Peltigera membranacea cyanobiont' 210A*	12	2	2
A0A235IB31	Phycocyanin subunit alpha	*Nostoc sp. 'Peltigera membranacea cyanobiont' 210A*	10	2	4
A0A235J6M6	Allophycocyanin	*Nostoc sp. 'Peltigera membranacea cyanobiont' 232*	12	2	2
A0A2L2NAQ3	Allopycocyanin subunit alpha	*Nostoc sp. 'Peltigera membranacea cyanobiont' N6*	12	2	2
A0A2L2NGF2	Allophycocyanin subunit alpha	*Nostoc sp. 'Peltigera membranacea cyanobiont' N6*	19	2	4
A0A252ECW7	Allophycocyanin	*Nostoc sp. T09*	19	2	4
A0A841VJM5	Allophycocyanin	*Nostoc sp. UCD120*	12	2	2
A0A4Y5PW22	Allophycocyanin alpha	*Nostoc sp. WR13*	36	4	9
A0A4Y5PW23	Allophycocyanin beta	*Nostoc sp. WR13*	24	3	7
Nostoc sphaeroides					
A0A5P8W6Q0	ApcA, allophycocyanin alpha subunit	*Nostoc sphaeroides CCNUC1*	17	3	3

A0A5P8VZW4	ApcD, allophycocyanin-B	*Nostoc sphaeroides CCNUC1*	32	3	5
Nostocaceae cyanobacterium					
A0A838VMI0	Allophycocyanin subunit beta	*Nostocaceae cyanobacterium*	19	2	6
A0A838VQ68	Allophycocyanin	*Nostocaceae cyanobacterium*	25	4	7
Nostocales cyanobacterium					
A0A9E6RYN0	Allophycocyanin	*Nostocales cyanobacterium*	35	4	9
A0A9E6RXB7	Allophycocyanin subunit beta	*Nostocales cyanobacterium*	16	2	5
A0A9E6RWB5	Allophycocyanin	*Nostocales cyanobacterium*	32	5	8
A0A1Y0RS29	Allophycocyanin	*Nostocales cyanobacterium HT-58-2*	26	3	3
A0A928WCI3	Allophycocyanin subunit alpha	*Nostocales cyanobacterium LEGE 11386*	19	2	3
A0A928WCS4	Allophycocyanin	*Nostocales cyanobacterium LEGE 11386*	23	2	3
Oculatella sp.					
A0A8J7JSZ9	Allophycocyanin subunit beta	*Oculatella sp. LEGE 06141*	19	2	5
A0A8J7EJZ7	Allophycocyanin subunit alpha	*Oculatella sp. LEGE 06141*	20	2	3
A0A8J7JX08	Phycocyanin subunit alpha	*Oculatella sp. LEGE 06141*	10	2	9
Oscillatoria acuminata					
K9TDG1	Allophycocyanin alpha subunit apoprotein	*Oscillatoria acuminata PCC 6304*	20	2	2
Oscillatoria nigro-viridis					
A0A8J9XFI1	Allophycocyanin alpha chain	*Oscillatoria nigro-viridis*	26	3	3
Oscillatoria sp.					
A0A926X3L2	Allophycocyanin	*Oscillatoria sp. FACHB-1407*	20	2	3
Oscillatoriales cyanobacterium					
A0A1J5GIY8	Allophycocyanin subunit beta	*Oscillatoriales cyanobacterium CG2_30_44_21*	32	3	8
A0A1J5GL20	Allophycocyanin	*Oscillatoriales cyanobacterium CG2_30_44_21*	25	3	3
A0A968IZQ6	Allophycocyanin	*Oscillatoriales cyanobacterium SM2_1_8*	12	2	2
A0A968M204	Phycocyanin subunit alpha	*Oscillatoriales cyanobacterium SM2_1_8*	10	2	4
A0A968HQM9	Allophycocyanin subunit beta	*Oscillatoriales cyanobacterium SM2_2_1*	27	3	5
A0A1C0VYM6	Allophycocyanin	*Oscillatoriales cyanobacterium USR001*	12	2	2
Pannus brasiliensis					
A0AAW9QT48	Allophycocyanin subunit alpha	*Pannus brasiliensis CCIBt3594*	20	2	3
A0AAW9QFW6	Allophycocyanin subunit beta	*Pannus brasiliensis CCIBt3594*	30	3	5
Pantanalinema sp.					
A0A937EXK1	Allophycocyanin	*Pantanalinema sp. GBBB05*	20	2	2
A0A937EV79	Allophycocyanin	*Pantanalinema sp. GBBB05*	7	2	3
Parathermosynechococcus lividus					
A0A2D2Q321	Allophycocyanin	*Parathermosynechococcus lividus PCC 6715*	12	2	2
A0A2D2Q3N1	C-phycocyanin beta subunit	*Parathermosynechococcus lividus PCC 6715*	12	2	2
Pegethrix bostrychoides					
A0A951U595	Allophycocyanin subunit beta	*Pegethrix bostrychoides GSE-TBD4-15B*	19	2	4
A0A951U6B3	Phycocyanin subunit alpha	*Pegethrix bostrychoides GSE-TBD4-15B*	10	2	4
Pelatocladus maniniholoensis					
A0A9E3HAM7	Allophycocyanin subunit alpha	*Pelatocladus maniniholoensis HA4357-MV3*	26	3	3
Phormidium ambiguum					
A0A1U7IEE7	Allophycocyanin subunit beta	*Phormidium ambiguum IAM M-71*	19	2	5
A0A1U7IE87	Allophycocyanin	*Phormidium ambiguum IAM M-71*	12	2	2
Phormidium sp.					

A0A0P7ZUX2	Allophycocyanin, beta subunit	*Phormidium sp. OSCR*	28	3	5
A0A522XEV7	Allophycocyanin subunit beta	*Phormidium sp. SL48-SHIP*	28	3	5
Phormidium tenue					
A0A1U7JAT3	Allophycocyanin	*Phormidium tenue NIES-30*	20	2	3
Picosynechococcus sp.					
A0AAE8LPP7	Allophycocyanin alpha subunit apoprotein	*Picosynechococcus sp. OG1*	20	2	3
Planktothricoides raciborskii					
A0AAU8JH72	Allophycocyanin subunit beta	*Planktothricoides raciborskii GIHE-MW2*	19	2	4
Planktothricoides sp.					
A0A7C3VLG6	Allophycocyanin subunit beta	*Planktothricoides sp. SpSt-374*	19	2	4
A0A0M1JPI9	Allophycocyanin	*Planktothricoides sp. SR001*	20	2	2
Planktothrix paucivesiculata					
A0A7Z9BJ54	Allophycocyanin beta chain	*Planktothrix paucivesiculata PCC 9631*	30	3	5
Planktothrix pseudagardhii					
A0A9W4G280	Allophycocyanin alpha chain	*Planktothrix pseudagardhii*	20	2	3
Planktothrix serta					
A0A7Z9BR97	Allophycocyanin beta chain	*Planktothrix serta PCC 8927*	30	3	7
A0A7Z9BR96	Allophycocyanin alpha chain	*Planktothrix serta PCC 8927*	20	2	3
Planktothrix sp.					
A0A3C1RUJ6	Allophycocyanin subunit beta	*Planktothrix sp. UBA8407*	30	3	5
Planktothrix tepida					
A0A1J1LN43	Allophycocyanin beta chain	*Planktothrix tepida PCC 9214*	30	3	7
Plectonema cf. radiosum					
A0A8J7K299	Phycocyanin subunit alpha	*Plectonema cf. radiosum LEGE 06105*	10	2	9
Pleurocapsa sp.					
A0A969NNN1	Allophycocyanin subunit beta	*Pleurocapsa sp. CRU_1_2*	19	2	3
K9TAS6	Allophycocyanin alpha subunit apoprotein	*Pleurocapsa sp. PCC 7327*	29	3	4
A0A968IRB4	Allophycocyanin	*Pleurocapsa sp. SU_5_0*	12	2	2
Pleurocapsales cyanobacterium					
A0A929FTT8	Allophycocyanin subunit beta	*Pleurocapsales cyanobacterium LEGE 06147*	30	3	5
A0A929FWD4	Allophycocyanin	*Pleurocapsales cyanobacterium LEGE 06147*	20	2	3
A0A928ZDL8	Allophycocyanin subunit beta	*Pleurocapsales cyanobacterium LEGE 10410*	19	2	3
Pseudanabaena catenata					
A0A9X4RIW4	Allophycocyanin subunit beta	*Pseudanabaena catenata USMAC16*	27	3	5
Pseudanabaena cinerea					
A0A926UST9	Allophycocyanin subunit beta	*Pseudanabaena cinerea FACHB-1277*	38	4	9
A0A926UTP6	Allophycocyanin	*Pseudanabaena cinerea FACHB-1277*	19	2	2
Pseudanabaena frigida					
A0A2W4W8I7	Allophycocyanin subunit beta	*Pseudanabaena frigida*	32	3	8
A0A2W4Y8U6	Allophycocyanin	*Pseudanabaena frigida*	25	3	3
Pseudanabaena sp					
A0A2W7AAG3	Allophycocyanin subunit beta	*Pseudanabaena sp*	32	3	8
A0A2W6ZMB9	Allophycocyanin subunit beta	*Pseudanabaena sp*	32	3	8
A0A352JCX6	Allophycocyanin	*Pseudanabaena sp*	12	2	2
A0A2W6Z5S6	Allophycocyanin	*Pseudanabaena sp*	25	3	3
A0A2W7C4P5	Allophycocyanin	*Pseudanabaena sp*	25	3	3

A0A352JCX7	Allophycocyanin subunit beta	*Pseudanabaena sp*	22	2	4
A0A2Z5X379	Allophycocyanin subunit beta	*Pseudanabaena sp. ABRG5-3*	22	2	4
A0A2Z5X2N9	Allophycocyanin alpha subunit	*Pseudanabaena sp. ABRG5-3*	12	2	2
A0A970B1A0	Phycocyanin subunit alpha	*Pseudanabaena sp. CRU_2_10*	10	2	4
A0A926WAZ3	Allophycocyanin subunit beta	*Pseudanabaena sp. FACHB-2040*	19	2	5
V5NXK8	ApcB	*Pseudanabaena sp. lw0831*	32	3	8
K9SNR5	Allophycocyanin beta subunit apoprotein	*Pseudanabaena sp. PCC 7367*	35	4	8
A0A256B7Q9	Allophycocyanin subunit beta	*Pseudanabaena sp. SR411*	32	3	8
A0A968Q0N3	Allophycocyanin subunit beta	*Pseudanabaena sp. SU_2_4*	36	4	6
A0A6C2DZJ7	Allophycocyanin	*Pseudanabaena sp. UWO310*	25	3	3
A0A652Z030	Allophycocyanin subunit beta	*Pseudanabaena sp. UWO311*	32	3	8
A0A652Z043	Allophycocyanin	*Pseudanabaena sp. UWO311*	25	3	3
Pseudanabaenaceae cyanobacterium					
A0A928VES5	Allophycocyanin subunit alpha	*Pseudanabaenaceae cyanobacterium LEGE 13415*	12	2	2
Pseudocalidococcus azoricus					
A0AAE4FQ17	Allophycocyanin subunit alpha	*Pseudocalidococcus azoricus BACA0444*	12	2	2
A0AAE4FUT5	Phycocyanin subunit beta	*Pseudocalidococcus azoricus BACA0444*	12	2	2
Richelia sinica					
A0A975T907	Allophycocyanin alpha chain	*Richelia sinica FACHB-800*	31	3	8
A0A975Y5K5	Phycocyanin	*Richelia sinica FACHB-800*	32	5	8
Richelia sp.					
A0A969QCR1	Allophycocyanin	*Richelia sp. CSU_2_1*	12	2	2
A0A969AE68	Allophycocyanin subunit beta	*Richelia sp. RM1_1_1*	19	2	6
A0A969MJ06	Allophycocyanin	*Richelia sp. RM2_1_2*	19	2	3
A0A968WAI6	Phycocyanin subunit alpha	*Richelia sp. SM1_7_0*	10	2	9
Rippkaea orientalis					
B7K5Q5	Allophycocyanin, beta subunit	*Rippkaea orientalis (strain PCC 8801 / RF-1)*	30	3	7
B7JX68	Phycocyanin, alpha subunit	*Rippkaea orientalis (strain PCC 8801 / RF-1)*	10	2	4
Rivularia sp.					
A0A949THS9	Allophycocyanin subunit beta	*Rivularia sp.*	19	2	6
A0A949TLI6	Phycocyanin subunit alpha	*Rivularia sp.*	10	2	9
A0A949T6V0	Allophycocyanin subunit alpha	*Rivularia sp. MS3*	25	3	4
K9RFJ2	Allophycocyanin beta subunit apoprotein	*Rivularia sp. PCC 7116*	19	2	6
K9REV4	Phycocyanin, alpha subunit	*Rivularia sp. PCC 7116*	10	2	9
Scytonema hofmannii					
A0A139WXF7	Allophycocyanin	*Scytonema hofmannii PCC 7110*	25	3	4
Scytonema sp.					
A0AA91GVQ4	Allophycocyanin	*Scytonema sp. HK-05*	17	3	3
A0AA91GS82	Allophycocyanin	*Scytonema sp. HK-05*	23	2	3
A0A969CK74	Allophycocyanin	*Scytonema sp. RU_4_4*	19	2	3
A0A968YRH4	Phycocyanin subunit alpha	*Scytonema sp. RU_4_4*	10	2	4
A0A969CLK2	Phycocyanin subunit alpha	*Scytonema sp. RU_4_4*	10	2	4
A0A844MPL2	Allophycocyanin	*Scytonema sp. UIC 10036*	19	2	3
A0A844M9P8	Allophycocyanin	*Scytonema sp. UIC 10036*	19	2	4
Snowella sp					

A0A2W7BUA5	Allophycocyanin subunit beta	*Snowella sp*	22	2	4
Sphaerospermopsis reniformis					
A0A479ZWB6	Phycocyanin subunit alpha	*Sphaerospermopsis reniformis*	26	3	11
A0A480A8R2	Phycocyanin	*Sphaerospermopsis reniformis*	16	2	6
A0A479ZXB4	Phycobilisome protein	*Sphaerospermopsis reniformis*	32	5	8
A0A479ZSX1	Phycocyanin, beta subunit	*Sphaerospermopsis reniformis*	27	4	9
Sphaerospermopsis sp.					
A0A846EIF2	Allophycocyanin	*Sphaerospermopsis sp. SIO1G1*	25	3	4
A0A846EGU6	Allophycocyanin	*Sphaerospermopsis sp. SIO1G1*	22	4	6
Spirulina major					
A0A8J9SZF1	Allophycocyanin beta chain	*Spirulina major*	19	2	3
Spirulinaceae cyanobacterium					
A0A968T7X6	Allophycocyanin subunit beta	*Spirulinaceae cyanobacterium SM2_1_0*	39	4	8
Stanieria cyanosphaera					
K9XS05	Allophycocyanin beta subunit apoprotein	*Stanieria cyanosphaera (strain ATCC 29371 / PCC 7437)*	30	3	5
K9XWL4	Phycocyanin, beta subunit	*Stanieria cyanosphaera (strain ATCC 29371 / PCC 7437)*	12	2	2
Stanieria sp					
A0A140KB32	Phycobilisome protein	*Stanieria sp. NIES-3757*	20	2	3
Stenomitos frigidus					
A0A2T1DX81	Allophycocyanin	*Stenomitos frigidus ULC18*	12	2	2
A0A2T1E6C8	Allophycocyanin	*Stenomitos frigidus ULC18*	7	2	3
Symplocastrum torsivum					
A0A951UD14	Allophycocyanin subunit beta	*Symplocastrum torsivum CPER-KK1*	19	2	5
Synechococcaceae bacterium					
A0A966T0S8	Phycocyanin subunit alpha	*Synechococcaceae bacterium WBB_3_034*	10	2	4
Synechococcaceae cyanobacterium					
A0A969KIN5	Allophycocyanin subunit beta	*Synechococcaceae cyanobacterium RL_1_2*	22	2	6
A0A968VDE0	Allophycocyanin subunit beta	*Synechococcaceae cyanobacterium SM2_3_2*	27	3	5
Synechococcales cyanobacterium					
A0A966B0S7	Phycocyanin subunit alpha	*Synechococcales cyanobacterium H12SWP_bin.12*	10	2	4
A0A930U2A6	Allophycocyanin subunit alpha	*Synechococcales cyanobacterium K44_A2020_017*	20	2	2
A0A978U6M1	Allophycocyanin subunit beta	*Synechococcales cyanobacterium M55_K2018_004*	19	2	3
A0A969DZ07	Phycocyanin subunit alpha	*Synechococcales cyanobacterium RM1_1_8*	10	2	4
A0A969DSH7	Allophycocyanin subunit beta	*Synechococcales cyanobacterium RM1_1_8*	19	2	4
A0A930TT59	Allophycocyanin subunit beta	*Synechococcales cyanobacterium T60_A2020_003*	30	3	7
A0A930TPG9	Allophycocyanin subunit alpha	*Synechococcales cyanobacterium T60_A2020_003*	20	2	3
Synechococcus lacustris					
A0A2P7EIB9	Phycocyanin subunit beta	*Synechococcus lacustris str. Tous*	12	2	2
Synechococcus sp					
Q76N39	Alpha-phycocyanin	*Synechococcus sp*	11	2	2
Q5N4S9	C-phycocyanin-2 alpha subunit	*Synechococcus sp. (strain ATCC 27144 / PCC 6301 / SAUG 1402/1)*	11	2	2
K9RWE6	Phycocyanin, beta subunit	*Synechococcus sp. (strain ATCC 27167 / PCC 6312)*	12	2	2
B4WI76	Allophycocyanin, beta subunit	*Synechococcus sp. (strain ATCC 29403 / PCC 7335)*	19	2	3

Q05ZB1	Phycocyanin, alpha subunit	*Synechococcus sp. BL107*	10	2	4
A0A937HWM7	Phycocyanin subunit alpha	*Synechococcus sp. BS307-5m-G38*	10	2	4
A0A1Q2TZY0	Allophycocyanin, beta subunit	*Synechococcus sp. NIES-970*	39	4	7
A0A1Q2U063	Allophycocyanin alpha subunit	*Synechococcus sp. NIES-970*	20	2	3
K9SUP9	Allophycocyanin beta subunit apoprotein	*Synechococcus sp. PCC 7502*	27	3	5
A0A024CHA8	R-phycocyanin II, alpha subunit	*uncultured Synechococcus sp*	10	2	4
A0A024CH50	R-phycocyanin II, alpha subunit	*uncultured Synechococcus sp*	10	2	4
A0A024CHA6	R-phycocyanin II, alpha subunit	*uncultured Synechococcus sp*	10	2	4
Synechocystis sp.					
A0A068N0S6	Allophycocyanin beta chain	*Synechocystis sp. (strain PCC 6714)*	40	4	8
A0AA50DXE6	Phycocyanin subunit beta	*Synechocystis sp. B12*	14	2	2
Thermoleptolyngbya oregonensis					
A0AA97BC92	Allophycocyanin subunit beta	*Thermoleptolyngbya oregonensis NK1-22*	19	2	5
A0AA96Y7U5	Allophycocyanin	*Thermoleptolyngbya oregonensis NK1-22*	20	2	2
Thermostichus vulcanus					
B3VNK3	Allophycocyanin beta subunit	*Thermostichus vulcanus str. Copeland*	19	2	3
Thermosynechococcus sp					
A0A915V2U9	C-phycocyanin beta chain	*Thermosynechococcus sp*	12	2	2
A0A5C2M414	Allophycocyanin subunit beta	*Thermosynechococcus sp. CL-1*	19	2	3
A0A5C2M6M9	Allophycocyanin	*Thermosynechococcus sp. CL-1*	12	2	2
Thermosynechococcus vestitus					
P50030	Allophycocyanin alpha chain	*Thermosynechococcus vestitus (strain NIES-2133 / IAM M-273 / BP-1)*	12	2	2
Tolypothrix bouteillei					
A0A0C1QT48	Allophycocyanin	*Tolypothrix bouteillei VB521301*	25	3	4
A0A0C1RG94	Allophycocyanin	*Tolypothrix bouteillei VB521301*	19	2	4
Tolypothrix sp.					
A0A218QHG9	Phycobilisome protein	*Tolypothrix sp. NIES-4075*	25	3	4
A0A218QEF0	Phycobilisome protein	*Tolypothrix sp. NIES-4075*	19	2	4
A0A6G9SDK2	Allophycocyanin	*Tolypothrix sp. PCC 7910*	25	3	5
Trichocoleus sp.					
A0A8J6V3R5	Allophycocyanin	*Trichocoleus sp. FACHB-832*	19	2	4
A0A8J6RWQ1	Allophycocyanin	*Trichocoleus sp. FACHB-90*	19	2	4
Trichormus variabilis					
Q3M9V1	Phycobilisome protein	*Trichormus variabilis (strain ATCC 29413 / PCC 7937)*	25	3	4
A0A3S1CEI7	Allophycocyanin alpha chain	*Trichormus variabilis SAG 1403-4b*	47	6	11
A0A433V1P0	Allophycocyanin beta chain	*Trichormus variabilis SAG 1403-4b*	24	3	7
A0A433USV6	Allophycocyanin-B	*Trichormus variabilis SAG 1403-4b*	34	4	7
Tumidithrix elongata					
A0AAW9PVD1	Allophycocyanin subunit beta	*Tumidithrix elongata BACA0141*	27	3	7
Tychonema bourrellyi					
A0A2G4EVV9	Allophycocyanin	*Tychonema bourrellyi FEM_GT703*	12	2	2
Umezakia ovalisporum					
A0AA43GWQ4	Allophycocyanin subunit beta	*Umezakia ovalisporum FSS-62*	24	3	5
A0AA43GX24	Allophycocyanin subunit alpha	*Umezakia ovalisporum FSS-62*	19	2	3
Woronichinia naegeliana					

A0A977L2D6	Allophycocyanin	*Woronichinia naegeliana WA131*	23	2	3
A0A977L226	Phycocyanin subunit alpha	*Woronichinia naegeliana WA131*	26	2	3
A0A977PZ20	Allophycocyanin subunit beta	*Woronichinia naegeliana WA131*	40	4	7
A0A977L2H6	Phycocyanin subunit beta	*Woronichinia naegeliana WA131*	51	6	7

Table 5: Proteins from cyanobacterial strains detected by trypsin digestion followed by LC-MS/MS analysis from lake water 6.

Accession	Description	Species	Coverage [%]	# Peptides	# PSMs
Alkalinema sp.					
A0A251WJA0	Allophycocyanin	*Alkalinema sp. CACIAM 70d*	12	2	2
Anabaena cylindrica					
P07325	Allophycocyanin alpha chain	*Anabaena cylindrica*	17	2	3
Anabaena sp.					
K7WMD9	Phycobilisome protein ApcA	*Anabaena sp. 90*	28	4	5
K7WTL4	Phycocyanin beta subunit	*Anabaena sp. 90*	28	5	7
A0A1B7WMN4	Phycocyanin	*Anabaena sp. CRKS33*	19	2	2
A0A1B7WSR8	Allophycocyanin	*Anabaena sp. CRKS33*	22	3	4
A0A926ULC4	Allophycocyanin	*Anabaena sp. FACHB-1237*	17	2	3
A0A1B7V0A1	Allophycocyanin	*Anabaena sp. LE011-02*	28	4	5
A0A9X1GP51	Allophycocyanin	*Anabaena sp. PCC 7938*	17	2	3
A0A3D4C397	Phycocyanin subunit alpha	*Anabaena sp. UBA12330*	19	2	2
A0A455KZV8	Phycocyanin B	*uncultured Anabaena sp*	20	4	6
Anabaena sphaerica					
A0A927A4K0	Allophycocyanin	*Anabaena sphaerica FACHB-251*	17	2	3
A0A927A4I2	Phycocyanin subunit alpha	*Anabaena sphaerica FACHB-251*	19	2	2
A0A927A3U9	Phycocyanin subunit beta	*Anabaena sphaerica FACHB-251*	12	2	2
Anabaenopsis circularis					
A0A1Z4GAW8	Phycocyanin beta subunit	*Anabaenopsis circularis NIES-21*	12	2	2
Anabaenopsis elenkinii					
A0A7S6RDD3	Phycocyanin subunit beta	*Anabaenopsis elenkinii CCIBt3563*	16	3	3
Aphanizomenon flos-aquae					
A0A1B7WBM0	Phycocyanin	*Aphanizomenon flos-aquae WA102*	19	2	2
A0A1B7X4P2	Allophycocyanin	*Aphanizomenon flos-aquae WA102*	17	2	3
A0A1B7WZK6	Phycocyanin	*Aphanizomenon flos-aquae WA102*	24	4	6
Aphanizomenon sp.					
A0A844ICS2	Phycocyanin subunit beta	*Aphanizomenon sp. UHCC 0183*	12	2	2
Aphanothece hegewaldii					
A0A2T1LW89	Allophycocyanin	*Aphanothece hegewaldii CCALA 016*	12	2	2
Aphanothece sacrum					
A0A401ICC4	Allophycocyanin subunit alpha apoprotein	*Aphanothece sacrum FPU1*	12	2	2
Calothrix sp.					
A0A1Z4NJH9	Phycocyanin	*Calothrix sp. NIES-3974*	17	2	2
K9PL05	Phycocyanin, beta subunit	*Calothrix sp. PCC 7507*	12	2	2
Chamaesiphon minutus					
K9ULF3	Phycocyanin, beta subunit	*Chamaesiphon minutus (strain ATCC 27169 / PCC 6605)*	8	2	2
Cronbergia siamensis					
B5A5J8	Phycocyanin beta subunit	*Cronbergia siamensis TISTR 8012*	12	2	2
Cuspidothrix issatschenkoi					
A0A2S6CQK9	Phycocyanin subunit beta	*Cuspidothrix issatschenkoi CHARLIE-1*	24	4	6
Cyanobacteria bacterium					
A0A3M1L8Y9	Phycocyanin subunit beta	*Cyanobacteria bacterium J083*	12	2	2
A0A3B8K348	Phycocyanin subunit alpha	*Cyanobacteria bacterium UBA8553*	19	2	2

Cyanobacterium aponinum					
A0AAF1C585	Phycocyanin subunit beta	*Cyanobacterium aponinum AL20115*	12	2	2
Cyanobacterium sp.					
A0A2K8WQM9	Phycocyanin beta subunit CpcB	*Cyanobacterium sp. HL-69*	12	2	2
A0A930XCN1	Phycocyanin subunit beta	*Cyanobacterium sp. T60_A2020_053*	12	2	2
Cyanomargarita calcarea					
A0A951QUE8	Phycocyanin subunit beta	*Cyanomargarita calcarea GSE-NOS-MK-12-04C*	12	2	2
Cylindrospermopsis raciborskii					
A0A9Q5QUG8	Allophycocyanin	*Cylindrospermopsis raciborskii CENA302*	17	2	2
A0A838WGC8	Phycocyanin subunit beta	*Cylindrospermopsis raciborskii CS-506_A*	10	2	2
Dendronalium phyllosphericum					
A0A8J7LG09	Phycocyanin subunit beta	*Dendronalium phyllosphericum CENA369*	8	2	2
Desertifilum tharense					
A0A1E5QEK5	Phycocyanin subunit beta	*Desertifilum tharense IPPAS B-1220*	8	2	2
Desmonostoc muscorum					
A0A8J6ZVU6	Allophycocyanin subunit alpha	*Desmonostoc muscorum LEGE 12446*	12	2	3
Dolichospermum compactum					
A0A1Z4VAR2	Phycocyanin alpha subunit	*Dolichospermum compactum NIES-806*	19	2	2
A0A1Z4V5K5	Phycocyanin	*Dolichospermum compactum NIES-806*	22	3	4
A0A1Z4VAT7	Phycocyanin beta subunit	*Dolichospermum compactum NIES-806*	12	2	2
Dolichospermum flos-aquae					
A0A6H2C1Q0	Phycocyanin subunit alpha	*Dolichospermum flos-aquae CCAP 1403/13F*	19	2	2
Dolichospermum sp.					
A0AAW6JTW9	Phycocyanin subunit alpha	*Dolichospermum sp. ST_sed8*	19	2	2
A0AAW6JT81	Allophycocyanin subunit alpha	*Dolichospermum sp. ST_sed8*	22	3	4
A0A5C0DQG9	C-phycocyanin alpha chain	*Dolichospermum sp. UHCC 0315A*	19	2	2
A0A5C0DNG5	C-phycocyanin beta chain	*Dolichospermum sp. UHCC 0315A*	20	3	5
Fortiea sp.					
A0A8J7D810	Phycocyanin subunit beta	*Fortiea sp. LEGE XX443*	12	2	2
Geminocystis sp.					
A0A978T651	Allophycocyanin	*Geminocystis sp. M7585_C2015_104*	12	2	3
A0A0D6ANR5	Allophycocyanin alpha chain	*Geminocystis sp. NIES-3709*	12	2	2
Gloeothece verrucosa					
E0UA88	Phycocyanin	*Gloeothece verrucosa (strain PCC 7822)*	12	2	3
Leptolyngbya boryana					
A0AA96WUE2	Allophycocyanin subunit alpha	*Leptolyngbya boryana CZ1*	12	2	2
Leptolyngbya sp.					
U9VUN9	C-phycocyanin beta chain	*Leptolyngbya sp. Heron Island J*	12	2	2
A0A6I5N4Q9	Phycocyanin subunit beta	*Leptolyngbya sp. SIO4C1*	12	2	2
Leptolyngbyaceae cyanobacterium					
A0A969RYG1	Phycocyanin subunit beta	*Leptolyngbyaceae cyanobacterium CRU_2_3*	8	2	2
Microcystis flos-aquae					
A0A3E0KYJ4	Phycocyanin subunit beta	*Microcystis flos-aquae TF09*	16	2	2
Microcystis sp.					
A0A552ARD9	Allophycocyanin	*Microcystis sp. M_OC_Ca_00000000_C217Col*	12	2	2
Microcystis viridis					

A0A3G9JUA6	Allophycocyanin alpha subunit	*Microcystis viridis NIES-102*	12	2	2
Mojavia pulchra					
A0A951UFB7	Phycocyanin subunit beta	*Mojavia pulchra JT2-VF2*	16	3	3
Nodularia harveyana					
A0A8J9T0Y8	C-phycocyanin beta chain	*Nodularia harveyana CCAP 1452/1*	8	2	2
Nodularia sp.					
A0A6P1ZW31	Phycocyanin subunit beta	*Nodularia sp.*	12	2	2
A0A218Q3W6	Phycocyanin beta subunit	*Nodularia sp. NIES-3585*	8	2	2
Nodularia spumigena					
A0A161UQG9	Phycocyanin subunit beta	*Nodularia spumigena CENA596*	12	2	2
Nostoc azollae					
D7E337	Phycocyanin, alpha subunit	*Nostoc azollae (strain 0708)*	19	2	2
Nostoc piscinale					
A0A0M3V570	Phycocyanin	*Nostoc piscinale CENA21*	12	2	2
Nostoc sp.					
K9QXY7	Phycocyanin, beta subunit	*Nostoc sp. (strain ATCC 29411 / PCC 7524)*	12	2	2
A0A252E6C1	Phycocyanin subunit beta	*Nostoc sp. 106C*	12	2	2
A0A1C0VMD8	Allophycocyanin	*Nostoc sp. MBR 210*	17	2	2
A0A1C0UYI3	Phycocyanin subunit beta	*Nostoc sp. MBR 210*	12	2	2
A0A1Z4I1T5	Phycocyanin beta subunit	*Nostoc sp. NIES-2111*	12	2	2
A0A252DGI8	Phycocyanin subunit beta	*Nostoc sp. T09*	12	2	2
A0A4Y5PW22	Allophycocyanin alpha	*Nostoc sp. WR13*	17	2	2
Nostocaceae cyanobacterium					
A0A838VMA7	Phycocyanin subunit beta	*Nostocaceae cyanobacterium*	12	2	2
Nostocales cyanobacterium					
A0A928WKW8	Phycocyanin subunit beta	*Nostocales cyanobacterium LEGE 11386*	12	2	2
Pannus brasiliensis					
A0AAW9QT48	Allophycocyanin subunit alpha	*Pannus brasiliensis CCIBt3594*	12	2	2
Phormidesmis sp.					
A0A969D556	Phycocyanin subunit beta	*Phormidesmis sp. RL_2_1*	8	2	2
Planktothrix pseudagardhii					
A0A9W4G280	Allophycocyanin alpha chain	*Planktothrix pseudagardhii*	12	2	2
Planktothrix serta					
A0A7Z9BR96	Allophycocyanin alpha chain	*Planktothrix serta PCC 8927*	12	2	2
Plectonema cf. radiosum					
A0A8J7K299	Phycocyanin subunit alpha	*Plectonema cf. radiosum LEGE 06105*	19	2	2
Pleurocapsa sp.					
K9TAS6	Allophycocyanin alpha subunit apoprotein	*Pleurocapsa sp. PCC 7327*	12	2	2
Pseudanabaena sp.					
A0A2Z5WZ42	Phycocyanin, beta subunit	*Pseudanabaena sp. ABRG5-3*	8	2	2
Pseudocalidococcus azoricus					
A0AAE4FUT5	Phycocyanin subunit beta	*Pseudocalidococcus azoricus BACA0444*	8	2	2
Richelia sinica					
A0A975T7E6	Phycocyanin beta subunit	*Richelia sinica FACHB-800*	12	2	2
Richelia sp.					
A0A968WAI6	Phycocyanin subunit alpha	*Richelia sp. SM1_7_0*	19	2	2

Rivularia sp.					
A0A949TLI6	Phycocyanin subunit alpha	*Rivularia sp.*	19	2	2
K9REV4	Phycocyanin, alpha subunit	*Rivularia sp. PCC 7116*	19	2	2
Scytonema sp.					
A0AA91GZ71	Phycocyanin subunit beta	*Scytonema sp. HK-05*	8	2	2
Snowella sp					
A0A2W7BC75	Allophycocyanin	*Snowella sp*	12	2	2
Sphaerospermopsis reniformis					
A0A479ZWB6	Phycocyanin subunit alpha	*Sphaerospermopsis reniformis*	19	2	2
A0A479ZSX1	Phycocyanin, beta subunit	*Sphaerospermopsis reniformis*	8	2	2
Synechococcus sp.					
K9RWE6	Phycocyanin, beta subunit	*Synechococcus sp. (strain ATCC 27167 / PCC 6312)*	8	2	2
A0A1J0PDQ2	Phycocyanin subunit beta	*Synechococcus sp. SynAce01*	12	2	2
Tolypothrix sp.					
A0A218QT84	Phycocyanin beta subunit	*Tolypothrix sp. NIES-4075*	16	3	5
Trichormus variabilis					
Q3M8Z5	Phycocyanin, beta subunit	*Trichormus variabilis (strain ATCC 29413 / PCC 7937)*	12	2	2
A0A3S1CEI7	Allophycocyanin alpha chain	*Trichormus variabilis SAG 1403-4b*	22	3	4
Umezakia ovalisporum					
A0AA43KG12	Phycocyanin subunit beta	*Umezakia ovalisporum FSS-62*	12	2	2
